# Synthesis of novel aryl-substituted 2-aminopyridine derivatives by the cascade reaction of 1,1-enediamines with vinamidinium salts to develop novel anti-Alzheimer agents

**DOI:** 10.1038/s41598-024-64179-1

**Published:** 2024-06-14

**Authors:** Sama Loori, Hormoz Pourtaher, Abdolmohammad Mehranpour, Alireza Hasaninejad, Mohammadreza Eftekharian, Aida Iraji

**Affiliations:** 1 Department of Chemistry, Faculty of Sciences, Persian Gulf University, Bushehr, 75169 Iran; 2 Student Research Committee, Jahrom University of Medical Sciences, Jahrom, Iran; 3 Department of Persian Medicine, School of Medicine, Research Center for Traditional Medicine and History of Medicine, Shiraz University of Medical Sciences, Shiraz, Iran; 4 Stem Cells Technology Research Center, Shiraz University of Medical Sciences, Shiraz, Iran

**Keywords:** Aryl vinamidinium salts, Alzheimer, 1,1-Enediamines, Aryl-substituted 2-aminopyridine, Molecular dynamic simulation, Chemical biology, Drug discovery

## Abstract

Alzheimer’s disease (AD), a severe neurodegenerative disorder, imposes socioeconomic burdens and necessitates innovative therapeutic strategies. Current therapeutic interventions are limited and underscore the need for novel inhibitors of acetylcholinesterase (AChE) and butyrylcholinesterase (BChE), enzymes implicated in the pathogenesis of AD. In this study, we report a novel synthetic strategy for the generation of 2-aminopyridine derivatives via a two-component reaction converging aryl vinamidinium salts with 1,1-enediamines (EDAMs) in a dimethyl sulfoxide (DMSO) solvent system, catalyzed by triethylamine (Et_3_N). The protocol introduces a rapid, efficient, and scalable synthetic pathway, achieving good to excellent yields while maintaining simplistic workup procedures. Seventeen derivatives were synthesized and subsequently screened for their inhibitory activity against AChE and BChE. The most potent derivative, **3m**, exhibited an IC_50_ value of 34.81 ± 3.71 µM against AChE and 20.66 ± 1.01 µM against BChE compared to positive control donepezil with an IC_50_ value of 0.079 ± 0.05 µM against AChE and 10.6 ± 2.1 µM against BChE. Also, detailed kinetic studies were undertaken to elucidate their modes of enzymatic inhibition of the most potent compounds against both AChE and BChE. The promising compound was then subjected to molecular docking and dynamics simulations, revealing significant binding affinities and favorable interaction profiles against AChE and BChE. The in silico ADMET assessments further determined the drug-like properties of **3m**, suggesting it as a promising candidate for further pre-clinical development.

## Introduction

Alzheimer’s disease (AD) is a progressive disorder that typically affects individuals over the age of 65, although early onset can occur at lower ages. Initially, AD impacts the parts of the brain that control memory, thinking, and language skills, and as the disease progresses, individuals may experience confusion, behavioral changes, an inability to carry out daily activities, loss of speech, and social withdrawal^1^.

The pathophysiology of AD is complex, involving multiple systems and processes within the brain. The most accepted mechanisms are the formation of amyloid-beta plaques and neurofibrillary tangles. These proteins are known to have a neurotoxic effect on cholinergic neurons, and the excessive accumulation of these plaques induces oxidative stress, inflammation, and a host of other neurodegenerative processes. These factors collectively contribute to the death of cholinergic neurons^2–4^.

Another significant aspect of AD pathophysiology is the dysfunction in the cholinergic system. The cholinergic system utilizes the neurotransmitter acetylcholine (ACh) to mediate communication related to memory, learning, and other aspects of cognition. ACh signaling is terminated by the enzyme acetylcholinesterase (AChE), which breaks down ACh to prevent excessive activation of receiving neurons^5^. In AD, degeneration of cholinergic neurons, especially those projecting to the cortex and hippocampus, results in inadequate acetylcholine levels. This contributes to the memory and cognitive dysfunction. The progressive deterioration of cholinergic neuronal loss directly correlates with cognitive decline^6^. As a result, enhancing ACh levels provides modest effects on AD symptoms^7,8^.

Butyrylcholinesterase (BChE) is an additional cholinesterase enzyme that shares functional similarities with AChE. Although BChE demonstrates broader substrate specificity, capable of hydrolyzing a range of choline esters, it compensates the role of AChE activity in late-stage AD where the AChE level is decreased. This compensatory role of BChE suggests a potential reserve mechanism for cholinergic neurotransmission regulation^9^.

Currently, there is no cure for AD, and the standard treatment involves using AChE inhibitors, including tacrine, donepezil, and galantamine, to increase the ACh levels. Treatment strategies also need to consider BChE due to its overlapping function and potential role in the disease as the level of AChE at the late stage of AD decreases, and it is proposed that BChE compensates its role in hydrolysis ACh^10,11^.

**Figure 1:**
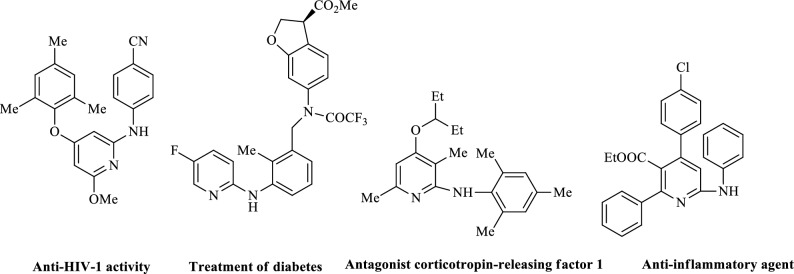
Representative examples of biologically active aryl-substituted 2-aminopyridine derivatives.

2-aminopyridines are a significant group of nitrogen heterocyclic compounds and are commonly found as essential structural elements in biologically active molecules. These kinds of compounds are commonly found in various useful compounds, such as fluorescent organic materials^12^ and therapeutics. Several approaches have been developed to target these heterocycles, including amination reactions^13–15^, Chichibabin aromatic substitution^16^, multicomponent transformations^17,18^, and hetero Diels–Alder reactions^19^. These compounds in different fields exhibit a wide range of biological activities, including inhibition of nitric oxide synthases (NOS)^20^. They can be used as intermediates in the manufacture of drugs^21^, such as piroxicam^22^. They also possess chelating abilities and are utilized as ligands in both organic and inorganic chemistry^23^. Among the derivatives of 2-aminopyridine, aryl-substituted derivatives exhibit a wide range of biological activities, such as allosteric BCRABL1 inhibitor^24^, treatment of neurodegeneration^25^, anticancer^26^, treatment of resistant fungal infections^27^. Some examples of pharmaceutical compounds with aryl-substituted 2-aminopyridine skeletons are shown in Fig. 1^28–30^.

Vinamidinium derivatives are present in natural products such as the betacyanin pigments found in red beets, pokeberry, many cacti, and other plants^31^. They are also present in the porphyrin and corrin ring systems of important compounds like hemoglobin, chlorophyll, vitamin B_12_, and cytochromes^32^. Due to their unique electronic distribution properties, these compounds serve as intriguing and versatile building blocks extensively employed in the synthesis of diverse fused heterocyclic compounds. Consequently, they are important in modern organic synthesis^33–38^.

In continuation of our interest in the preparation of functionalized heterocycles with biological activity, herein we report a novel synthetic strategy for the preparation of aryl-substituted 2-aminopyridine derivatives via the cyclocondensation between 1,1-enediamines (EDAMs) and vinamidinium salts as anti-AD agents.

## Result and discussion

### Designing

**Figure 2:**
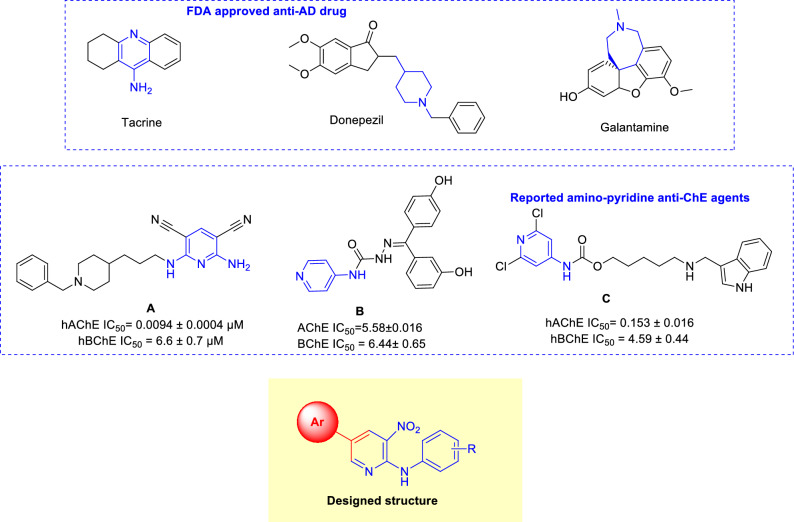
Previously reported ChE inhibitors and newly designed compound.

Nitrogen-containing heterocycles are key in medicinal chemistry, specifically in developing cholinesterase (ChE) inhibitors with anti-AD potential^5^. ChE inhibitors, including tacrine, donepezil, and galantamine (Fig. 2), serve as approved drugs for symptomatic AD relief, primarily owing to their nitrogen-containing moieties that interact effectively with the enzyme’s catalytic anionic site (CAS). This fact underscores the significant role of nitrogen-bearing heterocycles in therapeutic applications. In light of this, ongoing research focuses on synthesizing more potent and efficacious ChE inhibitors using the strategic alteration of template moieties in currently available AD therapeutics. Despite limited reports on nitropyridine, pyridonepezils (**A**, Fig. 2), exhibit inhibitory activity for hAChE similar to that of donepezil, while demonstrating a staggering 703-fold more selectivity against hAChE than hBChE. Molecular modeling supports pyridonepezil dual AChE inhibitory profile, revealing its capacity to bind simultaneously at the enzyme’s catalytic active and peripheral anionic sites (PAS)^39^.

Moreover, compound **B** illustrates a significant cognition-enhancing effect attributable to its potent ChE inhibitory activity^40^. According to molecular docking studies, a series of pyridines (**C**) were reported to show high inhibitory potency against both AChE and BChE. The charged amine group, reminiscent of donepezil, engages in cation-aromatic contact with the CAS residue, Tyr337^41^.

However, amino nitropyridine scaffolds have been somewhat overlooked, necessitating further exploration. In this context, 2-aminopyridines emerge as a particularly beneficial choice, given their potential to interact with both key pockets of ChE, namely, the PAS and CAS. Also, the nitro group in the structure is categorized as the added value, possibly facilitating H-bond interactions with the active site residues of the ChE enzymes. Consequently, a series of aryl-substituted 2-aminopyridines were designed and synthesized. The synthesized derivatives were subsequently evaluated as potential AChE and BChE inhibitors. Furthermore, kinetic studies were conducted on the most potent analogs to ascertain the enzymes’ response. Subsequent molecular docking and molecular dynamic assessments of selected inhibitors were carried out. Finally, the ADMET and drug-likeness properties were studied to predict the potentiality of potent derivatives as viable drug candidates for future studies.

### Chemistry

Initially, to achieve optimal conditions, first, *N*-(2-(4-bromophenyl)-3-(dimethylamino)-allylidene)-*N*-methylmethanaminium perchlorate **1d** (0.5 mmol) and *N*-(4-methoxyphenyl)-2-nitroethene-1,1-diamine **2a** (0.5 mmol) were used as substrates in the model reaction to determine the optimal reaction conditions.

The different types and amounts of solvents, the temperature, and the catalysts were carefully screened, and the results are shown in Table 1. First, the model reaction was tested in different solvents, including EtOH, CH_3_CN, THF, DMF, and DMSO, under heat conditions (Table 1, entries 1–5). As the results show, DMSO was the best solvent and produced the target compound **3j** with a 45% yield (Table 1, entry 5). Second, the different basic catalysts, including Et_3_N, piperidine, NaOMe, and K_2_CO_3_ were evaluated, and it was discovered that Et_3_N was the optimal catalyst to produce the best yields (Table 1, entries 6–9). Third, we performed the reaction at different temperatures in DMSO (Table 1, entries 9–12). The results revealed that the reaction temperature considerably affected the reaction, and the yield of the target compound **3j** was 92% at 80 °C (Table 1, entry 11). Thus, the best reaction conditions for the synthesis of compound **3j** were temperature under 80 °C, for 12h in 1mL DMSO to obtain a product yield of 92% (Table 1, entry 11).

**Table 1:** Optimization of the reaction conditions for the synthesis of **3j**.


Entry	Solvent	Base	T (°C)	Time/h	Yield^a^ (%)
1	EtOH	–	Reflux	12	–
2	CH_3_CN	–	Reflux	12	–
3	THF	–	Reflux	12	–
4	DMF	–	100	12	30
5	DMSO	–	100	12	45
6	DMSO	Piperidine	100	12	65
7	DMSO	K_2_CO_3_	100	12	54
8	DMSO	NaOMe	100	12	70
9	DMSO	Et_3_N	100	12	88
10	DMSO	Et_3_N	120	12	85
11	DMSO	Et_3_**N**	80	12	92
12	DMSO	Et_3_N	60	12	86

Reaction conditions: *N*-(2-(4-bromophenyl)-3-(dimethylamino)-allylidene)-*N*-methylmethanaminium perchlorate **1d** (0.5 mmol), *N*-(4-methoxyphenyl)-2-nitroethene-1,1-diamine **2a** (0.5 mmol), base (1 eq.), solvent (1 mL), 12 h.

^a^Isolated yield.

Once we determined the optimized conditions, as shown in Table 2 we explored different aryl vinamidinium salts **1** and 1,1-enediamines **2** in this protocol. The results showed that the substituents on EDAMs **2** only slightly affected the yield, which did not lead us to find the obvious rules. All EDAMs used are good substrates for the cascade reaction to synthesize new aryl-substituted 2-aminopyridine derivatives. This study applied a range of aryl vinamidinium salts to synthesize aryl-substituted 2-aminopyridine derivatives. As shown in Table 2, aryl vinamidinium salts having halogen (compounds 3c-m) usually produced higher yields than other aryl vinamidinium salts.

**Table 2:** Preparation of new aryl-substituted 2-aminopyridine **3a–q.**


Entry	Reactants	Ar	R	Product	Yield 3 (%)^a^
**1**	**1a + 2a**	H	4-OMe		88
**2**	**1a + 2b**	H	4-Cl		88
**3**	**1b + 2a**	4-F	4-OMe		91
**4**	**1b + 2c**	4-F	4-Me		90
**5**	**1b + 2b**	4-F	4-Cl		94
**6**	**1c + 2a**	4-Cl	4-OMe		94
**7**	**1c + 2b**	4-Cl	4-Cl		95
**8**	**1c + 2d**	4-Cl	1-Me		91
**9**	**1c + 2e**	4-Cl	1-Naphtyl		89
**10**	**1d + 2a**	4-Br	4-OMe		92
**11**	**1d + 2c**	4-Br	4-Cl		93
**12**	**1d + 2b**	4-Br	4-Me		93
**13**	**1e + 2b**		4-Cl		89
**14**	**1f + 2d**	1-Naphtyl	1-Me		87
**15**	**1f + 2c**	1-Naphtyl	4-Me		84
**16**	**1f + 2b**	1-Naphtyl	4-Cl		89
**17**	**1f + 2e**	1-Naphtyl	1-Naphtyl		80

Reaction conditions: 1,1-enediamines (EDAMs), (1 mmol) and aryl vinamidinium salts (1 mmol) in the presence of Et_3_N (1eq) were added to DMSO (1 mL) at 80 °C for 12h, to obtain the desired product **3.**

^a^Isolated yield.

**Scheme 1:**
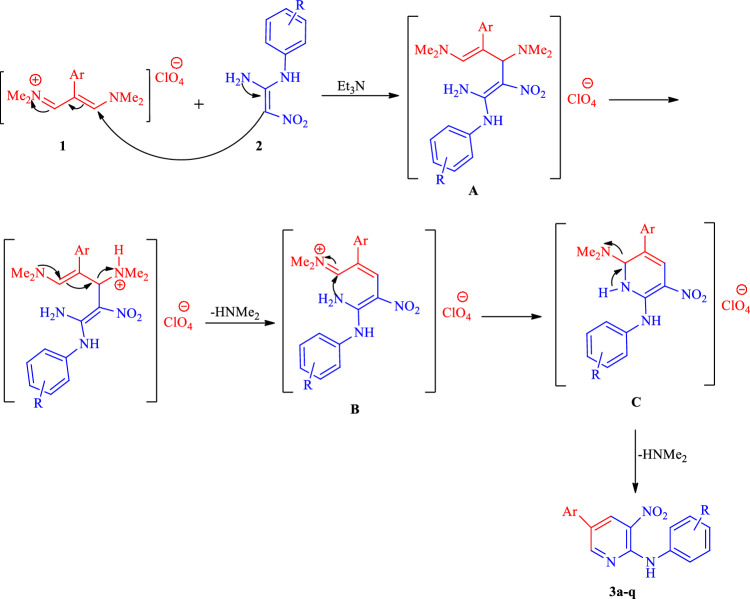
Proposed mechanism for synthesis of compounds **3(a–q).**

Based on the above experimental results, we propose a mechanism in the presence of Et_3_N, which is shown as Scheme 1. First, intermediate **A** is formed by the nucleophilic attack of the amine group in EDAMs **2** to aryl vinamidinium salts **1**. Then, the removal of dimethylamine occurs, followed by the nucleophilic attack of the second molecule of EDAMs on the obtained iminium salt **B** to produce intermediate **C**. Finally, intramolecular nucleophilic attack occurs, and the desired product **3** is formed with the loss of dimethylamine.

### Preliminary structure–activity relationship of AChE inhibition

The current study examined the preliminary structure–activity relationship (SAR) of AChE inhibition by screening seventeen compounds, **3a–q**, compared with a standard AChE inhibitor, donepezil. The results indicated variability in inhibition percentages, highlighting the influence of different substituents at the Ar and R positions (Table 3).

**Table 3:** The anti-AChE activity of **3a–q** expressed in the term of mean ± S.E


Compound	Ar	R	AChE mean ± S.E	
			% inhibition at 50 µM	IC_50_ (µM)
**3a**		4-OMe	28.12 ± 3.93	–
**3b**		4-Cl	59.93 ± 11.04	–
**3c**		4-OMe	10.68 ± 2.31	–
**3d**		4-Me	39.36 ± 1.56	–
**3e**		4-Cl	19.27 ± 3.30	–
**3f**		4-OMe	32.99 ± 1.86	–
**3g**		4-Cl	17.78 ± 2.74	–
**3h**		2-CH_3_	23.11 ± 8.61	–
**3i**		1-Naphthyl	39.72 ± 1.67	–
**3j**		4-OMe	38.83 ± 3.27	–
**3k**		4-Cl	27.76 ± 1.75	–
**3l**		4-Me	24.89 ± 5.01	–
**3m**		4-Cl	74.27 ± 5.86	34.81 ± 3.71
**3n**		2-Me	40.72 ± 2.60	–
**3o**		4-Me	35.58 ± 4.63	–
**3p**		4-Cl	21.43 ± 1.86	–
**3q**		1-Naphthyl	33.99 ± 2.39	–
**Donepezil**^**a**^	–	–	–	0.079 ± 0.05

Data presented here are the mean ± S.E.

^a^Positive control.

Focusing on the phenyl derivatives at the Ar position revealed that substitution with the electron-donating group, such as 4-OMe on compound **3a**, resulted in moderate inhibition (28.12 ± 3.93% at 50 µM). Replacing 4-OMe with an electron-withdrawing and lipophilic 4-Cl on **3b** significantly increased inhibition (59.93 ± 11.04% at 50 µM), suggesting that electron-withdrawing substituent enhance activity.

Further examination of substitutions on the phenyl ring (Ar position) with a small, strong electron-withdrawing substituent like fluorine (**3c–e**) produced diverse results. Compounds **3c** activities dropped with 4-MeO substitution at the R position. Compound **3d**, with a 4-Me substitution at R, demonstrated a three-fold improvement in potency *vs* **3d**. However, derivative **3e** (R = Cl) recorded a reduction in the potency.

Subsequent **3f–i** bearing chlorine at the *para* position of the phenyl ring and varying substituents at the R groups were synthesized. **3f** bearing 4-MeO at R recorded 32.99 ± 1.86% inhibition at 50 µM. In this set, 4-Cl substitution at the R position decreases the potency to 17.78 ± 2.74% inhibition at 50 µM. 2-CH_3_ substitution in **3h** also was not successful in improving the potency. Interestingly, 1-naphthyl substitution (**3i**; bulk and lipophilic group) in this set exhibited better results with 39.72 ± 1.67% inhibition at 50 µM. Intriguingly, larger lipophilic groups seem to have a dual effect. Bulkier groups like 1-naphthyl may enforce conformational restrictions on the enzyme active site, while on the other, they might promote hydrophobic interactions, crucial for stable ligand-enzyme complexes.

The bromine-containing derivatives **3j–l** indicated that bulky electron-donating groups might improve AChE inhibition, with 4-OMe at R (**3j**) outperforming other substituents like 4-Cl (**3k**) and 4-Me (**3l**) in the series, supporting the hypothesis that larger substituents might be beneficial.

Substitution of the phenyl ring with *tert*-butylpyridine (**3m**) led to noteworthy observations regarding the importance of this substitution on inhibition potency. As a nitrogen-containing heterocycle, the pyridine ring brings specific interactions potentially important for AChE affinity. A nitrogen-containing ring such as pyridine suggests that adding a heteroatom into the structure could introduce additional hydrogen bonding interactions or potentially engage in π–π stacking with aromatic residues within the enzyme’s binding site.

To delineate the impact of the *tert*-butylpyridine moiety on AChE inhibition might be through its steric and/or electronic attributes, a series of naphthyl-containing compounds (**3n–q**) were synthesized, revealing variable inhibitory activities. These ranged from a high of 40.72% inhibition with a 2-methyl (**3n**) substituent followed by 4-methyl at R (**3o**; 35.58 ± 4.63% inhibition at 50 µM) > naphtyl at R (**3q**; 33.99 ± 2.39% inhibition at 50 µM) > 4-Cl at R (**3p**; 21.43 ± 1.86% inhibition at 50 µM. The findings suggest that a moderate increase in steric bulk, as provided by methyl groups, enhances inhibition of AChE. In contrast, excessive bulk or specific electronic characteristics, as seen with the naphthyl and chloro groups, might negatively affect the interaction with the enzyme’s active site. These results underscore the delicate balance between hydrophobicity and electron density in AChE inhibitor design.

The study involved the evaluation of nineteen compounds in comparison to the standard AChE inhibitor, donepezil, unveiling varying inhibition percentages and underscoring the impact of electron-donating, electron-withdrawing, and size of substituents on activity. While these derivatives exhibited less potency compared with donepezil, they offer valuable insights into drug design as AChE inhibitors paving the way for the development of more effective compounds for the treatment of AD.

### Preliminary structure–activity relationship of BChE inhibition

The BChE inhibitory activities of a series of derivatives were determined using an Ellman colorimetric method, unveiling the inhibition potencies displayed in Table 4. Specifically, compound **3a** with a phenyl ring at the Ar position and a 4-MeO group at R showcased a 22.07 ± 0.33% inhibition at 50 µM. A subsequent substitution of 4-MeO with 4-Cl in compound **3b** did not significantly enhance BChE inhibition, suggesting a minimal impact of this particular electron-withdrawing group.

**Table 4:** The anti-BChE activity of **3a–q** expressed in the term of mean ± S.E


BChE ± S.E		
Compound	% inhibition at 50 µM	IC_50_ (µM)
**3a**	22.07 ± 0.33	–
**3b**	27.48 ± 1.92	–
**3c**	3.40 ± 1.63	–
**3d**	46.37 ± 2.42	–
**3e**	19.59 ± 1.49	–
**3f**	31.68 ± 1.10	–
**3g**	12.28 ± 0.99	–
**3h**	14.64 ± 1.95	–
**3i**	35.32 ± 5.14	–
**3j**	46.09 ± 0.92	–
**3k**	12.83 ± 1.70	–
**3l**	6.96 ± 0.74	–
**3m**	76.68 ± 6.01	20.66 ± 1.01
**3n**	38.89 ± 2.97	–
**3o**	22.75 ± 2.70	–
**3p**	11.71 ± 0.12	–
**3q**	53.07 ± 0.42	–
**Donepezil**^**a**^	–	10.6 ± 2.1

Data presented here are the mean ± S.E.

^a^Positive control.

Follow-up modifications, including adding a fluorine atom at the *para* position of the phenyl ring, resulted in compound **3d** with a 4-Me at the R position, achieving an appreciable 46.37% inhibition at 50 µM. Conversely, the variations 4-Cl and 4-MeO in compounds **3e** and **3c**, respectively, led to almost inactive outcomes.

Additional evaluations on the derivatives **3f–i**, all integrating a 4-chlorine on the phenyl ring, yielded interesting results. Replacing a *para*-fluorophenyl in **3c** with a *para*-chlorophenyl in **3f** (R = 4-MeO) positively affected the BChE inhibitory activity, while **3g** and **3h** did not manifest substantial improvements. Introducing a naphthyl group at the R position in compound **3i** increased potency to 35.32% inhibition at 50 µM, proposing a key role for bulky and lipophilic groups.

The synthesis of derivatives **3j**with a *para*-bromobenzyl group at Ar further supported the notion that increased bulkiness and lipophilic character at the *para* position of the phenyl ring are essential for potent BChE inhibition when 4-MeO is at the R position. However, additional adjustments yielding compounds **3k** and **3l**, bearing 4-Cl and 4-methyl moieties at R, did not result in favorable outcomes.

Notably, the derivative **3m**, indicated to have *tert*-butylpyridine substituents at the Ar, was highlighted for its superior potency, although the specifics were not provided, hinting at an elevation in potency due to increased bulkiness and the presence of a nitrogen-containing moiety.  This suggests a synergistic effect with the active site of BChE, implying that incorporating such a group can increase inhibitor efficacy.

Lastly, the **3n–q** series, featuring a naphthyl moiety at the Ar position, showed varied activity levels. Here, the **3n** derivative (R = 2-Me) was more active than **3h**, indicating the significance of substituent positioning for BChE inhibition potency. Moving the substituent from *ortho* to *para* significantly reduced the potency to 22.75% inhibition in **3o**, accentuating the importance of spatial configuration in inhibitor design.

In conclusion, **3m** as the most potent analog showed the selectivity ratio for BChE over AChE is 1.686. The SAR study elucidates the complexity of designing BChE inhibitors and highlights the delicate balance that needs to be achieved between hydrophobicity, steric bulk, electronic effects, and precise positioning of substituents. This research not only throws light on the structural considerations critical for BChE inhibitor potency but also emphasizes the potential of nitrogen-containing moieties in enhancing drug efficacy.

### Kinetic study

**Figure 3:**
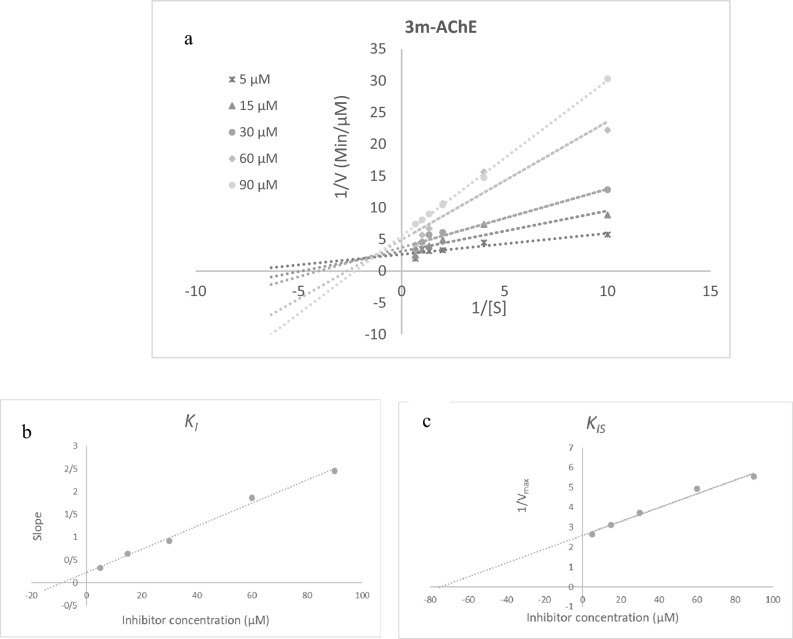
Kinetics of AChE inhibition by **3m**. (**a**) The Lineweaver–Burk plot in the absence and presence of different concentrations of **3m**. (**b**) The secondary plot of *1/V*_*max*_* vs* various concentrations of **3m**. (**c**) The secondary plot of slopes of the lines *vs* various concentrations of **3m.**

To investigate the type of inhibition of AChE and BChE, kinetic studies of **3m** were conducted using various concentrations of ATCI or BTCI as a substrate. As demonstrated in Fig. 3a, the Lineweaver–Burk plot of **3m** showed that the *K*_*m*_ and *V*_*max*_ gradually decreased with increasing the inhibitor concentration, indicating mixed inhibition. The plot of the slope of the lines versus different inhibitor concentrations gave an estimate of the inhibition constant (*K*_*i*_) of 8.92 µM (Fig. 3b). Compound **3m** also recorded the inhibition constant with the enzyme–substrate complex (*K*_*is*_) of 74.97 µM (Fig. 3c).

**Figure 4:**
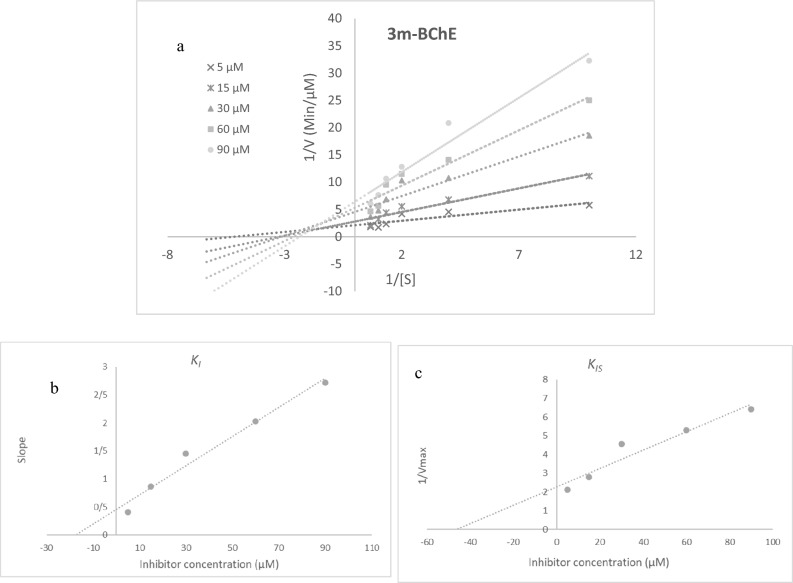
Kinetics of BChE inhibition by **3m**. (**a**) The Lineweaver–Burk plot in the absence and presence of different concentrations of **3m**. (**b**) The secondary plot of *1/Vmax* vs various concentrations of **3m**. (**c**) The secondary plot of slopes of the lines vs various concentrations of **3m.**

Extrapolation of the Lineweavere-Burk reciprocal plot of **3m** yielded the mixed type of inhibition for BChE (Fig. 4a). The plot of slope *vs* different concentrations of inhibitor gave an estimate of *K*_*i*_ of 17.5 µM (Fig. 4b) and the plot of 1/V_max_ *vs* different concentrations of **3m** gave *K*_*is*_ value of 46.27 µM (Fig. 4c).

### Molecular docking studies

The X-ray crystallographic structure of AChE illustrates the distinct binding sites crucial for its function. The PAS located at the entrance of the gorge is composed of key residues like Tyr70, Asp72, Tyr121, Trp279, and Tyr334, while the CAS at the bottom of the gorge contains the catalytic triad comprising Ser200, His440, and Glu327, alongside the catalytic anionic site close to the triad, including Trp84, Tyr130, Gly199, His441, and His444. Additionally, residues like Trp86, Glu220, and Trp337 within the choline-binding site further underscore AChE’s structural and functional features.

In contrast, BChE boasts a larger active site compared to AChE, accommodating more bulk ligands. The PAS of BChE includes residues Asp70 and Tyr332, while the catalytic triad on the enzyme’s active site features Ser198, His438, and Glu325. Additionally, the catalytic center is occupied by residues Gly116, Gly117, and Ala199, highlighting the differences in the binding sites between AChE and BChE.

Molecular docking analysis using the Schrödinger software suite was employed to investigate the interaction capabilities of compound **3m** against AChE and BChE. The selection of PDB IDs is significantly influenced by factors such as resolution and structural integrity. X-ray crystallography is the preferred method for determining protein structures. Additionally, considering the human source of the enzyme is essential, as it may provide insights specific to human biology relevant to the research. As a result in the current study AChE (4EY7) and BChE (4BDS) were chosen.

Before the analysis of compound **3m**, the molecular docking protocol was validated by re-docking the co-crystallized ligand within the binding site of the respective enzymes. The re-docking procedure yielded a root-mean-square deviation (RMSD) of less than 2 Å for the lowest binding energy pose, confirming the reliability of the docking methodology.

**Figure 5:**
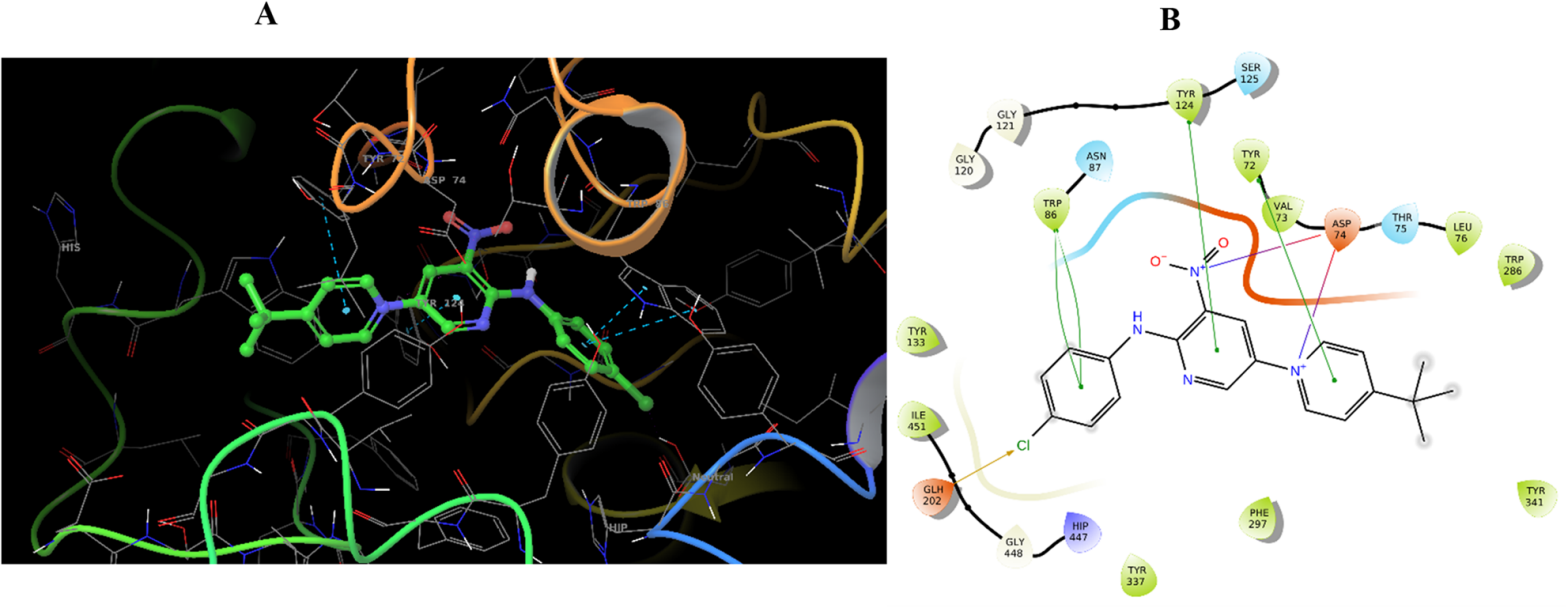
Molecular docking analysis of **3m** compounds against AChE. 3D interactions of **3 m** (**A**), 2D interactions of **3m** (**B**).

Figure 5 outlines the binding interactions of the potent inhibitor **3m** within the catalytic gorge of AChE. This compound engaged in a bifurcated salt bridge bonding network with Asp74, one bond originating from the nitro group of nitropyridine and the other anchored through the amine functionality of 4-chloroaniline. In addition to hydrogen bonding, compound **3m** participated in π–π stacking interactions; one interaction involved nitropyridine and the aromatic ring of Tyr124, while another coupled pyridinium with Tyr72 of PAS. Further, two π–π stacking interactions were observed, one with Trp86 (choline binding site) and another involving 4-chloroaniline, reinforcing the stability of the ligand-enzyme complex. Moreover, the chlorine group in 4-chloroaniline facilitated a halogen bond interaction with Glu202, which may contribute to the specificity and affinity of **3m** towards AChE. These diverse non-covalent interactions underscore the structural properties driving the inhibitory potency of compound **3m** against AChE.

**Figure 6:**
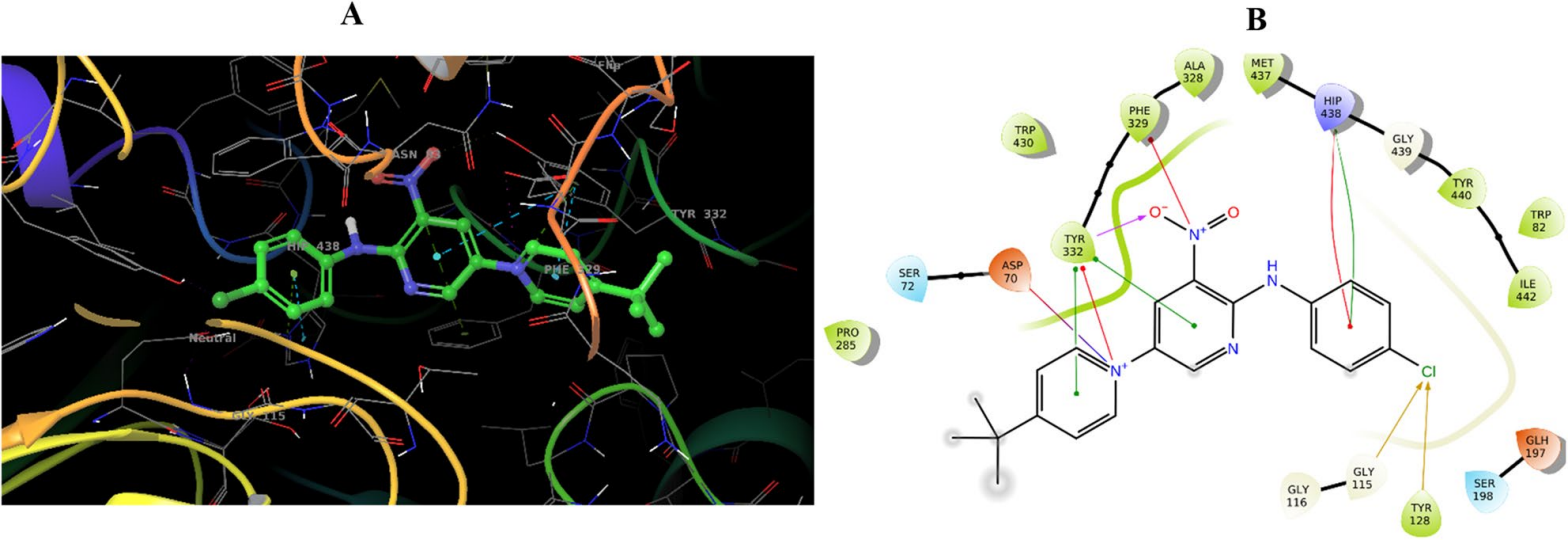
Molecular docking analysis of **3m** compounds against BChE. 3D interactions of **3m** (**A**), 2D interactions of **3m** (**B**).

In the active site of BChE, the interaction profile of compound **3m** was analyzed (Fig. 6). The molecular docking revealed that the pyridinium moiety forms a salt bridge with Asp70 of PAS pocket, a key residue in the enzyme’s active site. This interaction is crucial as it potentially stabilizes the ligand within the binding pocket through electrostatic complementarity. Additional interactions included a pi–cation bond with Tyr332 of PAS pocket and a pi–pi stacking interaction with the same tyrosine residue, delineating a significant aromatic interaction network contributing to **3m**’s binding affinity. In the central region of the ligand structure, the nitropyridine group formed a hydrogen bond with Tyr332 of PAS pocket, facilitating strong ligand–protein binding. This was further complemented by a pi–cation interaction with Phe329 and an additional pi–pi stacking interaction with Tyr332, suggesting that the nitropyridine ring system is key to the binding affinity given its engagement with multiple aromatic side chains of the enzyme. The 4-chloroaniline moiety interacted through pi–cation and pi–pi stacking interactions with the imidazole ring of His438 located within the catalytic triad of the enzyme. This region was specified and is a key point of interaction within the enzyme’s structure. The chlorine atom of this group engaged in halogen bonding with Gly115 oxanion hole and Tyr128, providing additional anchorage points. These halogen bonds are significant as they contribute to the positioning and orientation of the ligand within the enzyme’s binding site, potentially improving the specificity and inhibitory potency of compound **3m** against BChE.

Overall, the intricate network of electrostatic and aromatic interactions and halogen bond formations elucidate the strong binding affinity of compound **3m** for BChE and provide insights into its potent inhibitory activity. These detailed interactions facilitate a deeper understanding of the compound’s mechanism of inhibition, presenting a comprehensive profile that can inform further structural optimization for enhanced therapeutic potential.

### Molecular dynamic simulations

**Figure 7:**
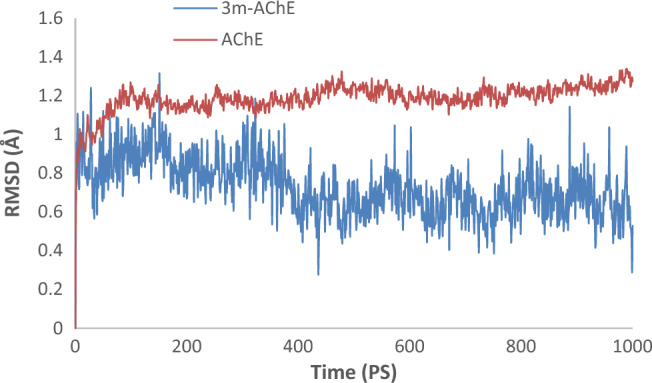
RMSD of AChE and **3m**-AChE.

Molecular dynamics (MD) simulations were carried out to analyze the stability of the AChE and BChE with compound **3m** complex, compared to their unbound forms. The RMSD was specifically monitored to assess structural consistency. In the case of apo AChE, RMSD values rose gradually in the initial phase, signifying typical conformational adjustment, and eventually reached a stable average of 1.2 Å. In contrast, the complex of **3m** with AChE displayed rapid stabilization in the simulations, with the RMSD plateauing at a significantly lower value of 0.6 Å in less than 20 nanoseconds. This behavior suggests that compound **3m** binds effectively to AChE, creating a stable complex that could behave as a potent inhibitor due to the low RMSD, indicating a strong interaction and high structural rigidity of the enzyme-inhibitor complex (Fig. 7).

**Figure 8:**
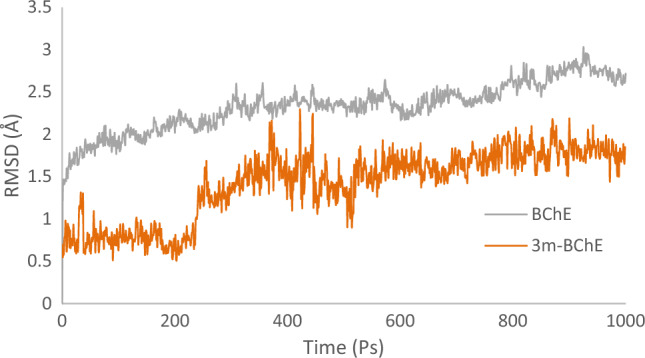
RMSD of BChE and **3m**-BChE.

Following the analysis of AChE interactions with compound **3m**, similar procedures were undertaken to assess the stability of BChE in a complex with **3m**. Initially, the RMSD of the **3m**-BChE complex was observed to maintain a stable course up to 230 picoseconds. However, after this duration, an increase in RMSD was noted, peaking at approximately 1.5 Å, sustained until the end of the simulation time, suggesting a delayed destabilization compared to the earlier stabilization observed in the AChE complex. On the other hand, the apo BChE enzyme demonstrated a higher RMSD value of 2.5 Å. The difference in the RMSD values between the apo BChE and the ligand-bound BChE indicates that the binding of compound **3m** influences the overall stability of the BChE (Fig. 8).

**Figure 9:**
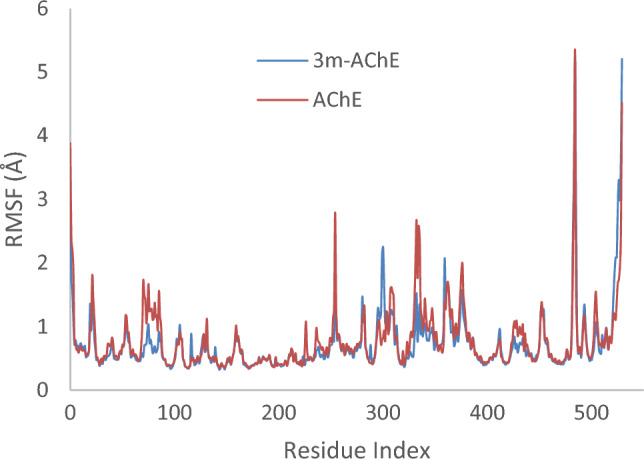
RMSF plot of the **3m**-AChE and AChE residue of MD simulations within 100 ns at 300 K of AChE.

Subsequently, alterations in Root Mean Square Fluctuation (RMSF) were scrutinized for **3m**-AChE as compared to the AChE. The RMSF profile of **3m**-AChE, as depicted in Fig. 7, manifests reduced variability within certain residues, which is attributed to the interaction carried out by the **3m** moiety at AChE’s active site. This modification notably reduces dynamic fluctuations in residues 67–96 of PAS pocket, 198–271, and 320–357 of PAS pocket. The findings suggest that the **3m** moiety stabilizes certain enzyme regions by possibly forming non-covalent interactions and altering the flexibility while also triggering decreased mobility in other domains, which could affect the enzyme’s ability to hydrolyze substrates (Fig. 9).

**Figure 10:**
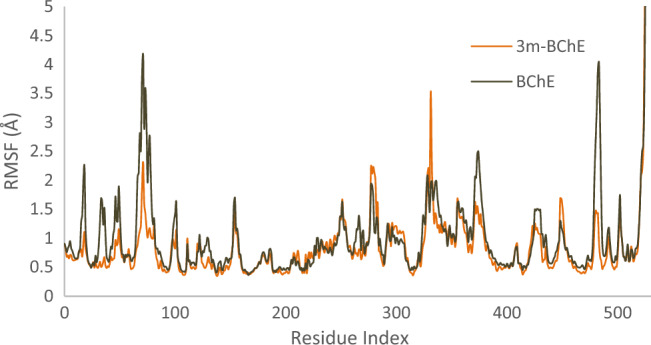
RMSF plot of the **3m**-BChE and BChE residue of MD simulations within 100 ns at 300 K of BChE.

Similarly, it was observed that the presence of **3m** notably reduced the RMSF within BChE (Fig. 10). The significant decrease in RMSF indicates that **3m** stabilizes BChE, particularly in interactions with residues spanning 61–95, which constitute PAS pocket, and residues 471 to 496. This interaction suggests that **3m**’s binding predominantly affects critical regions involved in the enzyme’s active conformation and dynamics.

**Figure 11:**
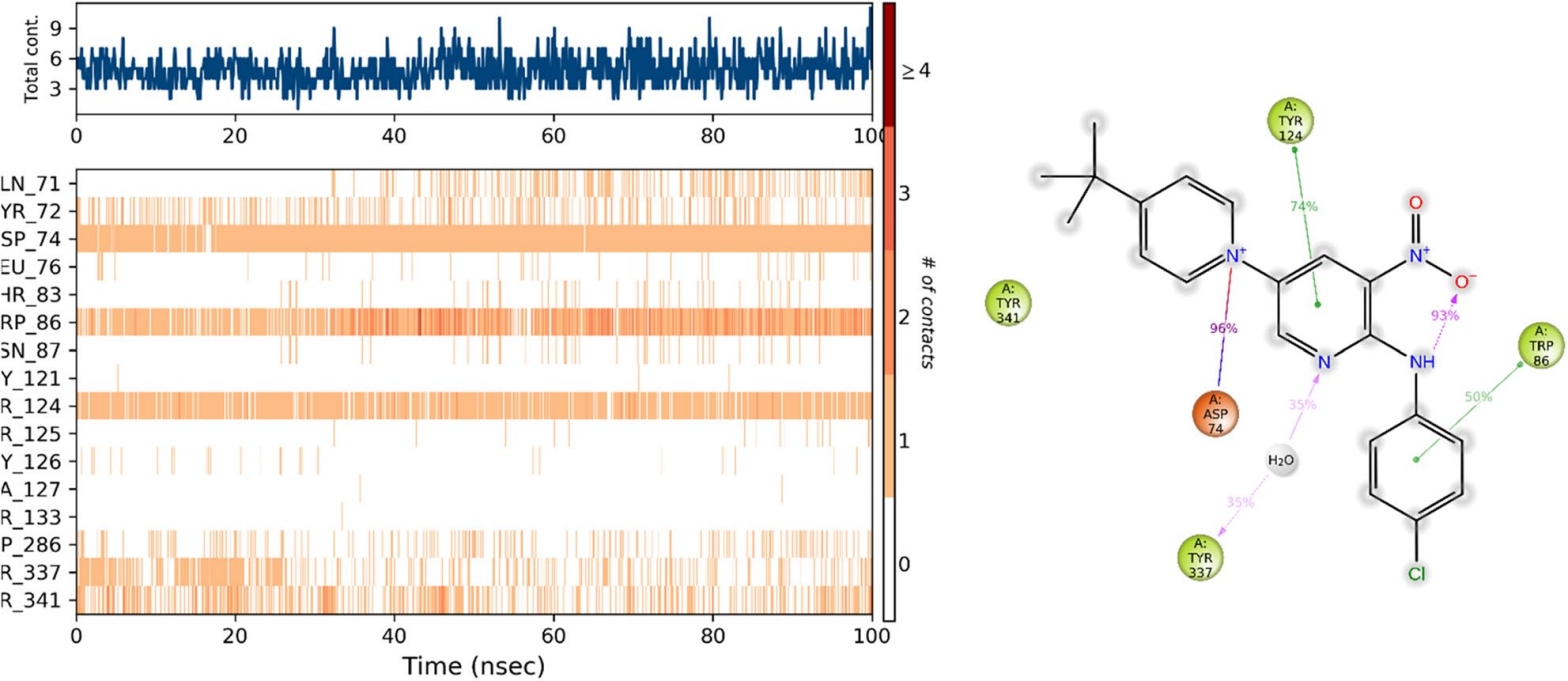
Timeline interactions timeline of **3m** with the active site of the AChE. (**b**) 2D representation of **3m**-residue interactions that occur more than 30.0% of the simulation time.

Moreover, the analysis of the interaction fraction plot (Fig. 11) provided insights into the chemical characteristics of the interactions between **3m** and the AChE binding site. The plot implies that **3m** predominantly engages in ionic interactions with the Asp74 residue, facilitates π–π stacking interactions with Trp86 and Tyr124, and partakes in a water-mediated hydrogen bonding with Tyr337. These findings highlight the nature of the binding specificity of **3m** to the AChE active site, which is largely obsessed by these diverse types of molecular interactions.

**Figure 12:**
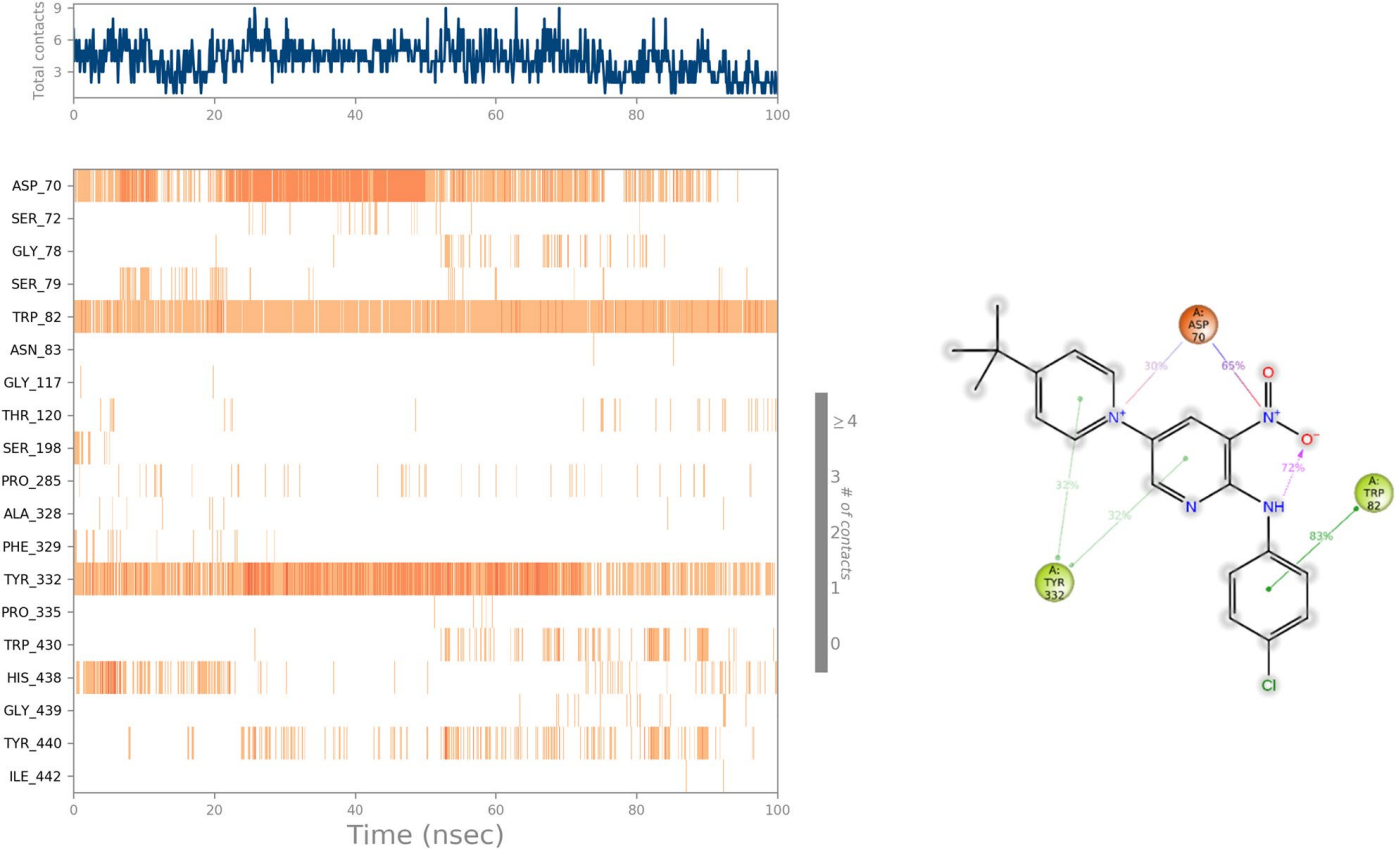
Timeline interactions timeline of **3m** with the active site of the BChE. (**b**) 2D representation of 3m-residue interactions that occur more than 30.0% of the simulation time.

Trajectory assessments of **3m**-BChE revealed that Asp70 is integral to the binding dynamics of **3m** within the active site of the target enzyme. It involves two distinct ionic interactions: one with the nitro group observed in 65% of the MD simulation and the other with the pyridine ring, occurring in 30% of the MD study. In addition, the phenyl and pyridine rings of **3m**, acting as electron-rich regions, enter into π-π stacking interactions with Trp82 and Tyr332. These interactions enhanced the stability of the BChE-inhibitor complex and improved the potency of **3m** as an inhibitor (Fig. 12).

### Molecular mechanics Poisson–Boltzmann surface area (MM/PBSA) calculations

The MM/PBSA technique is often employed for calculating ligands’ relatively accurate binding free energies with their targets, considering the conformational fluctuations and entropic contributions derived from MD trajectories. This computational method is particularly useful for screening potential inhibitors by evaluating their binding affinities. Utilizing the most stable trajectories obtained from MD simulations, MM/PBSA calculations were carried out to assess the binding energy components. The results reported in Table 5 highlight the substantial contribution of van der Waals interactions to the binding energy of **3m** *vs* donepezil, underscoring its strong interaction and potential as an effective inhibitor.

**Table 5:** Different MM/PBSA terms for the relative change in the enthalpy of the AChE and BChE binding site in complex with **3m** inhibitor at 300 K.

Energy term (kcal/mol)	dG bind Lipo	dG bind vdW	dG binding
3m-AChE	− 23.76	− 51.70	− 65.67
3m-BChE	− 22.52	− 44.78	− 56.44
Donepezil-AChE	− 41.52	− 58.47	− 79.68
Donepezil -BChE	− 30.69	− 45.55	− 69.25

### In silico drug-likeness, ADME, and toxicity studies

**Figure 13:**
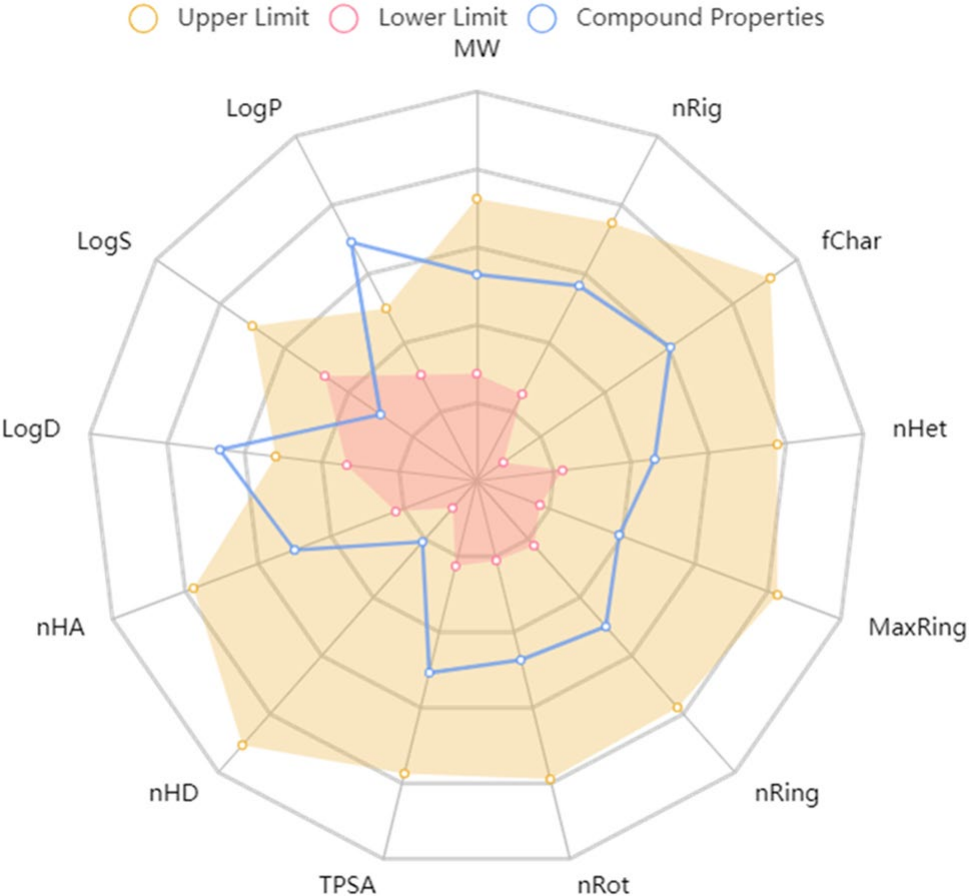
ADMET radar calculation of compound **3m**.

The drug-likeness and ADME/T (absorption, distribution, metabolism, excretion, and toxicity) properties of the most potent compounds within the **3m** series were computationally evaluated to estimate their potential as orally administered agents. The results of these in silico predictions were presented in Table 6 and Fig. 13. The data indicated that the compound, **3m**, has the desirable range for properties that define an ideal oral therapeutic agent and mostly fulfilling criteria such as bioavailability, stability, and non-toxicity that are encompassed within the concept of drug-like characteristics.

**Table 6:** Physicochemical properties of compound **3m**.

	Parameters	Value		Parameters	Value
Physicochemical property	Molecular Weight	383.130	Distribution	VD	1.928
	Volume	380.539		Fraction unbounded plasma	1.026%
	Density	1.007	Metabolism	CYP1A2 inhibitor	+
	nHA	6		CYP1A2 inhibitor	+
	nHD	1		CYP2C9 inhibitor	−
	nRot	5		CYP2C9 substrate	−
	nRing	3		CYP2D6 inhibitor	+
	MaxRing	6		CYP2D6 substrate	−
	nHet	7	Excretion	CL	Excretion
	fChar	1		T1/2	
	TPSA	71.940	Toxicity	AMES toxicity	−
	logP	5.985		H-HT	−
	logD	4.568		Rat oral acute toxicity	−
Absorption	Caco-2 permeability	− 4.520		Carcinogencity	−
	MDCK permeability	5e−05		Skin sensitization	+
	Pgp-inhibitor	+		Acute toxicity rule	0
	Pgp-substrate	+	Medicinal chemistry	SAscore	2.807
	HLA	> 90%		Lipinski rule	+

## Conclusion

In conclusion, we have developed a procedure for the simple synthesis of a variety of potentially biologically active aryl-substituted 2-aminopyridine through a cascade reaction. This reaction provides a novel, rapid, and efficient route for preparing a variety of aryl-substituted 2-aminopyridine in good to excellent yields from readily available building blocks of aryl vinamidinium salts **1** and EDAMs **2**. Next, twenty derivatives were developed, and among these derivatives, compound **3m** demonstrated the highest potency against both AChE and BChE with IC_50_ values of 34.81 ± 3.71 and 20.66 ± 1.01. The kinetic study of **3m** demonstrated mix-type inhibition against both enzymes and demonstrated *K*_*i*_ = 8.92 µM and *K*_*is*_ = 74.97 µM against AChE and *K*_*i*_ = 17.5 µM and *K*_*is*_ = 46.27 µM against BChE. Subsequent docking studies and MD simulations provided a deeper understanding of **3m** binding interactions within the active sites of AChE and BChE, illustrating affinity for critical enzymatic pockets and reinforcing its potential as a therapeutic agent. In silico ADME-T profiling validated the ideal drug-likeness properties of **3m**. Conclusively, compound **3m** has emerged as an ideal candidate in the search for effective AD management drugs. The promising results from our synthetic, biological, and computational studies indicate further research to explore its clinical applicability and elucidate the underlying mechanisms.

## Experimental

### Chemicals and apparatus

All chemicals were purchased from Merck or Fuluka chemical companies. ^1^H-NMR 300 MHz and ^13^C-NMR 75 MHz spectra were run on a Bruker Avance 300 MHz instrument in DMSO-d_6_. Melting points were recorded as a Buchi B-545 apparatus in open capillary tubes. Reaction progress was screened by TLC using silica gel polygram SIL G/UV254 plates.

### General procedure for the synthesis of compounds 3a–r

A mixture of aryl vinamidinium salts **1** (1 mmol) and EDAMs **2** (1 mmol) were stirred in the presence of Et_3_N (1 eq) in 1 mL of DMSO at 80 °C for 12h. The completion of reaction was monitored by TLC. After completion of the reaction, water was added to the reaction mixture to result in a crude participation which filtered and washed by isopropanol (5 ml) and then dried to obtain the pure product (Fig. S1).

#### N-(4-Methoxyphenyl)-3-nitro-5-phenylpyridin-2-amine (3a)

Red powder, Yield: 88%, m.p: 152–154 °C; (TLC; hexane–EtOAc, 5:1, R_f_ = 0.364); ^1^H NMR (300 MHz, DMSO-d_6_) δ: 9.96 (1H, s, NH), 8.86 (1H, d, *J* = 2.4 Hz, Ar), 8.71 (1H, d, *J* = 2.4 Hz, Ar), 7.81–7.71 (2H, m, Ar), 7.59–7.38 (5H, m, Ar), 7.00–6.96 (2H, m, Ar), 3.79 (3H, s, OMe); ^13^C NMR (75 MHz, DMSO-d_6_) δ: 156.8, 153.7, 149.6, 135.6, 132.8, 131.5, 129.6, 128.7, 128.3, 126.6, 126.3, 125.6, 114.2, 55.7; Anal. calcd for: C, 67.28; H, 4.71; N, 13.08. Found: C, 67, 75; H, 4.33; N, 13.68.

#### N-(4-Chlorophenyl)-3-nitro-5-phenylpyridin-2-amine (3b)

Red powder, Yield: 88%, m.p: 158–160 °C; (TLC; hexane–EtOAc, 5:1, R_f_ = 0.368); ^1^H NMR (300 MHz, DMSO-d_6_) δ: 10.04 (1H, s, NH), 8.91 (1H, d, *J* = 2.4 Hz, Ar), 8.74 (1H, d, *J* = 2.4 Hz, Ar), 7.85–7.38 (10H, m, Ar); ^13^C NMR (75 MHz, DMSO-d_6_) δ: 153.1, 148.6, 137.9, 136.2, 135.4, 132.9, 129.6, 128.9, 128.4, 128.3, 127.3, 126.7, 124.8; Anal. calcd for: C, 62.68; H, 3.71; N, 12.90. Found: C, 62.69; H, 3.67; N, 12.96; MS (*m/z*): 325 [M^+^], 278, 244, 216, 149, 113, 75.

#### 5-(4-Fluorophenyl)-N-(4-methoxyphenyl)-3-nitropyridin-2-amine (3c)

Brown powder, Yield: 91%, m.p: 175–177 °C; (TLC; hexane–EtOAc, 5:1, R_f_ = 0.360); ^1^H NMR (300 MHz, DMSO-d_6_) δ 9.95 (1H, s, NH), 8.84 (1H, t, *J* = 2.1 Hz, Ar), 8.70 (1H, t, *J* = 2.1 Hz, Ar), 7.87–7.75 (2H, m, Ar), 7.54 (2H, dt, *J* = 10.6, 2.7 Hz, Ar), 7.34 (2H, dt, *J* = 8.8, 4.3 Hz, Ar), 7.03–6.92 (2H, m, Ar), 3.79 (3H, s, OMe); ^13^C NMR (75 MHz, DMSO-d_6_) δ: 160.8, 156.8, 153.7, 149.5, 132.8, 132.1, 131.5, 128.8, 128.7, 128.6, 125.6, 125.4, 116.6, 116.3, 114.2, 55.7; Anal. calcd for: C, 63.71; H, 4.16; N, 12.38. Found: C, 63.41; H, 4.29; N, 12.62.

#### 5-(4-Fluorophenyl)-3-nitro-N-(p-tolyl)pyridin-2-amine (3d)

Red powder, Yield: 90%, m.p: 152–154 °C; (TLC; hexane–EtOAc, 5:1, R_f_ = 0.364); ^1^H NMR (300 MHz, DMSO-d_6_) δ: 9.98 (1H, s, NH), 8.85 (1H, d, *J* = 2.4 Hz, Ar), 8.70 (1H, d, *J* = 2.3 Hz, Ar), 7.81 (2H, dd, *J* = 8.5, 5.3 Hz, Ar), 7.56 (2H, d, *J* = 8.0 Hz, Ar), 7.33 (2H, t, *J* = 8.6 Hz, Ar), 7.20 (2H, d, *J* = 8.0 Hz, Ar), 2.33 (3H, s, Me); ^13^C NMR (75 MHz, DMSO-d_6_) δ: 160.9, 153.4, 149.1, 136.1, 133.9, 132.8, 132.1, 129.5, 128.9, 128.8, 128.7, 125.7, 123.4, 116.6, 116.3, 21.0; Anal. calcd for: C, 66.87; H, 4.36; N, 13.00. Found: C, 66.58; H, 4.42; N, 12.85; MS (*m/z*): 323 [M^+^], 276, 234, 158, 133, 91, 65.

#### N-(4-Chlorophenyl)-5-(4-fluorophenyl)-3-nitropyridin-2-amine (3e)

Red powder, Yield: 94%, m.p: 196–198 °C; (TLC; hexane–EtOAc, 5:1, R_f_ = 0.365); ^1^H NMR (300 MHz, DMSO-d_6_) δ: 10.01 (1H, s, NH), 8.87 (1H, d, *J* = 2.3 Hz, Ar), 8.71 (1H, d, *J* = 2.4 Hz, Ar), 7.86–7.69 (4H, m, Ar), 7.47–7.27 (5H, m, Ar); ^13^C NMR (75 MHz, DMSO-d6) δ: 160.9, 153.0, 148.5, 137.8, 132.9, 132.0, 131.9, 129.5, 128.9, 128.9, 128.8, 128.3, 126.3, 124.8, 116.6, 116.3; Anal. calcd for: C, 59.40; H, 3.23; N, 12.22. Found: C, 59.33; H, 3.56; N, 12.47; MS (*m/z*): 343 [M^+^], 296, 262, 234, 207, 158, 131, 75.

#### 5-(4-Chlorophenyl)-N-(4-methoxyphenyl)-3-nitropyridin-2-amine (3f)

Red powder, Yield: 94%, m.p: 170–172 °C; (TLC; hexane–EtOAc, 5:1, R_f_ = 0.360); ^1^H NMR (300 MHz, DMSO-d_6_) δ 9.97 (1H, s, NH), 8.86 (1H, d, *J* = 2.4 Hz, Ar), 8.73 (1H, d, *J* = 2.4 Hz, Ar), 7.86–7.75 (2H, m, Ar), 7.60–7.47 (4H, m, Ar), 7.04–6.89 (2H, m, Ar), 3.79 (3H, s, OMe); ^13^C NMR (75 MHz, DMSO) δ: 156.9, 153.6, 149.7, 134.5, 133.1, 132.9, 131.4, 129.5, 128.6, 128.3, 125.6, 125.5, 125.0, 114.2, 55.7; Anal. calcd for: C, 60.77; H, 3.97; N, 11.81. Found: C, 60.89; H, 4.05; N, 11.49; MS (*m/z*): 355 [M^+^], 294, 265, 229, 174, 140, 115, 92, 64.

#### N,5-Bis(4-chlorophenyl)-3-nitropyridin-2-amine (3g)

Red powder, Yield: 95%, m.p: 167–169 °C; (TLC; hexane–EtOAc, 5:1, R_f_ = 0.365); ^1^H NMR (300 MHz, DMSO-d_6_) δ: 10.05 (1H, s, NH), 8.92 (1H, d, *J* = 2.4 Hz, Ar), 8.77 (1H, d, *J* = 2.4 Hz, Ar), 7.87–7.70 (4H, m, Ar), 7.61–7.42 (4H, m, Ar); ^13^C NMR (75 MHz, DMSO-d6) δ: 153.1, 148.8, 137.8, 134.4, 133.3, 133.1, 129.6, 129.6, 128.9, 128.5, 128.4, 126.0, 125.0; Anal. calcd for: C, 56.69; H, 3.08; N, 11.67. Found: C, 56.19; H, 3.13; N, 11.63.

#### 5-(4-Chlorophenyl)-3-nitro-N-(o-tolyl)pyridin-2-amine (3h)

Brown powder, Yield: 94%, m.p: 124–126 °C; (TLC; hexane–EtOAc, 5:1, R_f_ = 0.368); ^1^H NMR (300 MHz, DMSO-d_6_) δ: 9.94 (1H, s, NH), 8.83 (1H, d, *J* = 2.4 Hz, Ar), 8.73 (1H, d, *J* = 2.4 Hz, Ar), 7.81–7.67 (3H, m, Ar), 7.52 (2H, d, *J* = 8.5 Hz, Ar), 7.35–7.14 (3H, m, Ar), 2.27 (3H, s, Me); ^13^C NMR (75 MHz, DMSO-d6) δ: 153.8, 149.9, 137.3, 134.5, 133.1, 132.9, 132.8, 130.8, 129.5, 128.7, 128.3, 126.7, 126.0, 125.0, 18.3; Anal. calcd for: C, 63.63; H, 4.15; N, 12.37. Found: C, 63.58; H, 4.29; N, 12.44.

#### 5-(4-Chlorophenyl)-N-(naphthalen-1-yl)-3-nitropyridin-2-amine (3i)

Red powder, Yield: 89%, m.p: 150–152°C; (TLC; hexane–EtOAc, 5:1, R_f_ = 0.368); ^1^H NMR (300 MHz, DMSO-d_6_) δ: 10.34 (1H, s, NH), 8.75 (2H, dd, *J* = 14.7, 2.4 Hz, Ar), 8.05–7.88 (3H, m, Ar), 7.80–7.73 (3H, m, Ar), 7.63–7.49 (5H, m, Ar); ^13^C NMR (75 MHz, DMSO-d6) δ: 153.7, 151.0, 134.8, 134.5, 134.3, 133.1, 132.8, 130.0, 129.5, 128.9, 128.7, 128.3, 126.8, 126.8, 126.6, 126.2, 125.1, 124.3, 123.2, 40.8; Anal. calcd for: C, 67.12; H, 3.75; N, 11.18. Found: C, 67.49; H, 3.60; N, 11.38; MS (*m/z*): 375 [M^+^], 328, 293, 264, 190, 163, 111, 75.

#### 5-(4-Bromophenyl)-N-(4-methoxyphenyl)-3-nitropyridin-2-amine (3j)

Red powder, Yield: 92%, m.p: 164–166 °C; (TLC; hexane–EtOAc, 5:1, R_f_ = 0.360); ^1^H NMR (301 MHz, DMSO-d_6_) δ: 9.98 (1H, s, NH), 8.85 (1H, s, Ar), 8.73 (1H, s, Ar), 7.80–7.48 (6H, m, Ar), 6.98 (2H, d, *J* = 8.3 Hz, Ar), 3.79 (3H, s, OMe); ^13^C NMR (75 MHz, DMSO) δ: ^13^C NMR (75 MHz, DMSO-d6) δ: 157.7, 153.6, 149.7, 134.9, 132.8, 132.4, 131.4, 128.6, 125.6, 125.0, 121.6, 114.2, 55.7; Anal. calcd for: C, 54.02; H, 3.53; N, 10.50. Found: C, 53.94; H, 3.64; N, 10.33; MS (*m/z*): 399 [M^+^], 354, 311, 274, 229, 183, 149, 123, 92, 64.

#### 5-(4-Bromophenyl)-N-(4-chlorophenyl)-3-nitropyridin-2-amine (3k)

Red powder, Yield: 93%, m.p: 174–176 °C; (TLC; hexane–EtOAc, 5:1, R_f_ = 0.365); ^1^H NMR (300 MHz, DMSO-d_6_) δ: 10.05 (1H, s, NH), 8.90 (1H, d, *J* = 2.3 Hz, Ar), 8.75 (1H, d, *J* = 2.4 Hz, Ar), 7.80–7.65 (6H, m, Ar), 7.50–7.39 (2H, m, Ar); ^13^C NMR (75 MHz, DMSO-d6) δ: 153.07, 132.52, 128.94, 128.84, 125.02; Anal. calcd for: C, 50.46; H, 2.74; N, 10.38. Found: C, 50.68; H, 2.82; N, 10.49; MS (*m/z*): 405 [M^+^], 357, 324, 278, 242, 214, 140, 113, 75.

#### 5-(4-Bromophenyl)-3-nitro-N-(p-tolyl)pyridin-2-amine (3l)

Red powder, Yield: 93%, m.p: 163–165 °C; (TLC; hexane–EtOAc, 5:1, R_f_ = 0.364); ^1^H NMR (300 MHz, DMSO-d_6_) δ: 10.01 (1H, s, NH), 8.90 (1H, d, *J* = 2.4 Hz, Ar), 8.75 (1H, d, *J* = 2.4 Hz, Ar), 7.88–7.77 (2H, m, Ar), 7.56 (4H, d, *J* = 8.2 Hz, Ar), 7.22 (2H, d, *J* = 8.0 Hz, Ar), 2.33 (3H, s, Me); ^13^C NMR (75 MHz, DMSO-d6) δ: 153.5, 149.3, 136.1, 134.5, 134.0, 133.1, 132.9, 129.5, 129.0, 128.4, 125.3, 123.5, 21.0; Anal. calcd for: C, 56.27; H, 3.67; N, 10.94. Found: C, 56.96; H, 3.42; N, 11.06.

#### 4-(Tert-butyl)-6ʹ-((4-chlorophenyl)amino)-5'-nitro-[1,3'-bipyridin]-1-ium perchlorate (3m)

Red powder, Yield: 89%, m.p: 268–270 °C; (TLC; hexane–EtOAc, 5:1, R_f_ = 0); ^1^H NMR (300 MHz, DMSO-d_6_) δ: 10.30 (1H, s, NH), 9.24 (2H, d, *J* = 6.6 Hz, Ar), 9.17 (1H, d, *J* = 2.6 Hz, Ar), 8.91 (1H, d, *J* = 2.6 Hz, Ar), 8.36 (2H, d, *J* = 6.6 Hz, Ar), 7.72–7.59 (2H, m, Ar), 7.55–7.44 (2H, m, Ar), 1.44 (9H, s, Ar); ^13^C NMR (75 MHz, DMSO-d6) δ: 171.8, 150.9, 150.1, 144.8, 137.2, 133.1, 130.1, 129.5, 129.1, 129.0, 128.2, 126.0, 126.0, 125.4, 37.0, 29.9; Anal. calcd for: C, 49.70; H, 4.17; N, 11.59. Found: C, 49.95; H, 4.39; N, 11.13.

#### 5-(Naphthalen-1-yl)-3-nitro-N-(o-tolyl)pyridin-2-amine (3n)

Red powder, Yield: 87%, m.p: 134–136 °C; (TLC; hexane–EtOAc, 5:1, R_f_ = 0.372); ^1^H NMR (300 MHz, DMSO-d_6_) δ: 10.01 (1H, s, NH), 8.65–8.55 (2H, m, Ar), 8.09–7.75 (4H, m, Ar), 7.59 (4H, ddt, *J* = 12.2, 7.6, 4.4 Hz, Ar), 7.41–7.10 (3H, m, Ar), 2.34 (3H, s, Me); ^13^C NMR (75 MHz, DMSO-d6) δ: 156.1, 150.0, 137.4, 136.1, 134.3, 133.9, 132.9, 131.3, 130.8, 129.0, 128.9, 128.4, 128.0, 127.4, 126.7, 126.1, 126.1, 126.0, 125.1, 18.4; Anal. calcd for: C, 74.35; H, 4.82; N, 11.82. Found: C, 74.43; H, 4.72; N, 11.67.

#### 5-(Naphthalen-1-yl)-3-nitro-N-(p-tolyl)pyridin-2-amine (3o)

Brown powder, Yield: 84%, m.p: 137–139 °C; (TLC; hexane–EtOAc, 1:5, R_f_ = 0.370); ^1^H NMR (300 MHz, DMSO-d6) δ: 10.07 (1H, s, NH), 8.59 (2H, d, *J* = 23.2 Hz, Ar), 8.13–7.82 (3H, m, Ar), 7.58 (6H, q, *J* = 8.2 Hz, Ar), 7.21 (2H, d, *J* = 8.0 Hz, Ar), 2.33 (3H, s, Me); ^13^C NMR (75 MHz, DMSO-d6) δ: 155.8, 149.3, 136.1, 134.3, 133.9, 131.3, 129.5, 129.0, 128.9, 128.6, 127.9, 127.3, 126.7, 126.2, 126.1, 125.0, 123.5, 21.0; Anal. calcd for: C, 74.35; H, 4.82; N, 11.82. Found: C, 74.85; H, 4.62; N, 11.09.

#### N-(4-Chlorophenyl)-5-(naphthalen-1-yl)-3-nitropyridin-2-amine (3p)

Red powder, Yield: 89%, m.p: 179–181 °C; (TLC; hexane–EtOAc, 5:1, R_f_ = 0.368); ^1^H NMR (300 MHz, DMSO-d6) δ: 10.12 (1H, s, NH), 8.68 (1H, d, *J* = 2.2 Hz, Ar), 8.59 (1H, d, *J* = 2.3 Hz, Ar), 8.05 (2H, dd, *J* = 7.6, 4.8 Hz, Ar), 7.94–7.84 (1H, m, Ar), 7.84–7.76 (2H, m, Ar), 7.69–7.54 (4H, m, Ar), 7.47 (2H, d, *J* = 8.8 Hz, Ar); ^13^C NMR (75 MHz, DMSO-d6) δ: 155.4, 148.8, 137.9, 136.2, 134.2, 133.9, 131.3, 129.3, 129.0, 128.9, 128.4, 128.0, 127.4, 126.9, 126.7, 126.1, 125.1, 125.0; Anal. calcd for: C, 67.12; H, 3.75; N, 11.18. Found: C, 67.61; H, 3.45; N, 11.52.

#### N,5-Di(naphthalen-1-yl)-3-nitropyridin-2-amine (3q)

Red powder, Yield: 80%, m.p: 162–164; (TLC; hexane–EtOAc, 5:1, R_f_ = 0.372); ^1^H NMR (300 MHz, DMSO-d6) δ; 10.40 (s, 1H), 8.63 (d, *J* = 2.3 Hz, 1H), 8.52 (d, *J* = 2.2 Hz, 1H), 8.11–7.99 (m, 4H), 7.95–7.81 (m, 3H), 7.67–7.51 (m, 8H); Anal. calcd for: C, 62.68; H, 3.71; N, 12.90. Found: C, 62.45; H, 3.56; N, 13.02.

### AChE and BChE inhibition

Cholinesterase inhibitory activities of all analogs were evaluated spectrometrically using the modified Ellman method as previously reported. 20 µl of AChE 0.18 units/ml, or 20 µl BChE iodide 0.162 units/ml and 20 µL DTNB (301 μM) were added to 160 μl sodium phosphate buffer (0.1 mol/l, pH 7.4) in separate wells of a 96-well microplate and gently mixed. Then, 10 μl of different concentrations of test compounds were added to each well and incubated for 15 min at 37 °C followed by the addition of acetylthiocholine (ATCh) or butyrylthiocholine (BTCh) (20 μl, final concentration of 452 μM). The absorbance of each well was measured at 415 nm using a microplate reader. IC_50_ and inhibition values were calculated with the software GraphPad Prism as the mean of three independent experiments and expressed as mean ± SEM^42,43^.

### Enzyme kinetic studies

The mode of inhibition of the most active compound **3m** identified with the lowest IC_50_, was investigated against AChE and BChE in different concentrations of acetylthiocholine or butylthiocholine substrate (0.1–1 mM) as substrate. A Lineweaver–Burk plot was generated to identify the type of inhibition and the Michaelis–Menten constant (Km) value was determined from plot between reciprocal of the substrate concentration (1/[S]) and reciprocal of enzyme rate (1/V) over the various inhibitor concentrations^44,45^.

### Molecular docking

The molecular docking approach was performed using the Schrodinger package’s induced-fit molecular docking (IFD). The SMILE format was converted to a three-dimensional structure within the Maestro software package. The X-ray structures of AChE (PDB code: 4EY7) and BChE (PDB code: 4BDS) were prepared with the Protein Preparation Wizard interface. The molecular docking was performed according to the previpusly reported protocol^46,47^.

### Molecular dynamics simulation

Docked complexes of AChE and BChE complexed with **3m** and apo enzymes were used for the MD simulation according to a previous study using the Schoringer package^48,49^.

### Prime MM-GBSA

The ligand-binding energies (ΔG_Bind_) were calculated using molecular mechanics/generalized born surface area (MM‑GBSA) modules (Schrödinger LLC 2018) based on the following equation

$${\Delta G}_{Bind}={E}_{Complex}-\left({E}_{Receptor}+{E}_{Ligand}\right),$$

where ΔG_Bind_ is the calculated relative free energy in which it includes both receptor and ligand strain energy. E_Complex_ is defined as the MM-GBSA energy of the minimized complex, and E_Ligand_ is the MM-GBSA energy of the ligand after removing it from the complex and allowing it to relax. E_Receptor_ is the MM-GBSA energy of relaxed protein after separating it from the ligand.

### Prediction of pharmacokinetic properties of synthesis compounds

The physicochemical and biological absorption, distribution, metabolism, excretion, and toxicity (ADMET) properties of the selected compound were performed using the ADMETlab 2.0 server (https://admetmesh.scbdd.com/) (accessed on: 12-9-2023).

### Supplementary Information

Supplementary Figures.

## Data Availability

The datasets generated and/or analyzed during the current study are available in the Worldwide Protein Data Bank (wwPDB) repository; with PDB 10.2210/pdb4EY7/pdb and with PDB 10.2210/pdb4BDS/pdb.
