# Supplementary material for: Synthesis of novel aryl-substituted 2-aminopyridine derivatives by the cascade reaction of 1,1-enediamines with vinamidinium salts to develop novel anti-Alzheimer agents
Source: Sci Rep. 2024 Jun 14;14:13780. doi: 10.1038/s41598-024-64179-1 (PMC11178820; doi:10.1038/s41598-024-64179-1)
Supplement: Supplementary file 1 — Supplementary Figures. [file 41598_2024_64179_MOESM1_ESM.pdf]

Fig S1. N-(4-methoxyphenyl)-3-nitro-5-phenylpyridin-2-amine (**3a**)

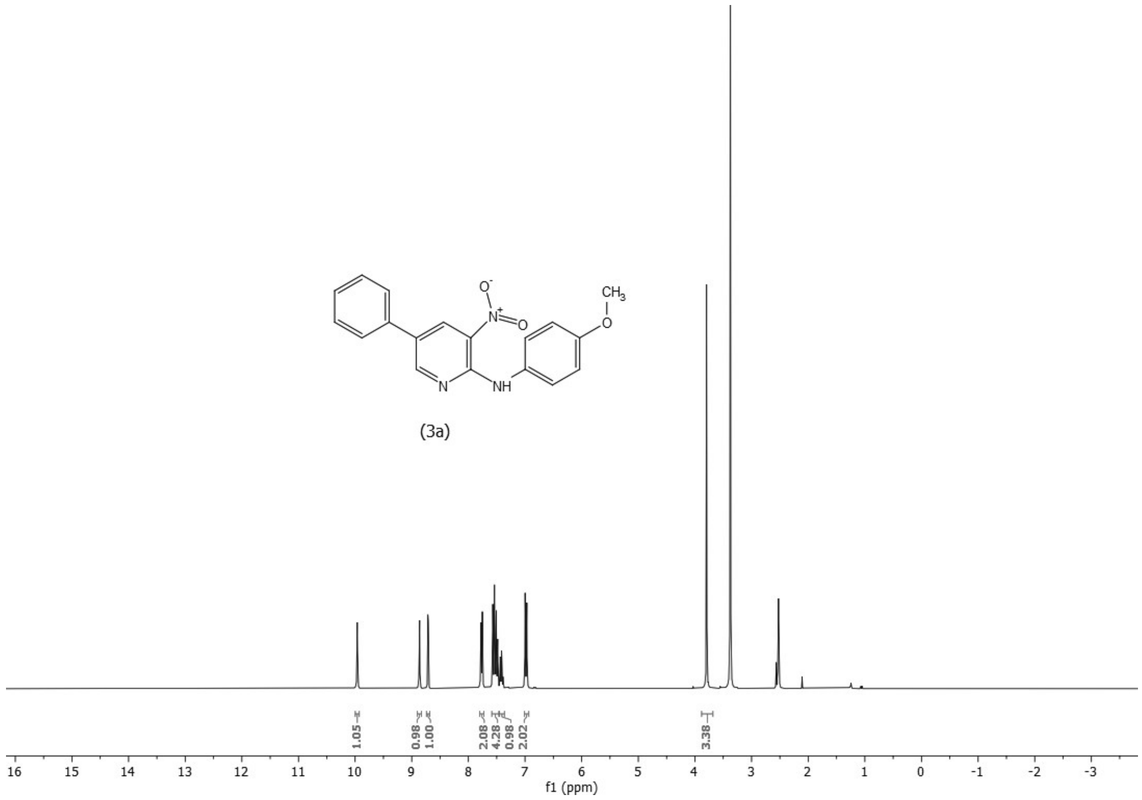

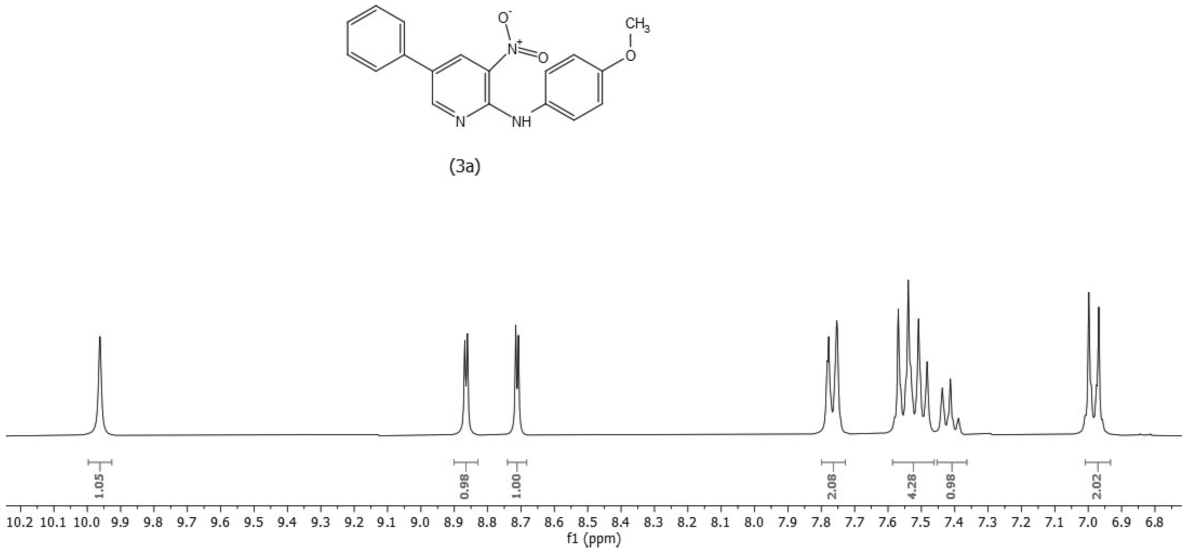

Fig S1. N-(4-methoxyphenyl)-3-nitro-5-phenylpyridin-2-amine (**3a**)

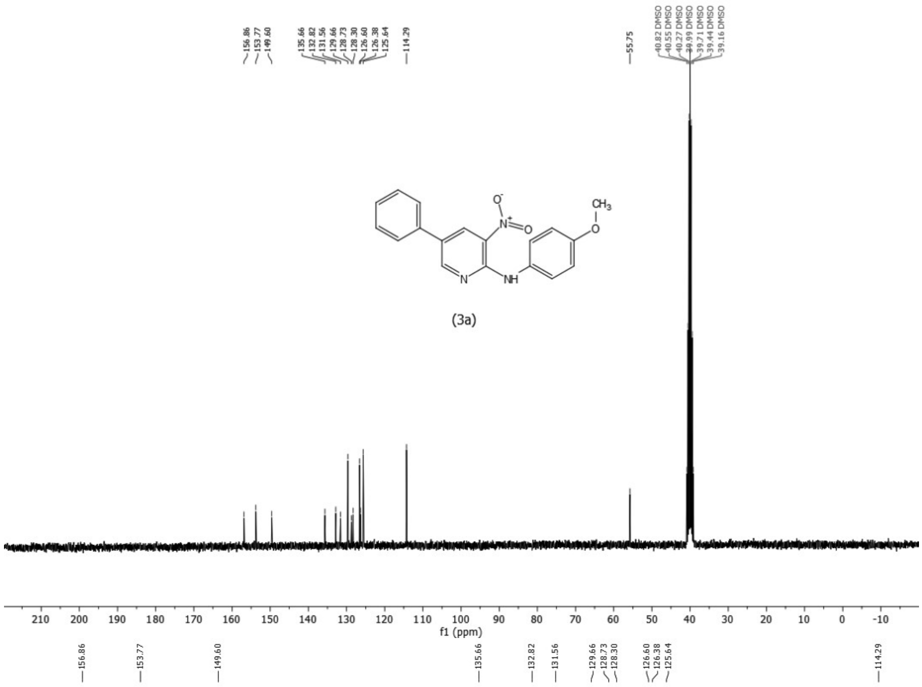

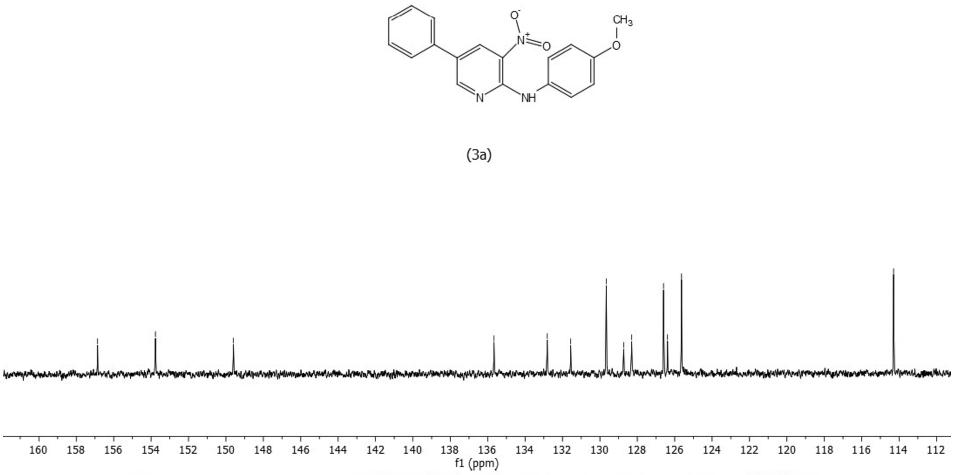

Chromatogram C:\EAS Clarity\Work1\DATA\k3 1402.5.16_1_3_2002 5_26_03 AM_089.PRM
PRM *Result Table (ESTD – k3 1402.5.16_1_3_2002 5_26_03 AM_089 - INT7 - 1)*

	Reten. Time [min]	Response	Weight [mg]	Weight [%]	Peak Type	Element Name	Carbon Response Ratio
1	1.452	1151.214	0.402	10.33	Refer	Nitrogen	0.113
3	3.253	3125.889	0.704	53.94	Refer	Carbon	1.000
4	11.458	867.698	0.089	3.64	Refer	Hydrogen	0.168
	Total		1.195	67.91			

CHN for $C_{18}H_{15}N_3O_3$ (3a)

Fig S2. N-(4-chlorophenyl)-3-nitro-5-phenylpyridin-2-amine (**3b**)

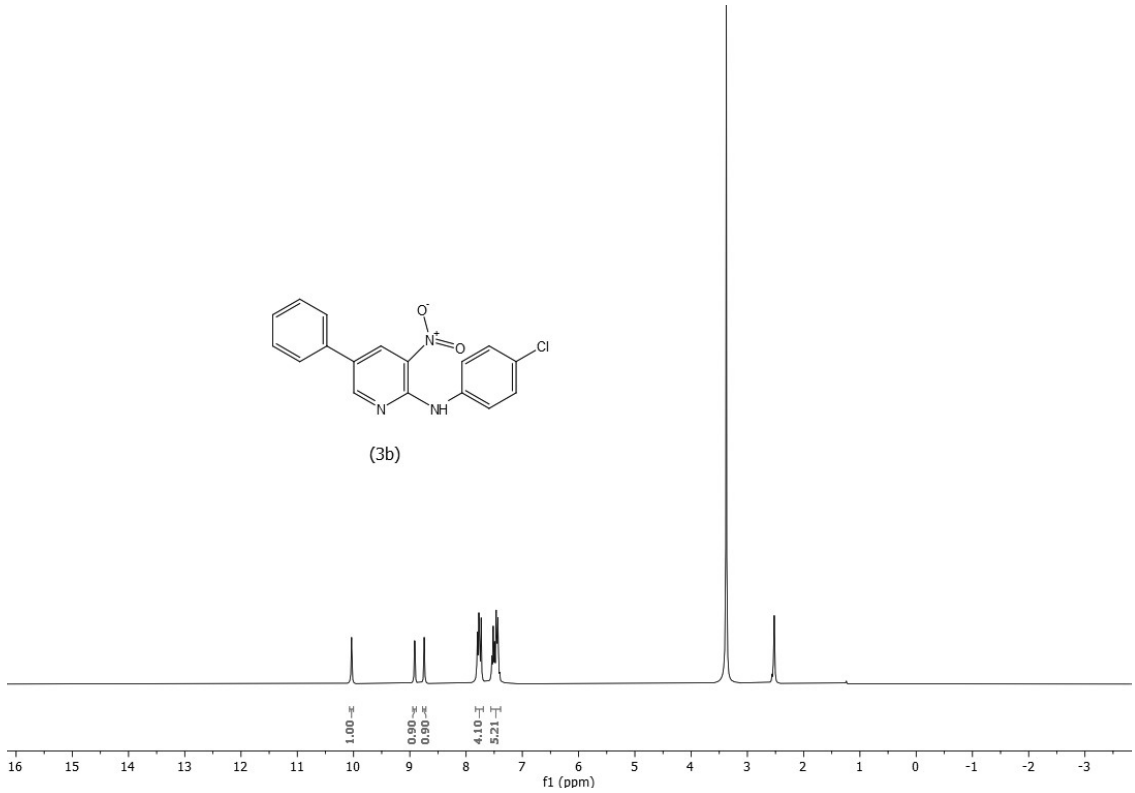

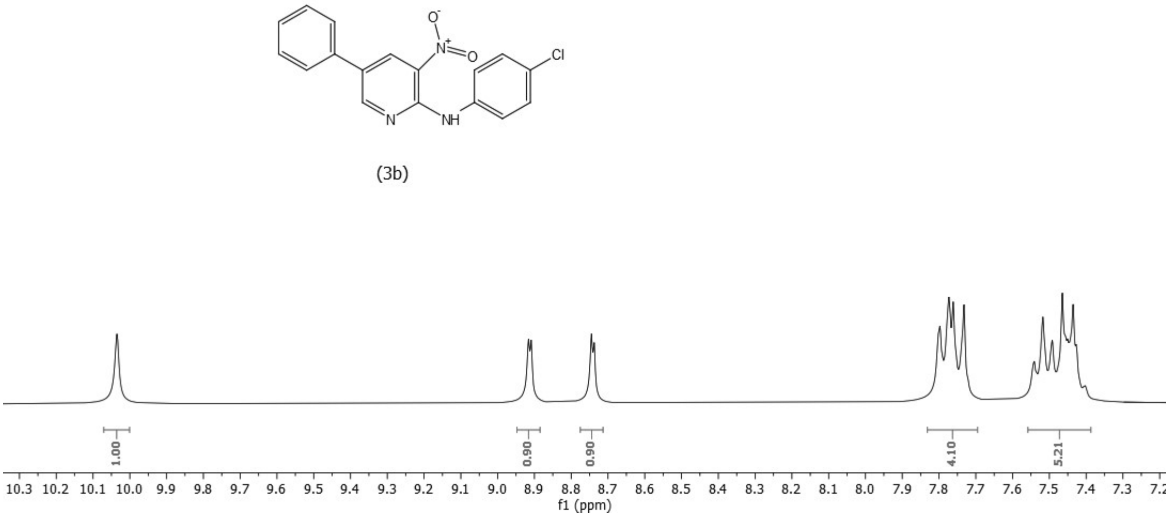

Fig S2. N-(4-chlorophenyl)-3-nitro-5-phenylpyridin-2-amine (**3b**)

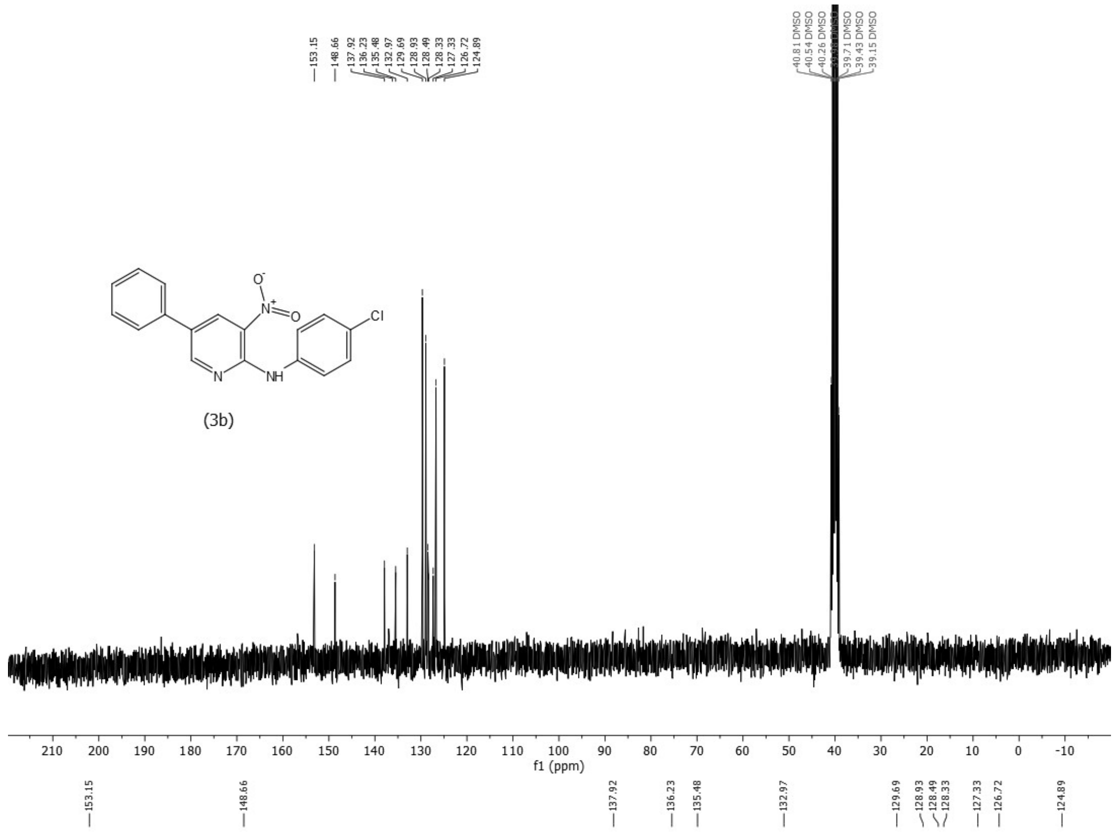

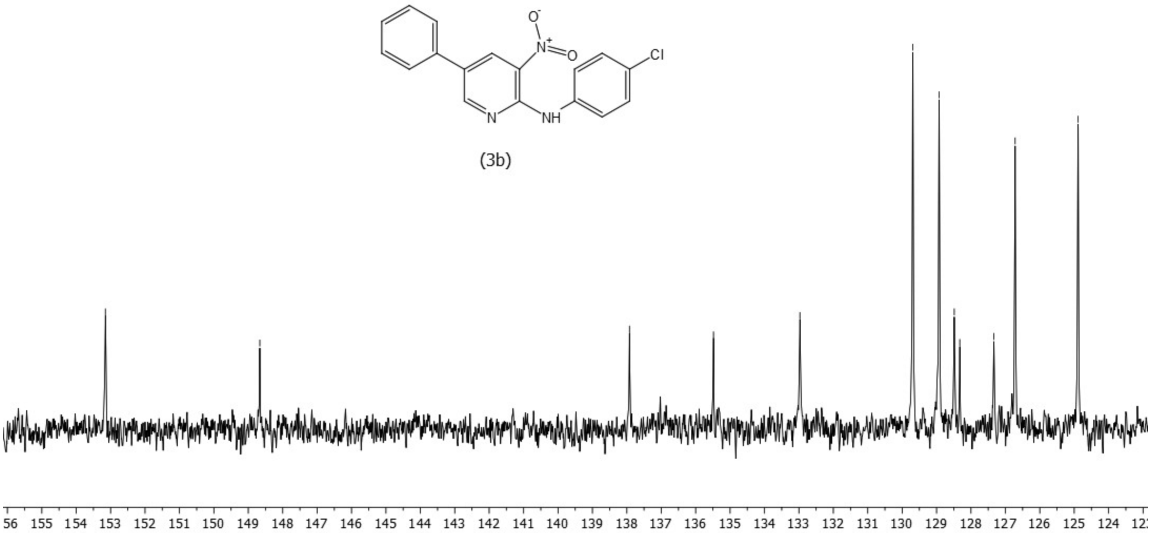

Fig S2. N-(4-chlorophenyl)-3-nitro-5-phenylpyridin-2-amine (**3b**)

Chromatogram C:\EAS Clarity\WORK1\Data\k4 1402.5.16_1_3_2002 1_52_02 AM_069.PRM
Result Table (ESTD - k4 1402.5.16_1_3_2002 1_52_02 AM_069 - INT7 - 1)

	Reten. Time [min]	Response	Weight [mg]	Weight [%]	Peak Type	Element Name	Carbon Response Ratio
1	1.303	597.118	0.402	11.49	Refer	Nitrogen	0.085
3	2.460	7053.697	0.704	60.89	Refer	Carbon	1.000
4	10.557	1198.279	0.089	4.05	Refer	Hydrogen	0.170
	Total		1.195	67.91			

CHN for $C_{17}H_{12}ClN_3O_2$ (3b)

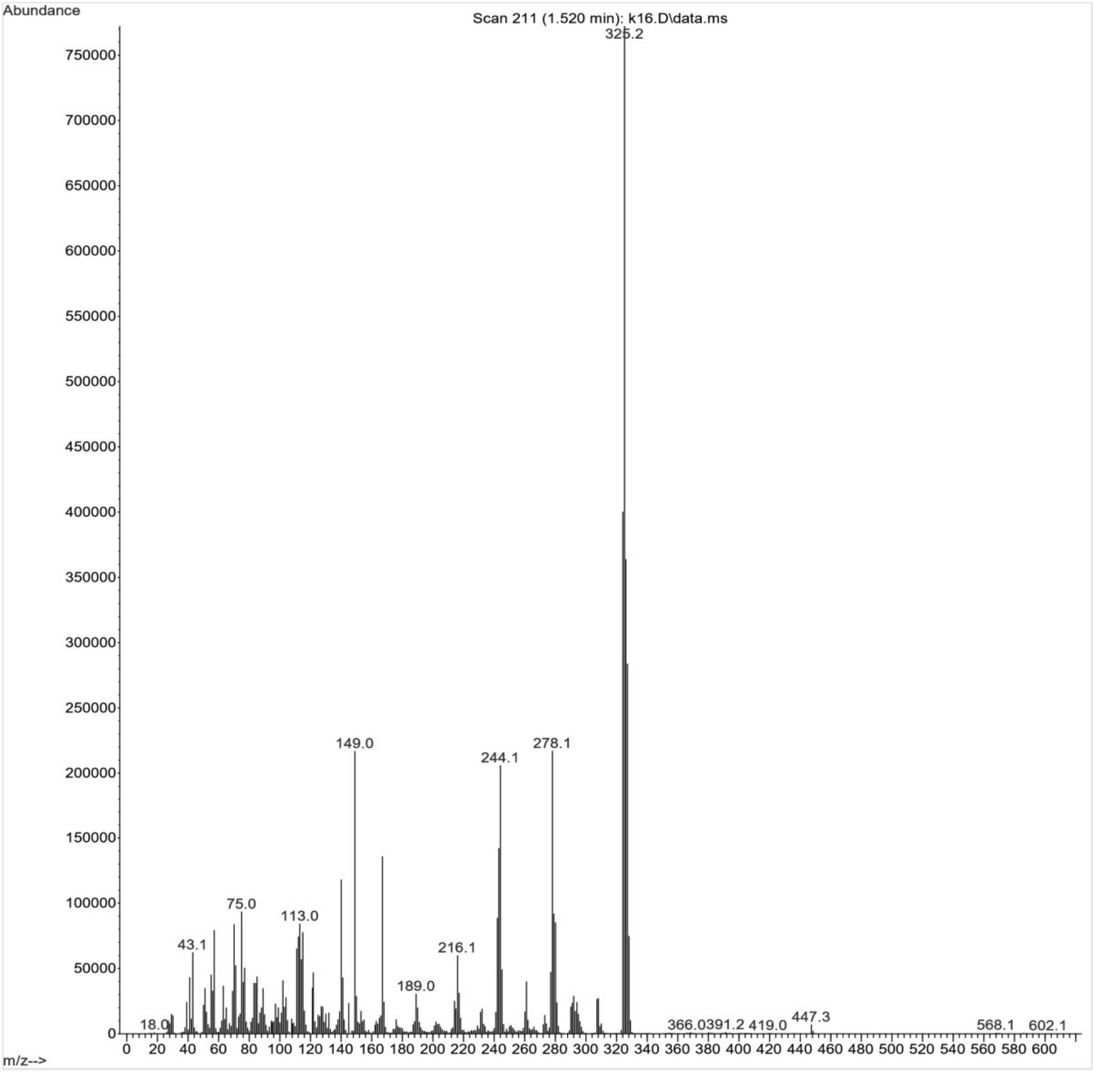

$C_{17}H_{12}ClN_3O_2$ (3b)

MS (*m/z*): 325

Fig S3. 5-(4-fluorophenyl)-N-(4-methoxyphenyl)-3-nitropyridin-2-amine (**3c**)

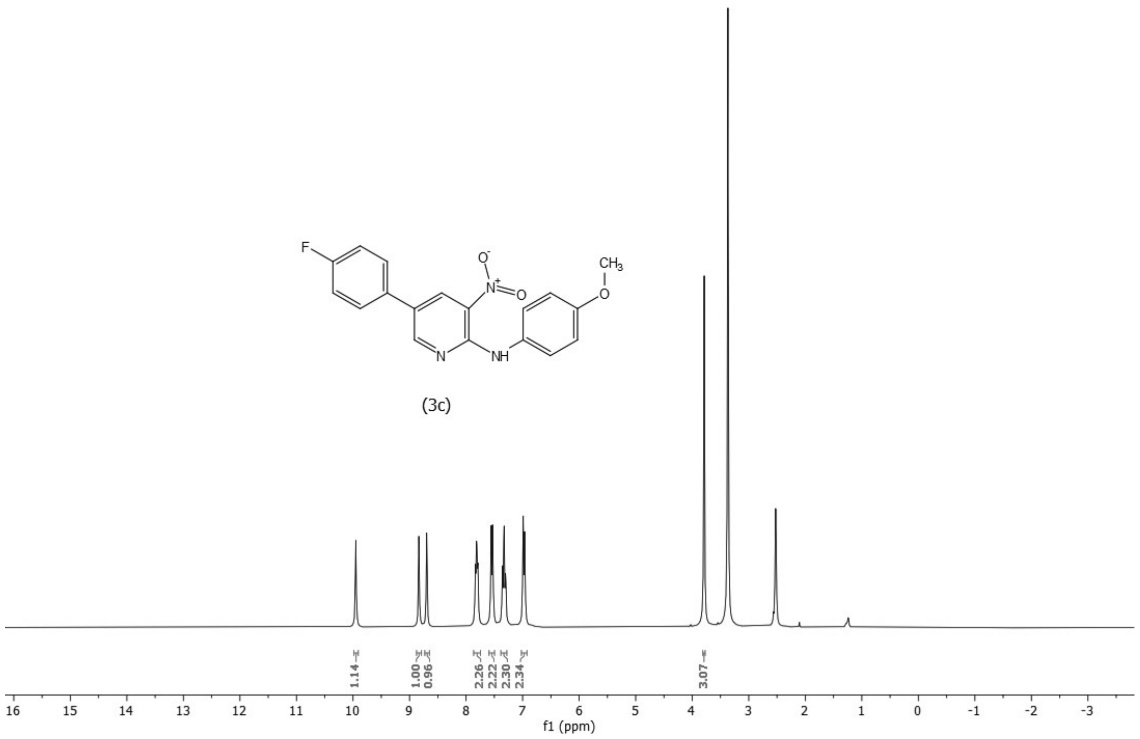

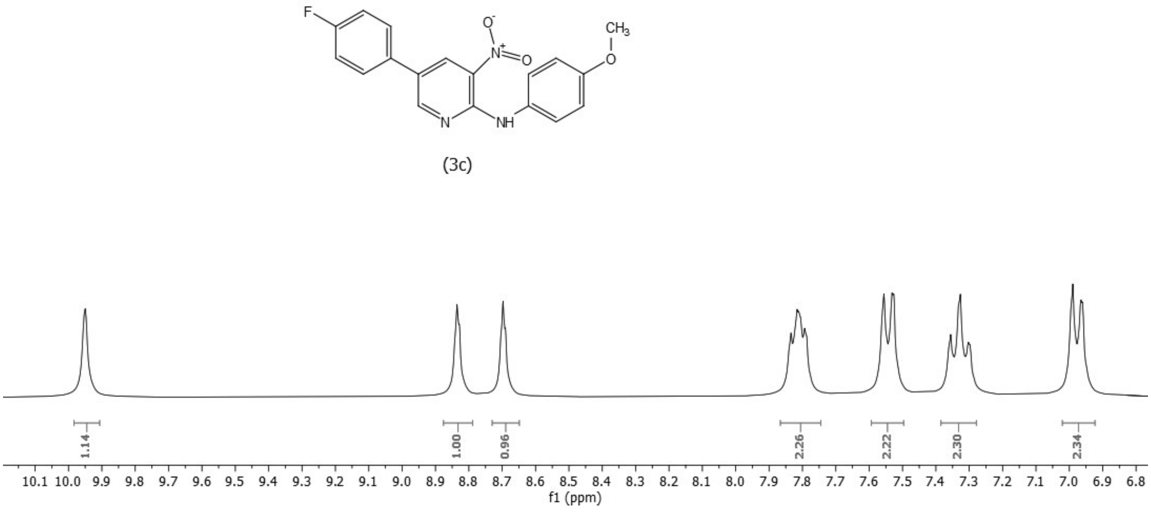

Fig S3. 5-(4-fluorophenyl)-N-(4-methoxyphenyl)-3-nitropyridin-2-amine (**3c**)

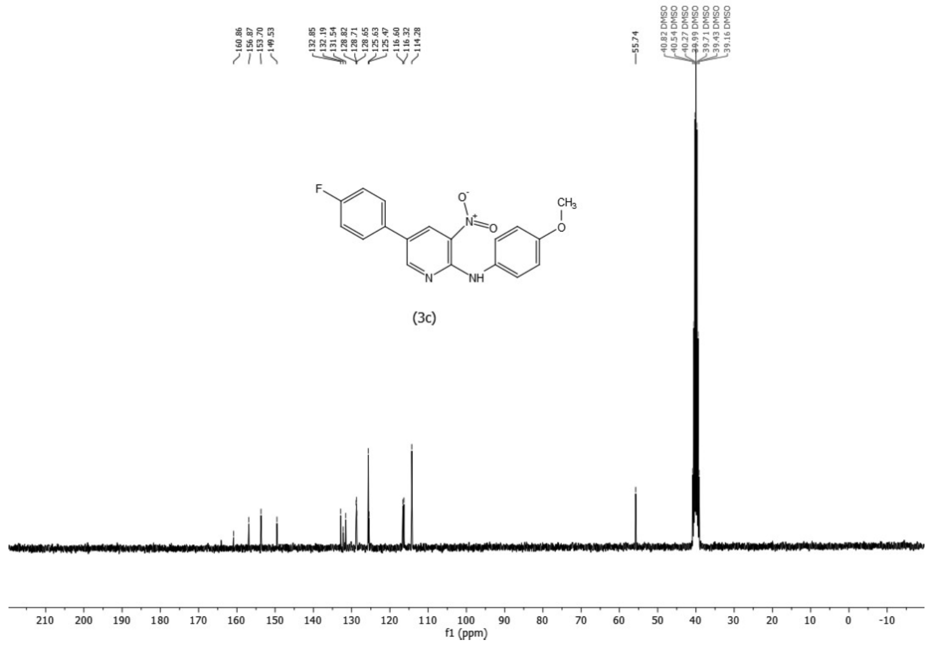

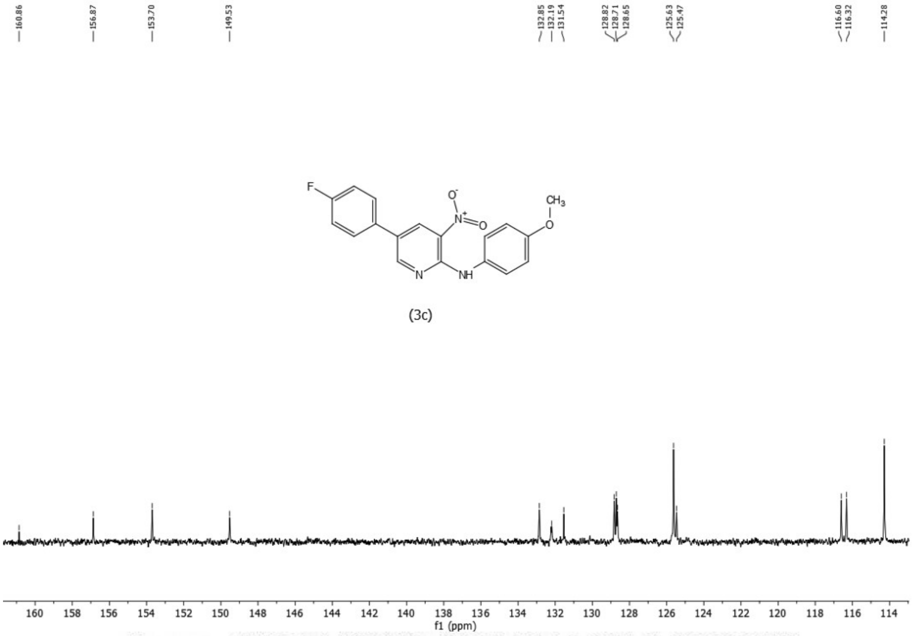

Chromatogram C:\EAS Clarity\WORK1\Data\k7 1402.5.15_1_2_2002 4_24_00 AM_062.PRM
Result Table (ESTD – k7 1402.5.15_1_2_2002 4_24_00 AM_062 - INT7 - 1)

	Reten. Time [min]	Response	Weight [mg]	Weight [%]	Peak Type	Element Name	Carbon Response Ratio
1	1.320	322.499	0.138	12.62	Refer	Nitrogen	0.086
3	2.635	2257.372	0.386	63.41	Refer	Carbon	1.000
4	11.985	448.038	0.023	4.29	Refer	Hydrogen	0.169
	Total		1.118	80.32			

CHN for $C_{18}H_{14}FN_3O_3$ (3c)

Fig S4. 5-(4-fluorophenyl)-3-nitro-N-(p-tolyl)pyridin-2-amine (**3d**)

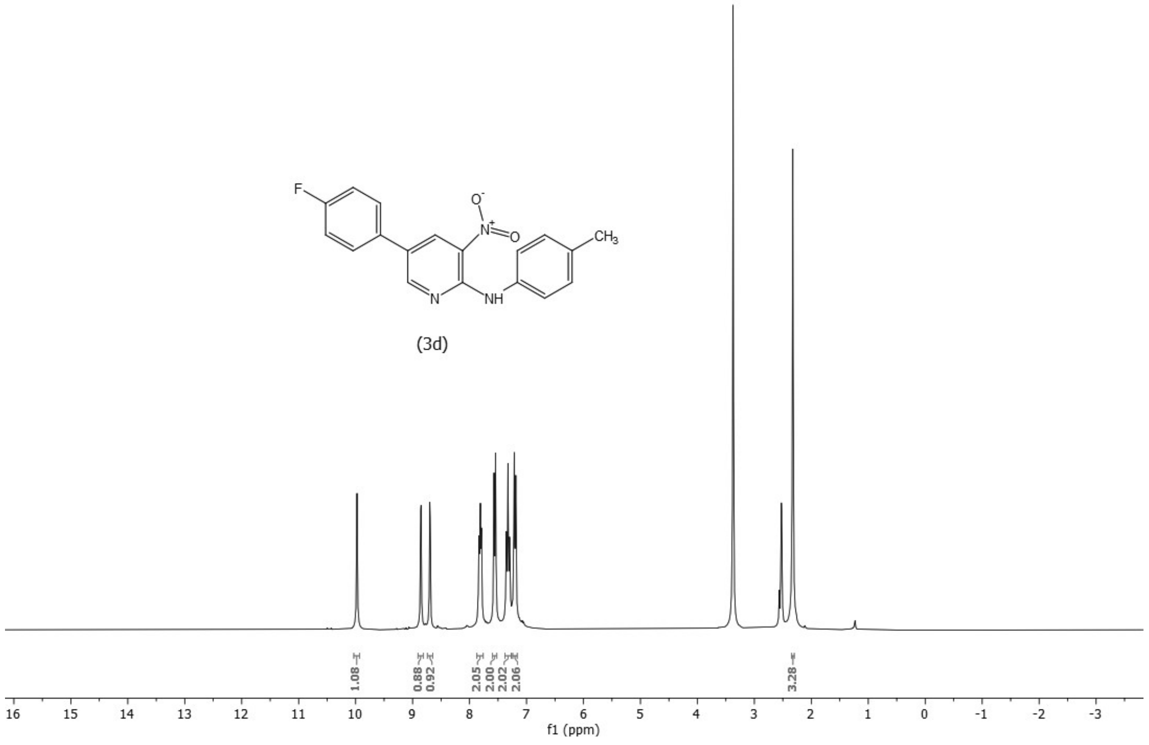

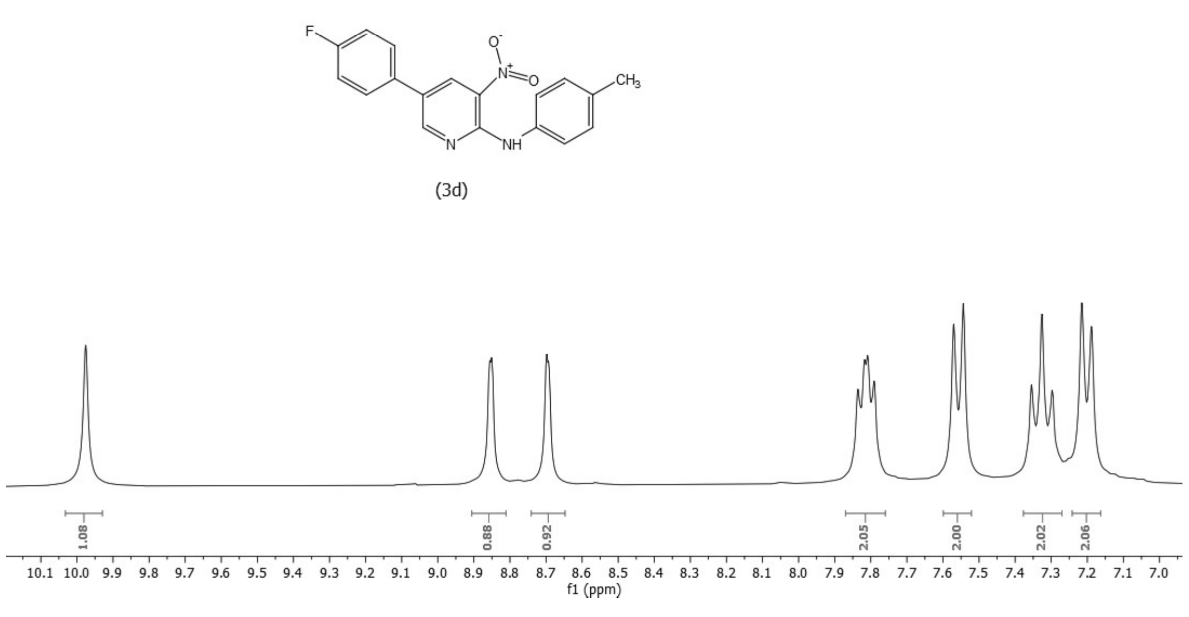

Fig S4. 5-(4-fluorophenyl)-3-nitro-N-(p-tolyl)pyridin-2-amine (**3d**)

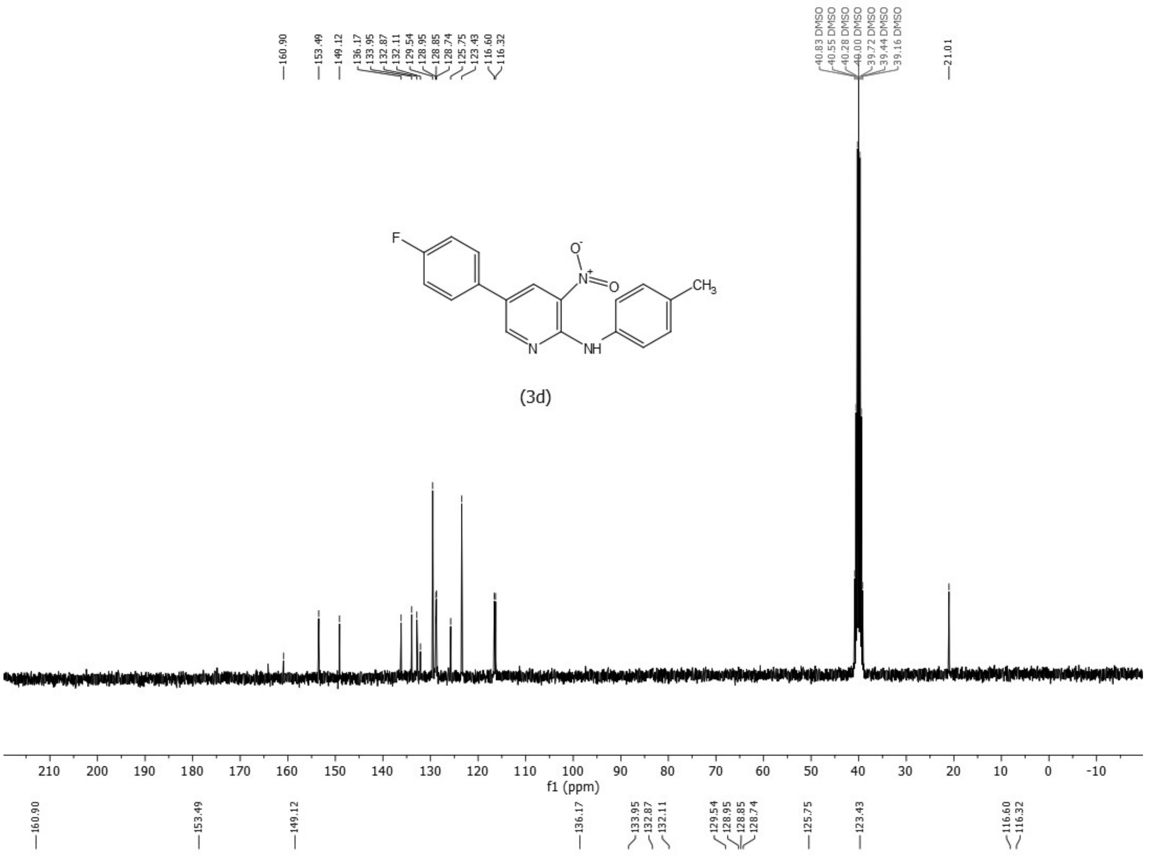

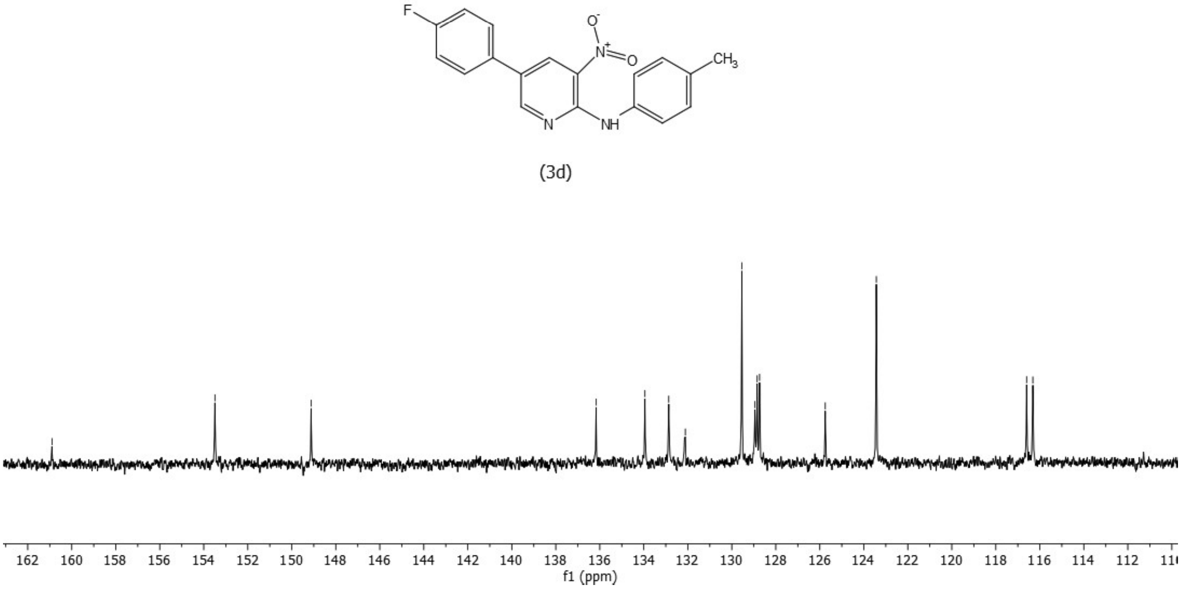

Chromatogram C:\EAS Clarity\WORK1\Data\k8 1402.5.15_1_2_2002 2_24_00 AM_058.PRM
Result Table (ESTD – k8 1402.5.15_1_2_2002 2_24_00 AM_058 - INT7 - 1)

	Reten. Time [min]	Response	Weight [mg]	Weight [%]	Peak Type	Element Name	Carbon Response Ratio
1	1.297	559.797	0.264	11.06	Refer	Nitrogen	0.095
3	2.533	3814.657	0.699	56.96	Refer	Carbon	1.000
4	10.810	907.988	0.054	3.42	Refer	Hydrogen	0.189
	Total		1.370	71.44			

CHN for $C_{18}H_{14}FN_3O_2$ (3d)

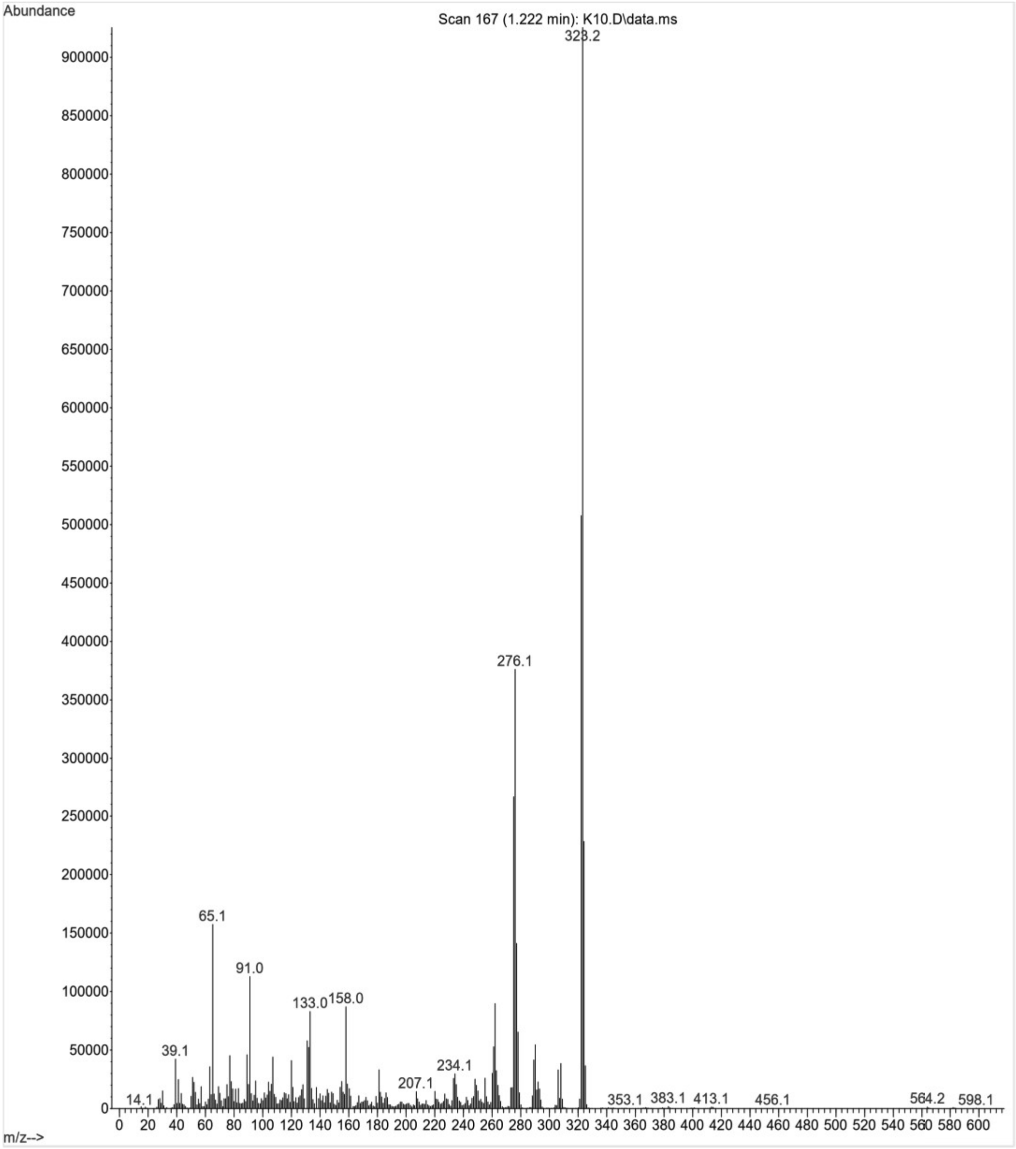

$C_{18}H_{14}FN_3O_2$ (3d)

MS (*m/z*): 323

Fig S5. N-(4-chlorophenyl)-5-(4-fluorophenyl)-3-nitropyridin-2-amine (**3e**)

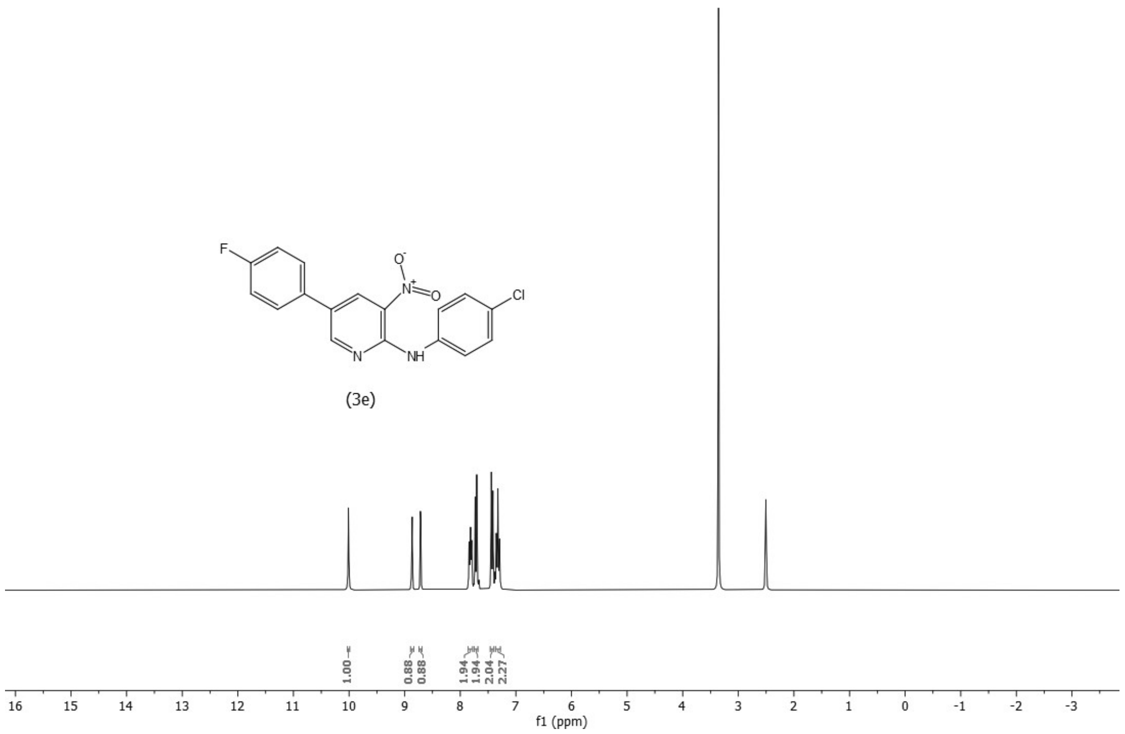

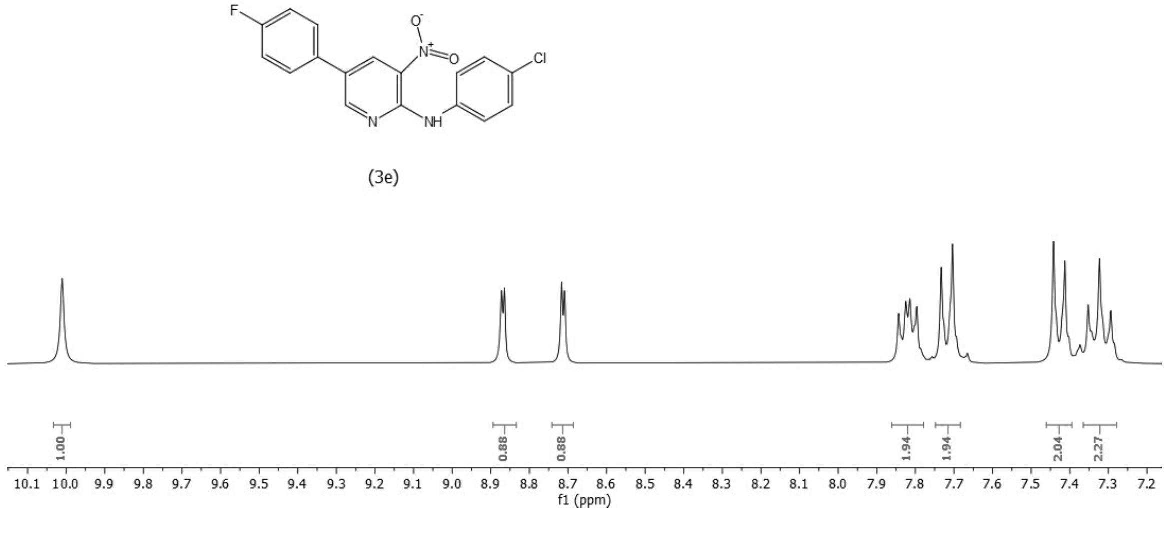

Fig S5. N-(4-chlorophenyl)-5-(4-fluorophenyl)-3-nitropyridin-2-amine (**3e**)

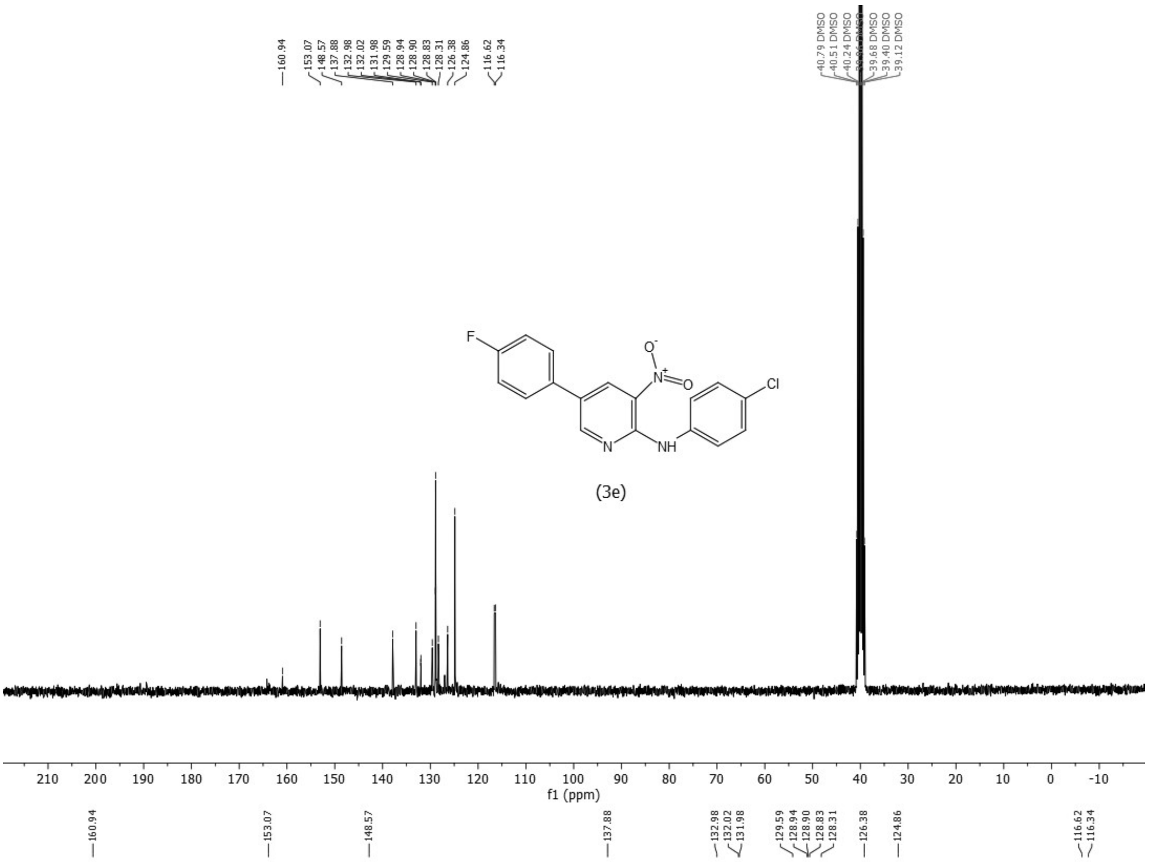

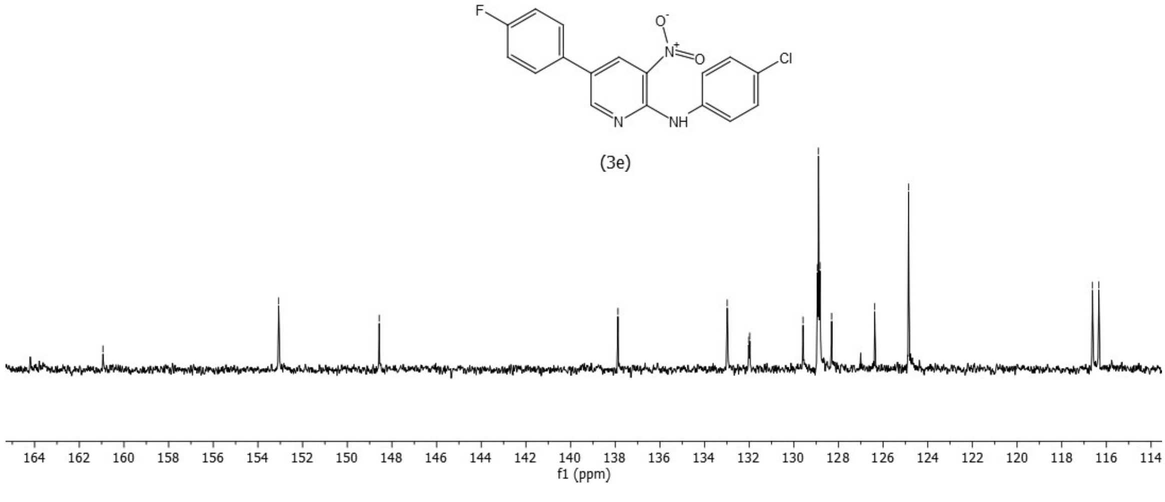

Fig S5. N-(4-chlorophenyl)-5-(4-fluorophenyl)-3-nitropyridin-2-amine (**3e**)

Chromatogram C:\EAS Clarity\Work1\DATA\k10 1402.5.16_1_3_2002 3_22_02 AM_072.PRM
Result Table (ESTD - k10 1402.5.16_1_3_2002 3_22_02 AM_072 - INT7 – 1)

	Reten. Time [min]	Response	Weight [mg]	Weight [%]	Peak Type	Element Name	Carbon Response Ratio
1	1.333	911.317	0.450	12.85	Refer	Nitrogen	0.083
3	2.440	1097.823	2.409	66.58	Refer	Carbon	1.000
4	10.317	1979.911	0.124	4.42	Refer	Hydrogen	0.180
	Total		3.062	83.85			

CHN for $C_{17}H_{11}ClFN_3O_2$ (**3e**)

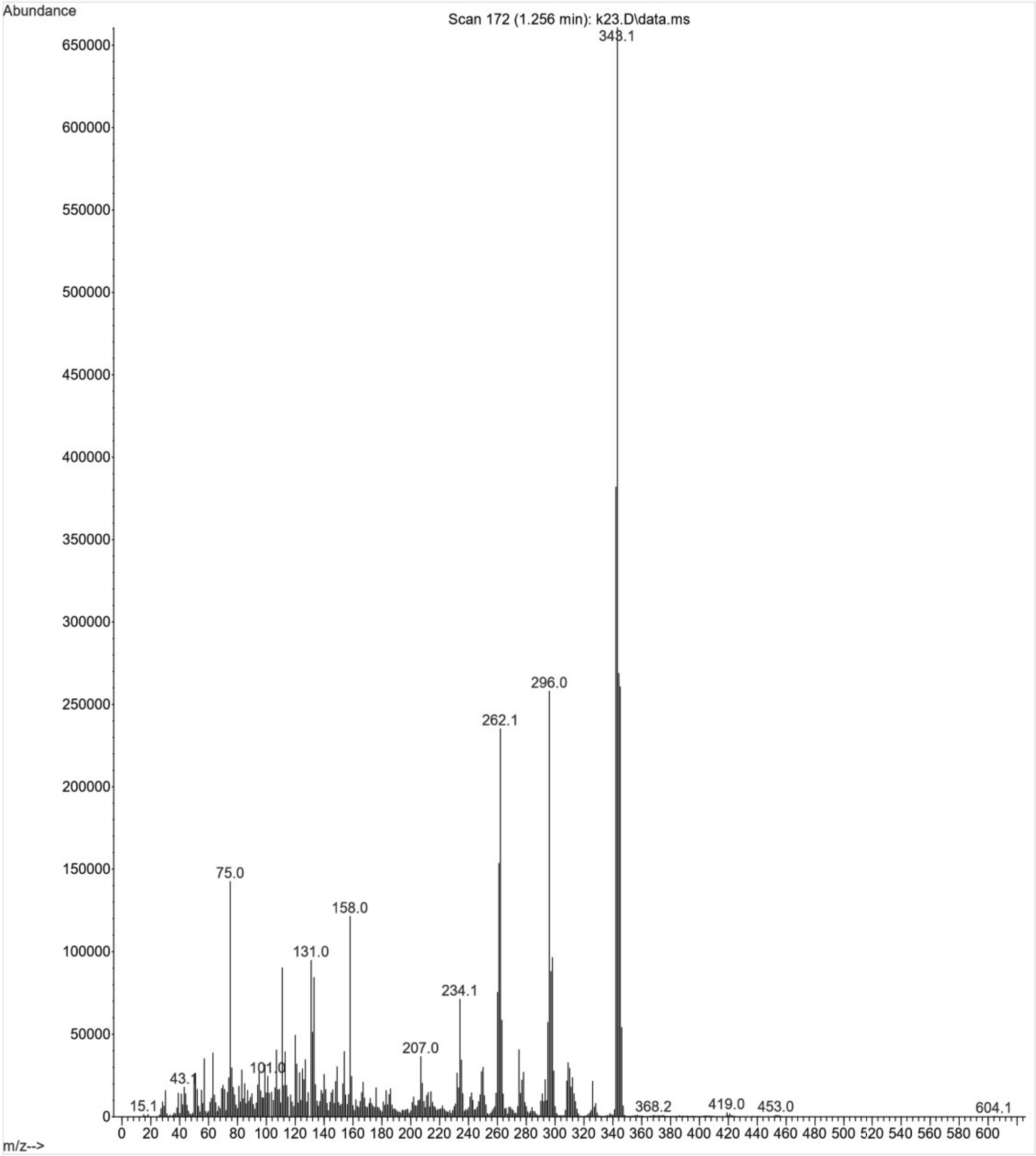

$C_{17}H_{11}ClFN_3O_2$ (**3e**)

MS (*m/z*): 343

Fig S6. 5-(4-chlorophenyl)-N-(4-methoxyphenyl)-3-nitropyridin-2-amine (**3f**)

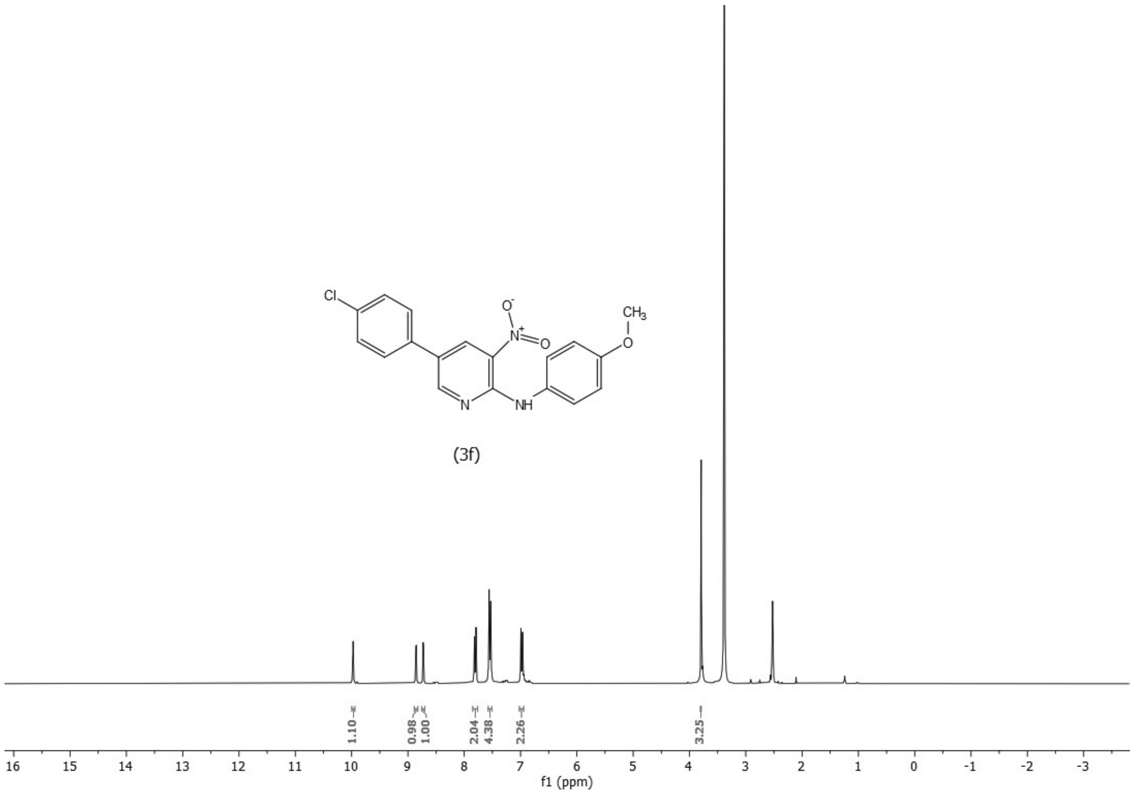

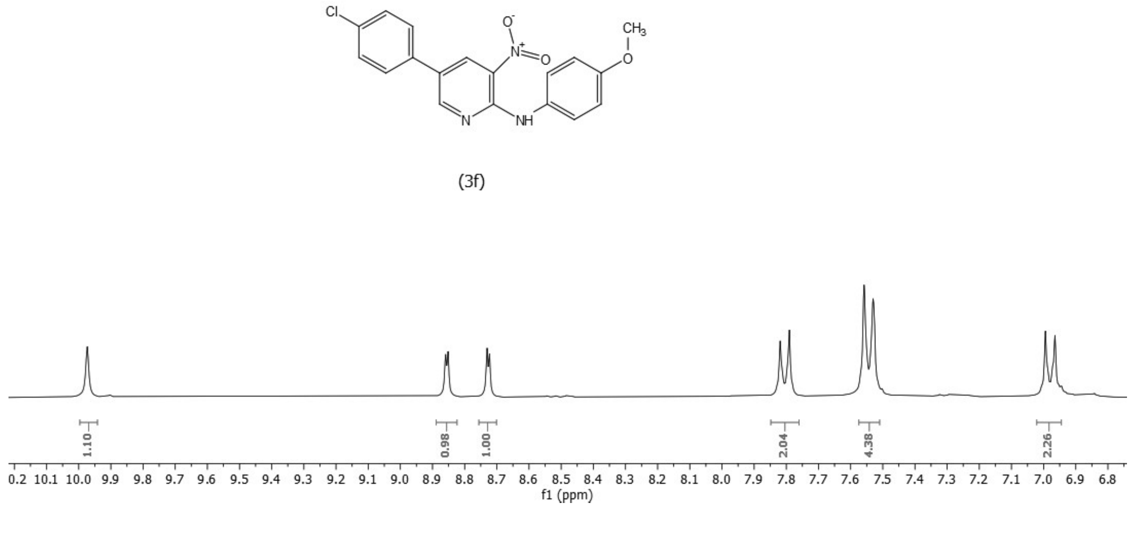

Fig S6. 5-(4-chlorophenyl)-N-(4-methoxyphenyl)-3-nitropyridin-2-amine (**3f**)

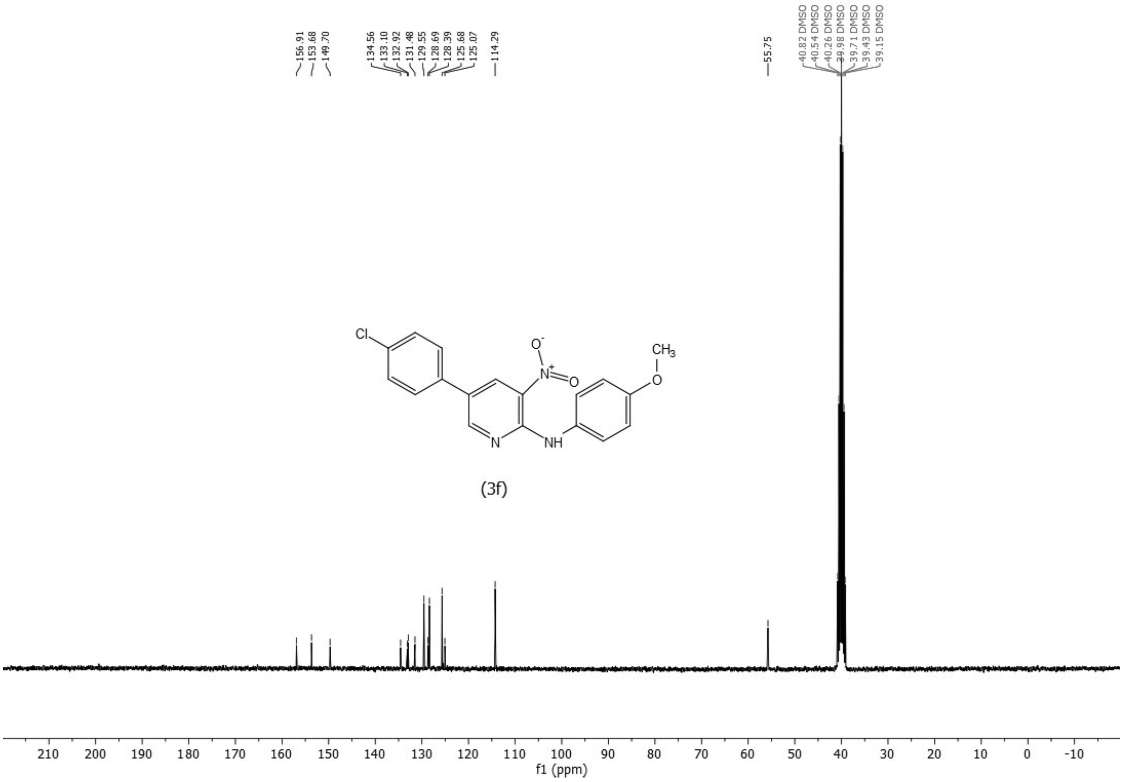

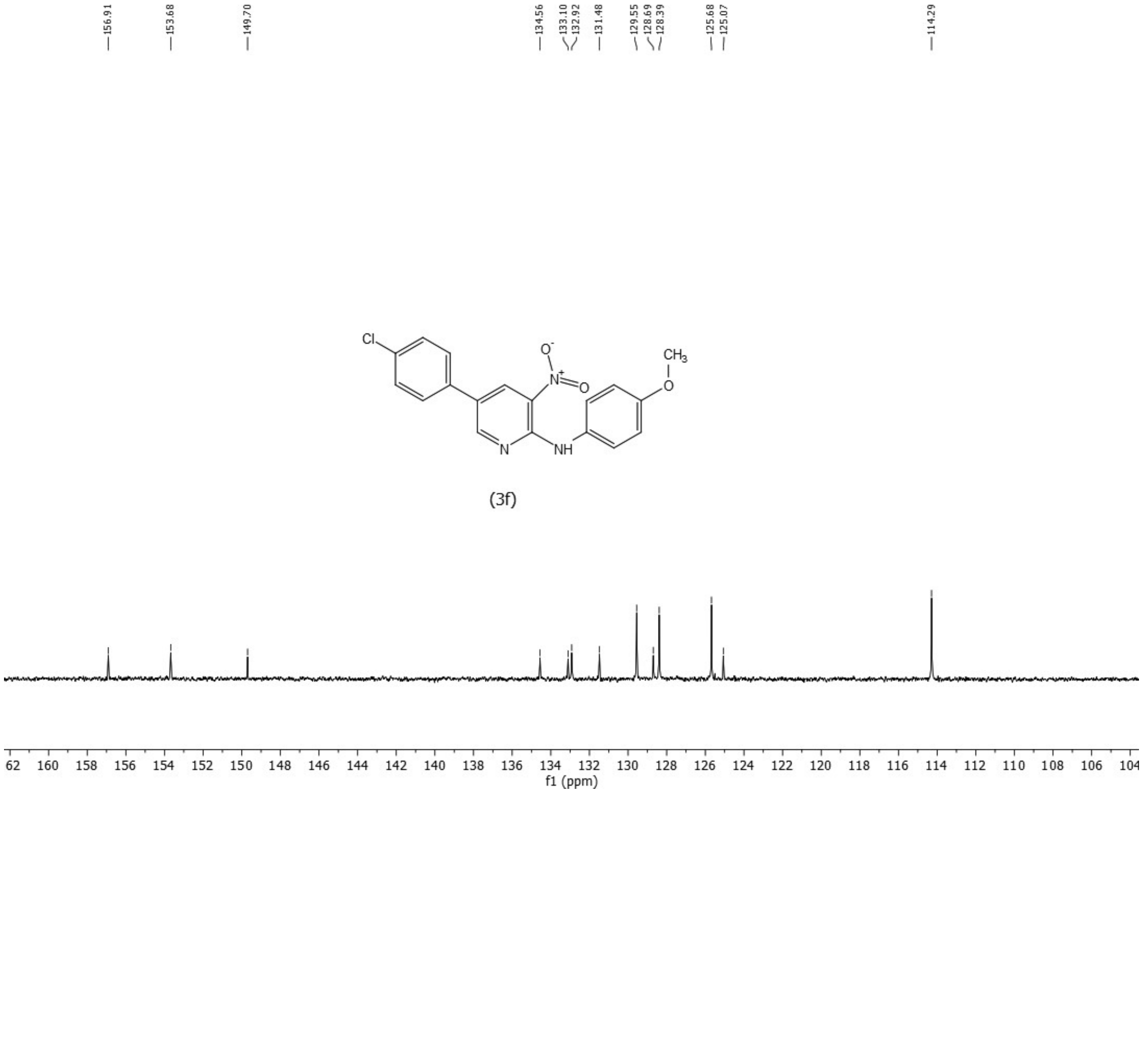

Fig S6. 5-(4-chlorophenyl)-N-(4-methoxyphenyl)-3-nitropyridin-2-amine (**3f**)

Chromatogram C:\EAS Clarity\WORK1\Data\k12 1402.5.16_1_3_2002 1_22_02 AM_068.PRM
Result Table (ESTD - k12 1402.5.16_1_3_2002 1_22_02 AM_068 - INT7 - 1)

	Reten. Time [min]	Response	Weight [mg]	Weight [%]	Peak Type	Element Name	Carbon Response Ratio
1	1.283	369.178	0.163	12.44	Refer	Nitrogen	0.088
3	2.533	4186.738	0.776	63.58	Refer	Carbon	1.000
4	11.417	965.041	0.040	4.29	Refer	Hydrogen	0.166
	Total		1.164	80.31			

CHN for $C_{18}H_{14}ClN_3O_3$ (**3f**)

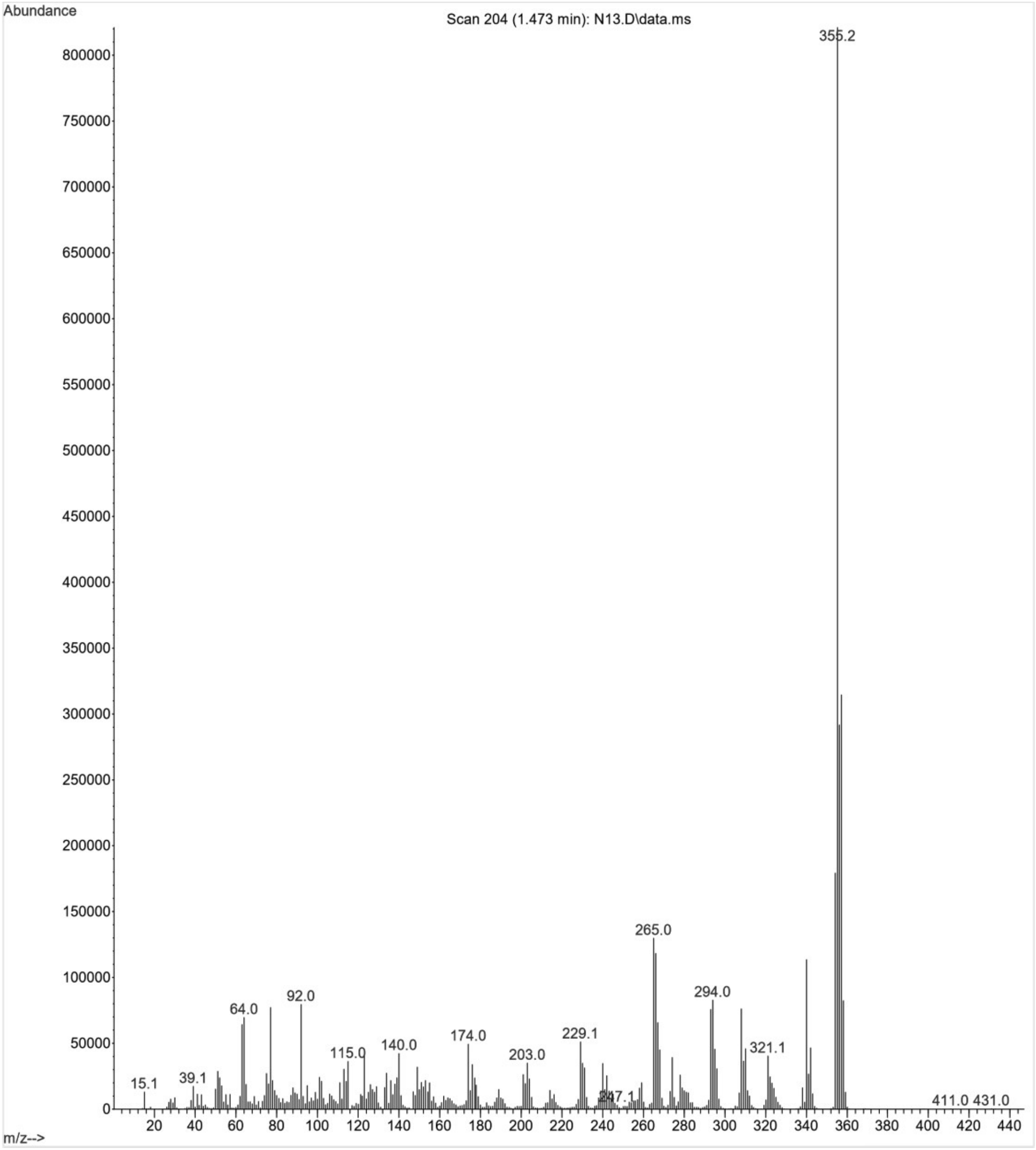

$C_{18}H_{14}ClN_3O_3$ (**3f**)

MS (*m/z*): 355

Fig S7. N,5-bis(4-chlorophenyl)-3-nitropyridin-2-amine (**3g**)

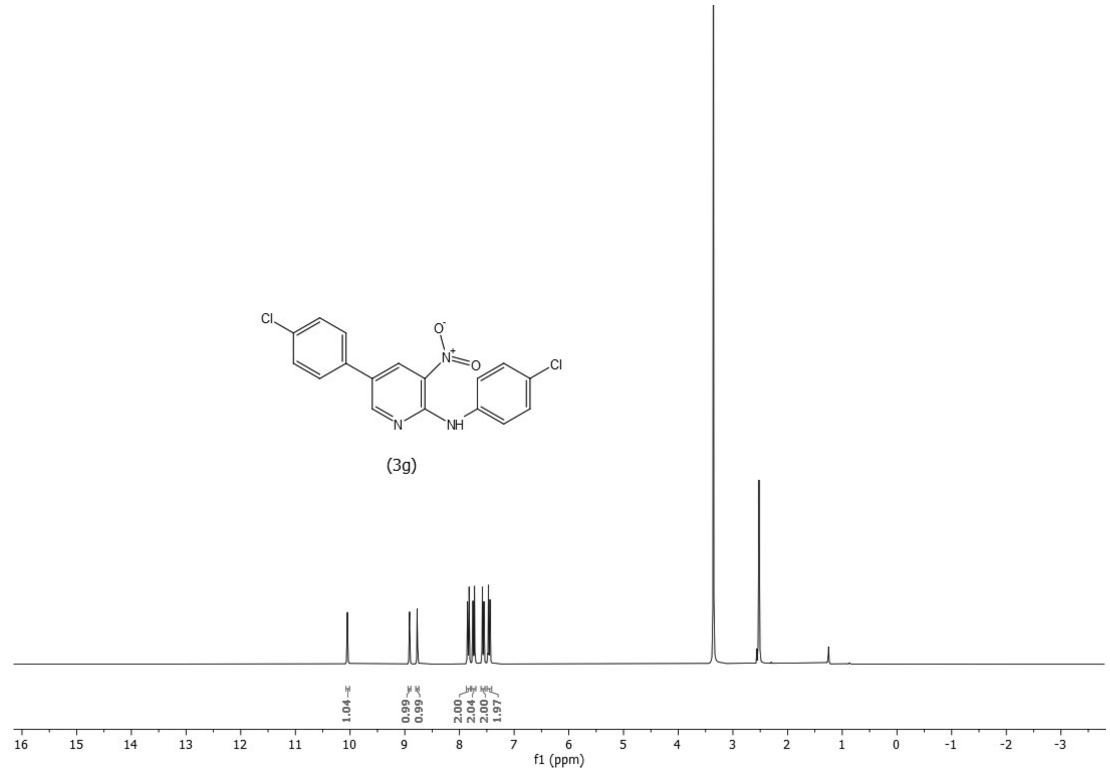

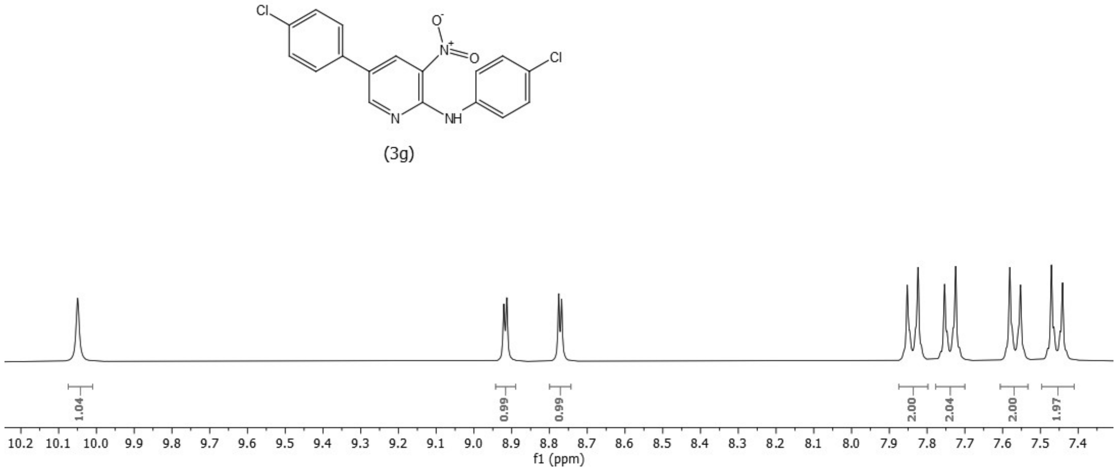

Fig S7. N,5-bis(4-chlorophenyl)-3-nitropyridin-2-amine (**3g**)

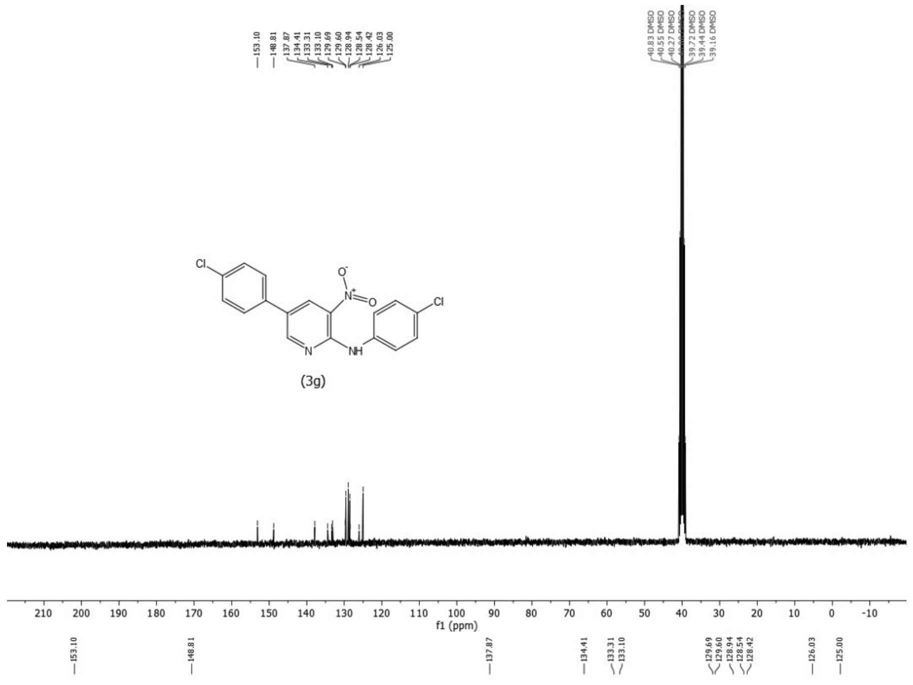

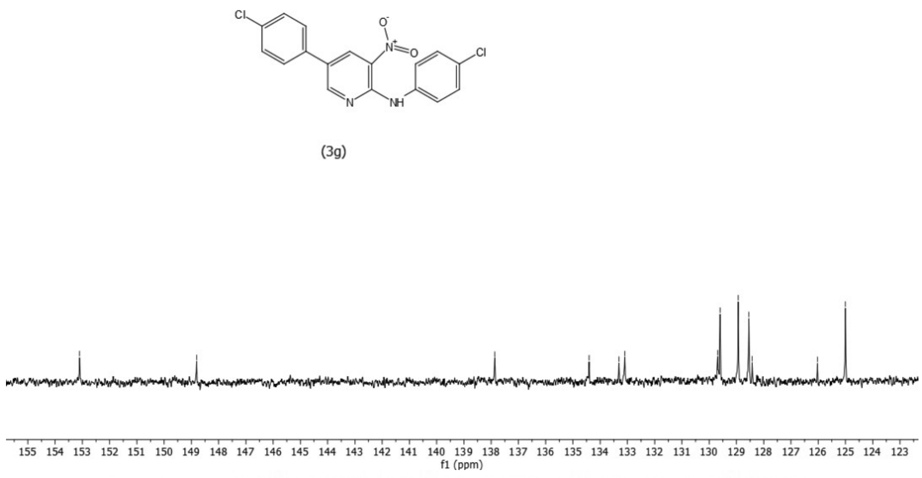

Chromatogram C:\EAS Clarity\Work1\DATA\k14 1402.5.16_1_3_2002 6_22_03 AM_078.PRM
Result Table (ESTD - k14 1402.5.16_1_3_2002 6_22_03 AM_078 - INT7 - 1)

	Reten. Time [min]	Response	Weight [mg]	Weight [%]	Peak Type	Element Name	Carbon Response Ratio
1	1.413	1131.023	0.567	11.63	Refer	Nitrogen	0.083
3	2.537	13320.381	3.135	56.19	Refer	Carbon	1.000
4	10.960	2059.383	0.129	3.13	Refer	Hydrogen	0.158
	Total		4.098	70.95			

CHN for $C_{17}H_{11}Cl_2N_3O_2$ (**3g**)

Fig S8. 5-(4-chlorophenyl)-3-nitro-N-(o-tolyl)pyridin-2-amine (**3h**)

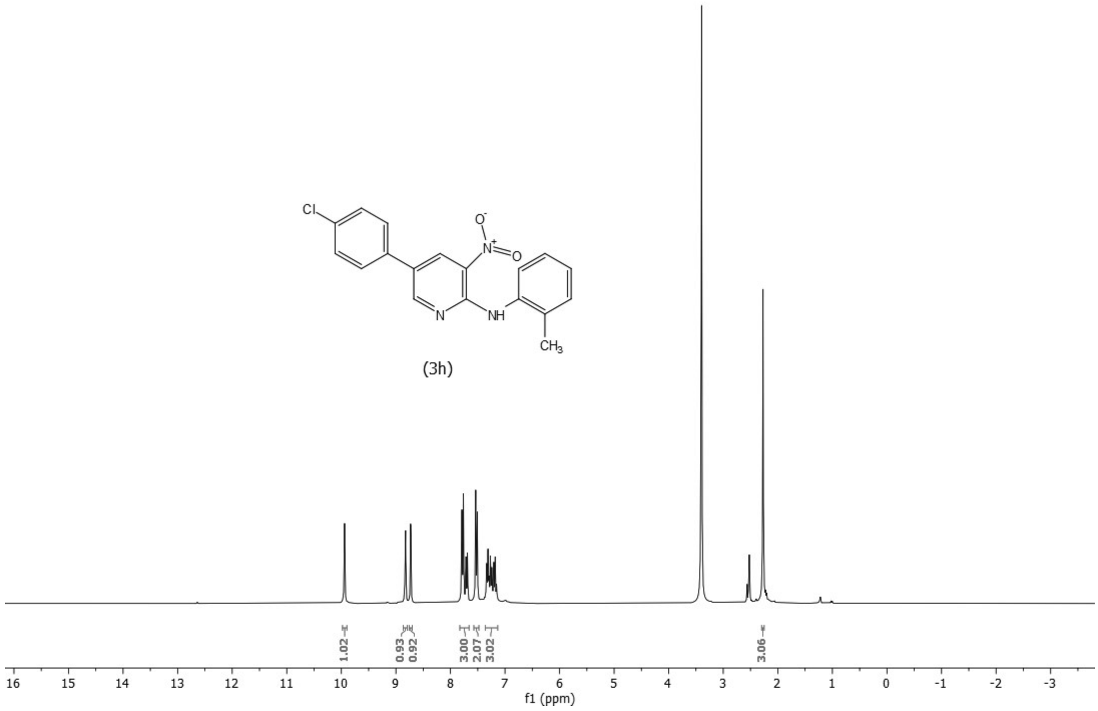

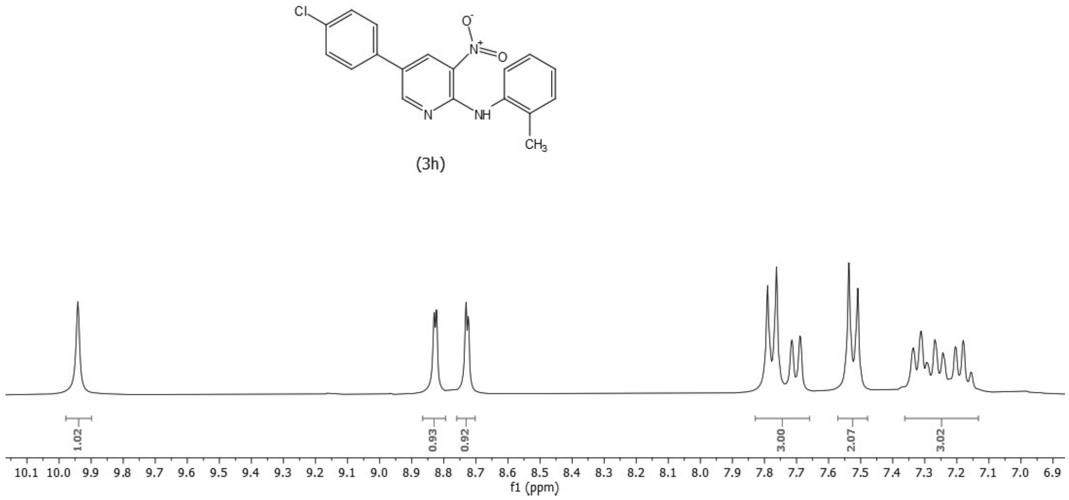

Fig S8. 5-(4-chlorophenyl)-3-nitro-N-(o-tolyl)pyridin-2-amine (**3h**)

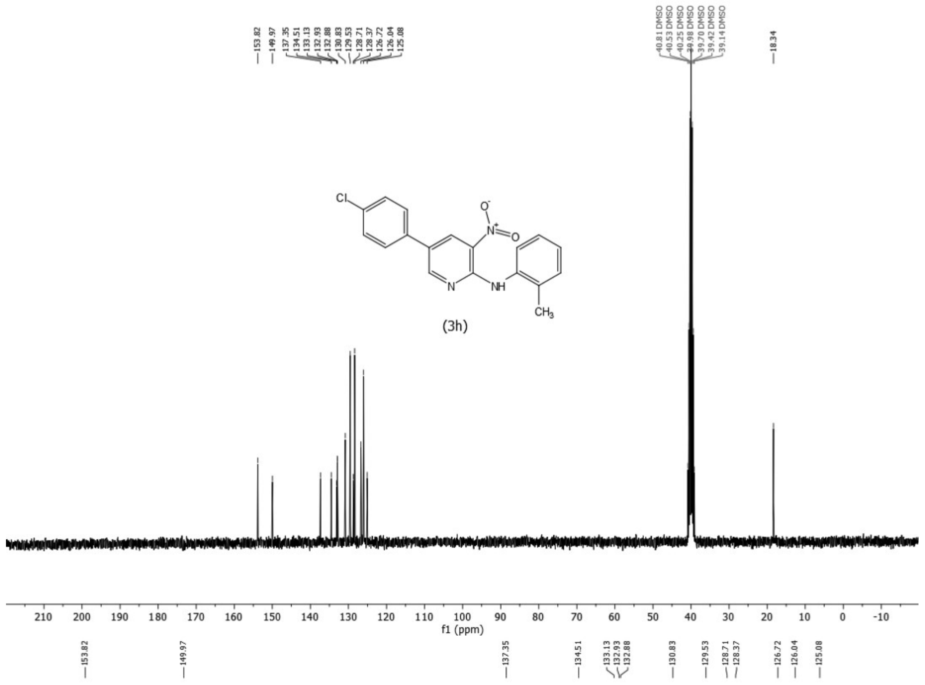

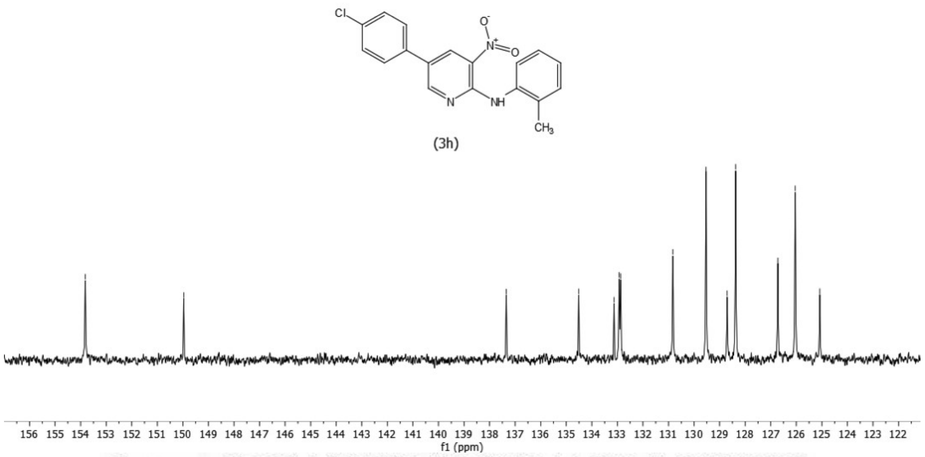

Chromatogram C:\EAS Clarity\WORK1\Data\k15 1402.5.15_1_2_2002 2_54_00 AM_059.PRM
Result Table (ESTD – k15 1402.5.15_1_2_2002 2_54_00 AM_059 - INT7 - 1)

	Reten. Time [min]	Response	Weight [mg]	Weight [%]	Peak Type	Element Name	Carbon Response Ratio
1	1.293	2094.803	0.124	10.49	Refer	Nitrogen	0.079
3	2.607	2279.871	0.390	50.68	Refer	Carbon	1.000
4	12.220	291.768	0.013	2.82	Refer	Hydrogen	0.152
	Total		0.866	63.99			

CHN for $C_{18}H_{14}ClN_3O_2$ (**3h**)

Fig S9. 5-(4-chlorophenyl)-N-(naphthalen-1-yl)-3-nitropyridin-2-amine (**3i**)

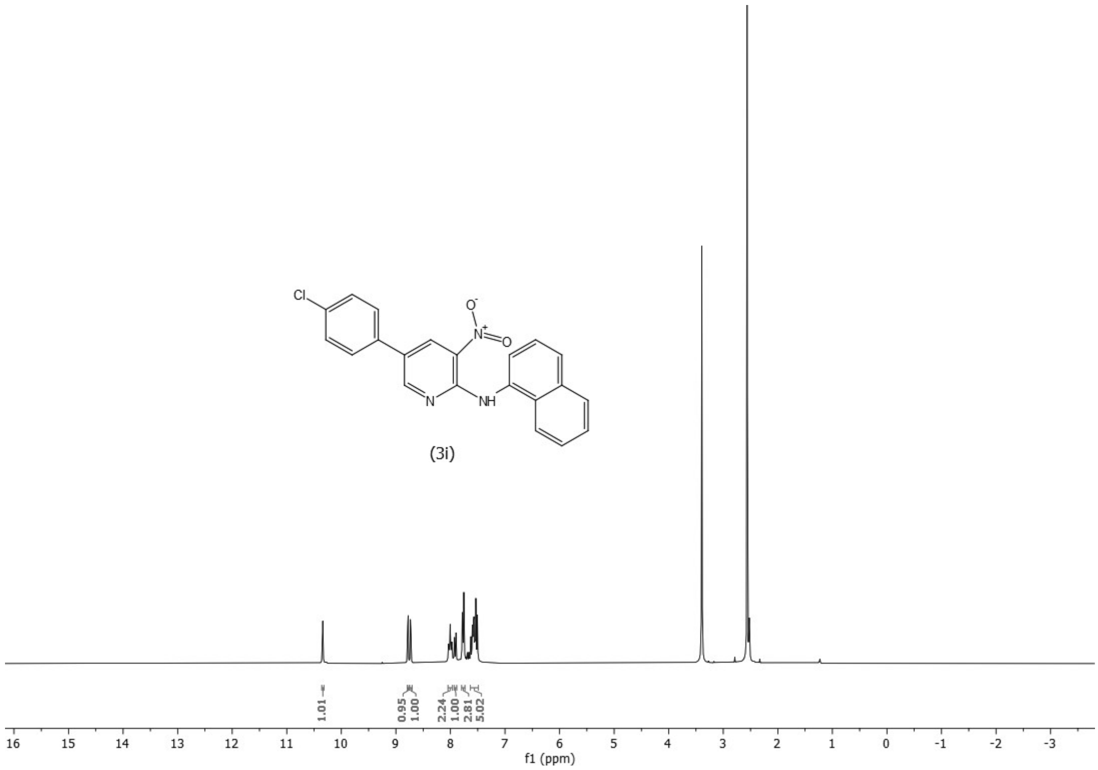

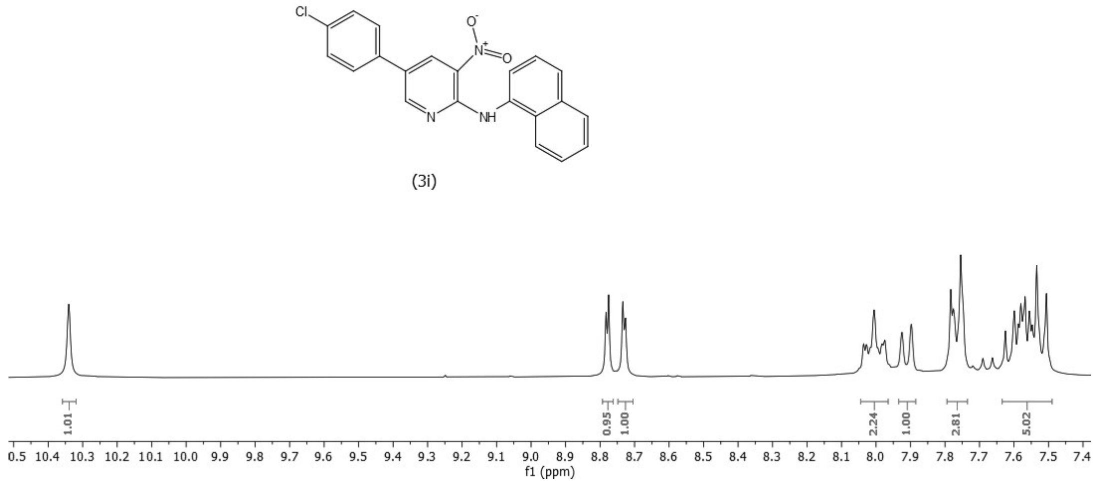

Fig S9. 5-(4-chlorophenyl)-N-(naphthalen-1-yl)-3-nitropyridin-2-amine (**3i**)

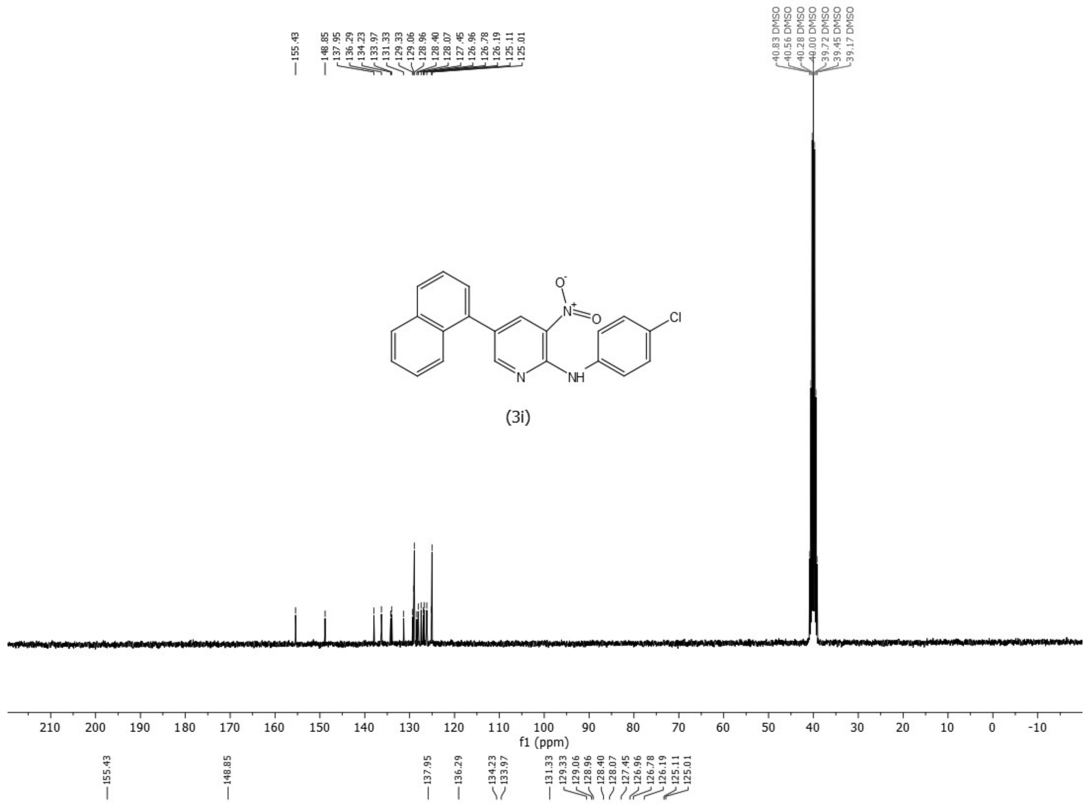

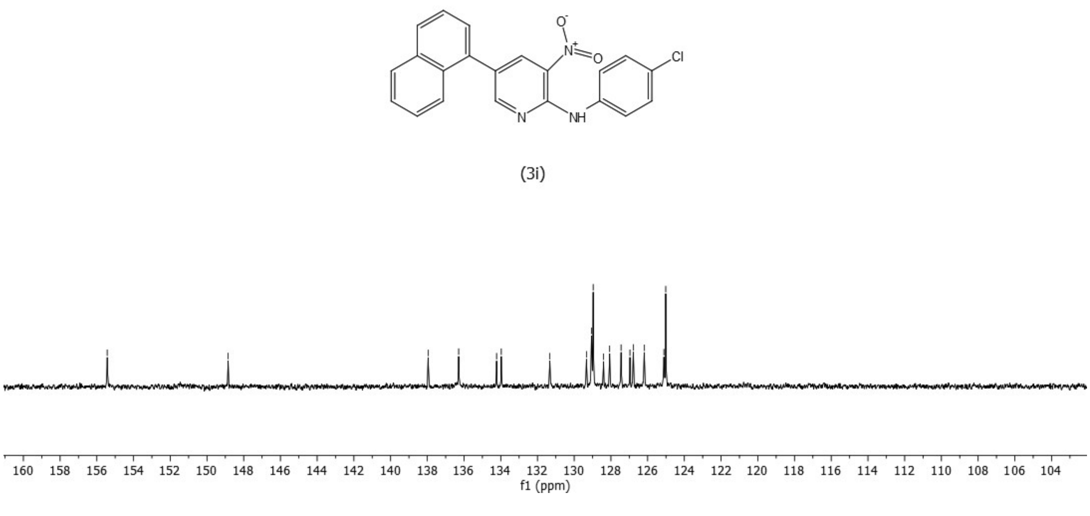

Fig S9. 5-(4-chlorophenyl)-N-(naphthalen-1-yl)-3-nitropyridin-2-amine (**3i**)

Chromatogram C:\EAS Clarity\WORK1\Data\k18 1402.5.16_1_3_2002 5_22_03 AM_076.PRM
Result Table (ESTD - k18 1402.5.16_1_3_2002 5_22_03 AM_076 - INT7 - 1)

	Reten. Time [min]	Response	Weight [mg]	Weight [%]	Peak Type	Element Name	Carbon Response Ratio
1	1.377	335.446	0.145	11.38	Refer	Nitrogen	0.075
3	2.673	4855.879	0.917	67.49	Refer	Carbon	1.000
4	12.390	771.553	0.045	3.60	Refer	Hydrogen	0.163
	Total		1.384	82.47			

CHN for $C_{21}H_{14}ClN_3O_2$ (**3i**)

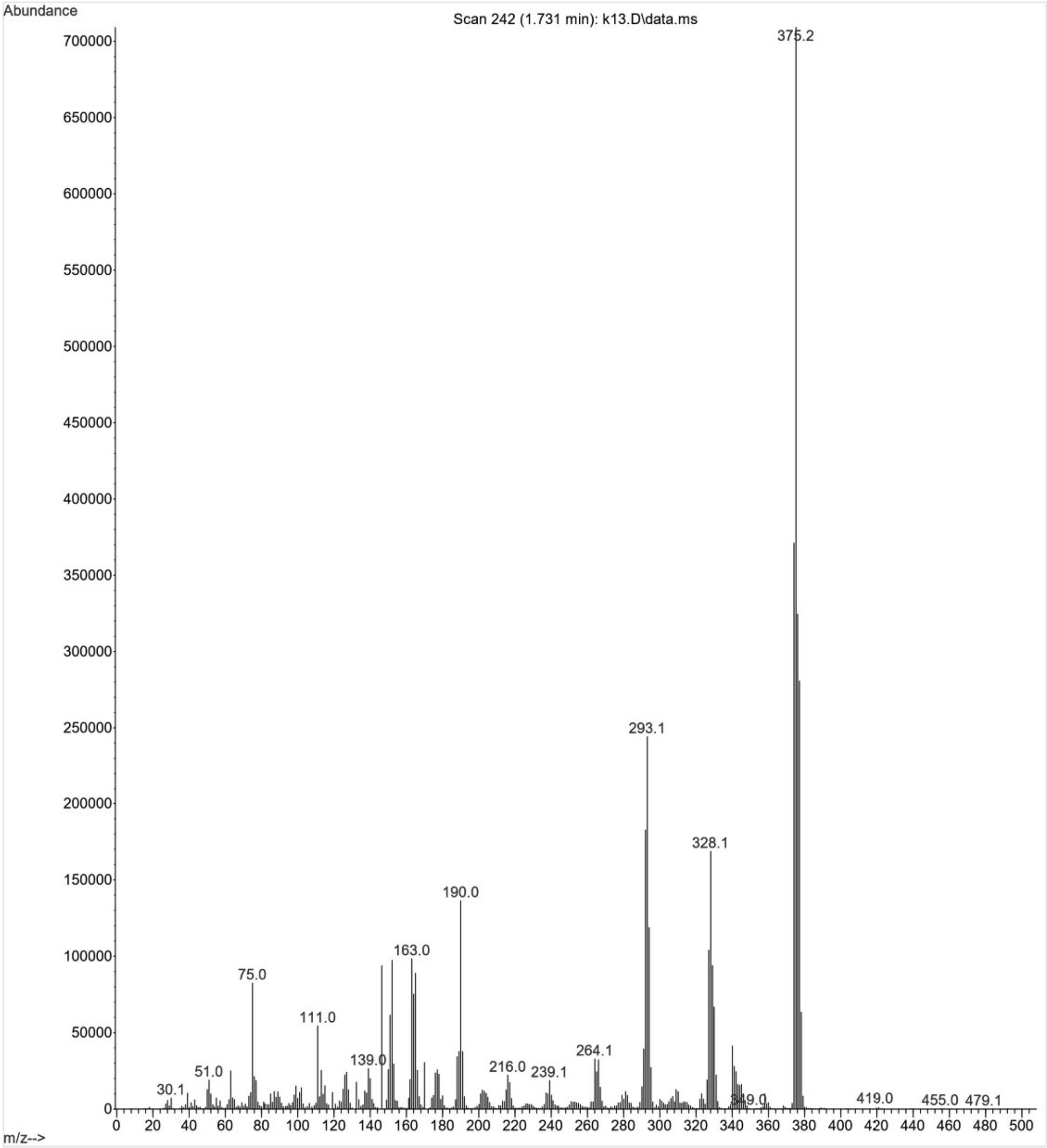

$C_{21}H_{14}ClN_3O_2$ (**3i**)

MS (*m/z*): 375

Fig S10. 5-(4-bromophenyl)-N-(4-methoxyphenyl)-3-nitropyridin-2-amine (**3j**)

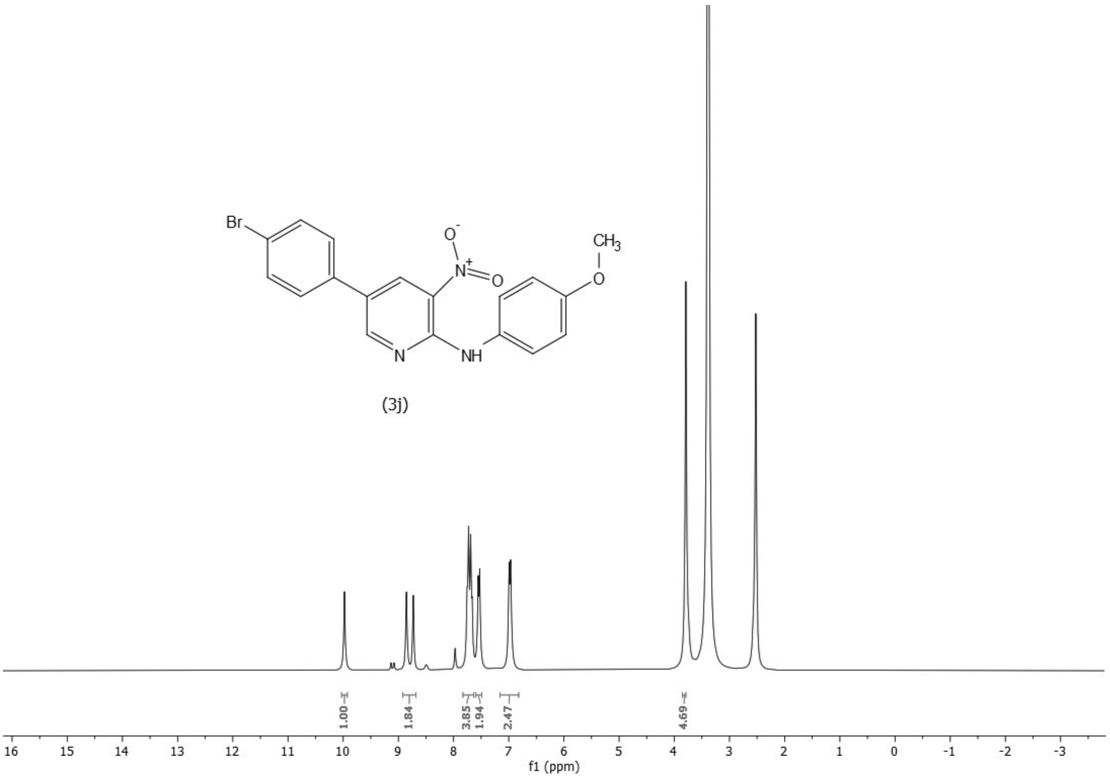

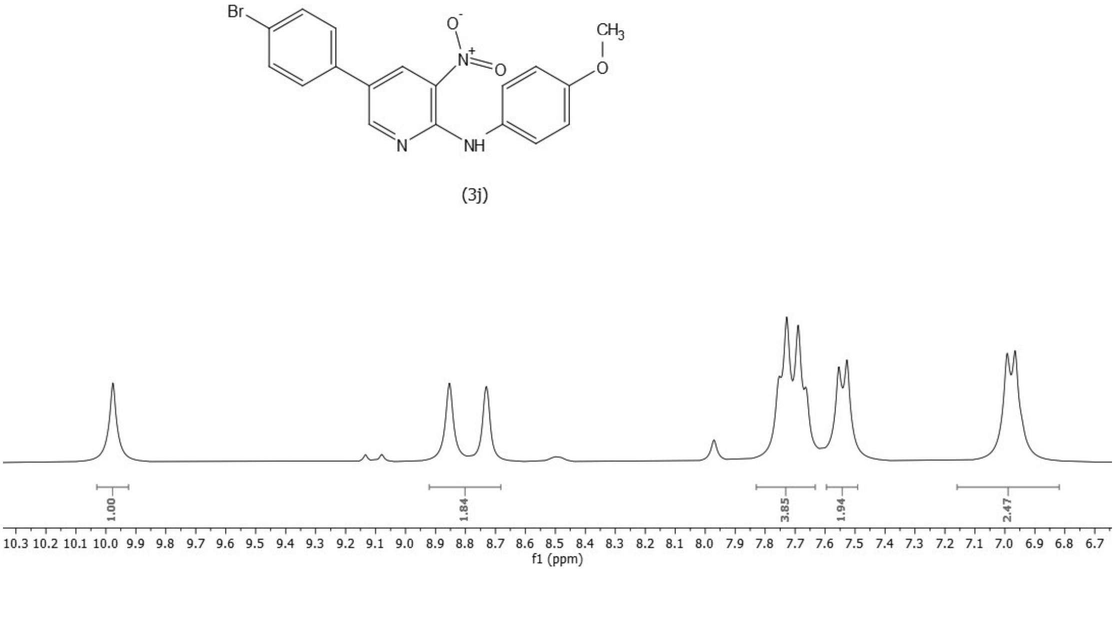

Fig S10. 5-(4-bromophenyl)-N-(4-methoxyphenyl)-3-nitropyridin-2-amine (**3j**)

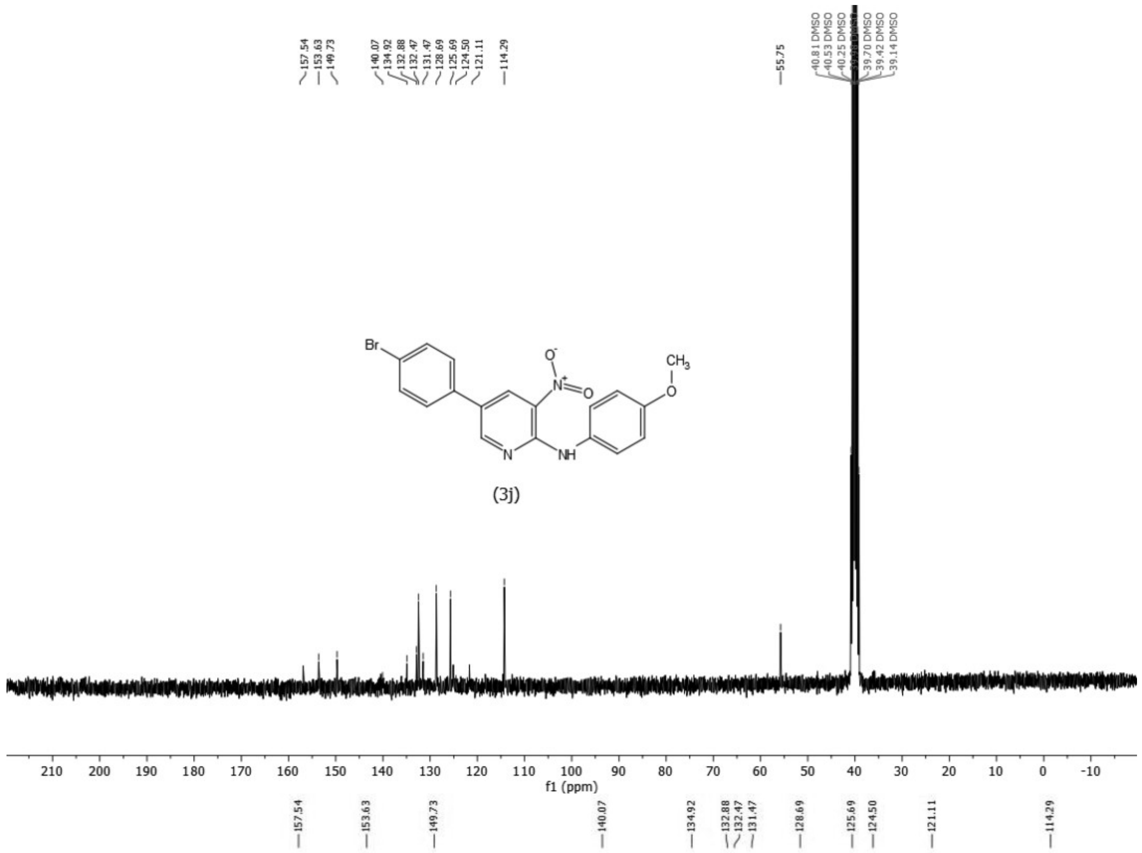

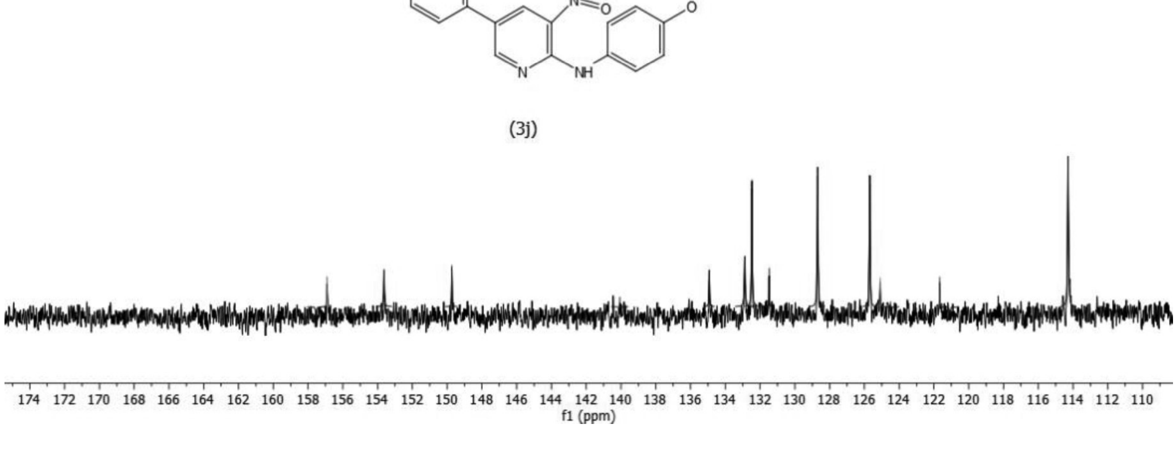

Fig S10. 5-(4-bromophenyl)-N-(4-methoxyphenyl)-3-nitropyridin-2-amine (**3j**)

Chromatogram C:\EAS Clarity\WORK1\Data\k23 1402.5.16_1_3_2002 2_22_02 AM_070.PRM
Result Table (ESTD - k23 1402.5.16_1_3_2002 2_22_02 AM_070 - INT7 - 1)

	Reten. Time [min]	Response	Weight [mg]	Weight [%]	Peak Type	Element Name	Carbon Response Ratio
1	1.327	639.469	0.306	12.47	Refer	Nitrogen	0.087
3	2.477	7328.979	1.470	59.33	Refer	Carbon	1.000
4	10.917	1058.055	0.063	3.56	Refer	Hydrogen	0.165
	Total		32.307	75.36			

CHN for $C_{18}H_{14}BrN_3O_3$ (**3j**)

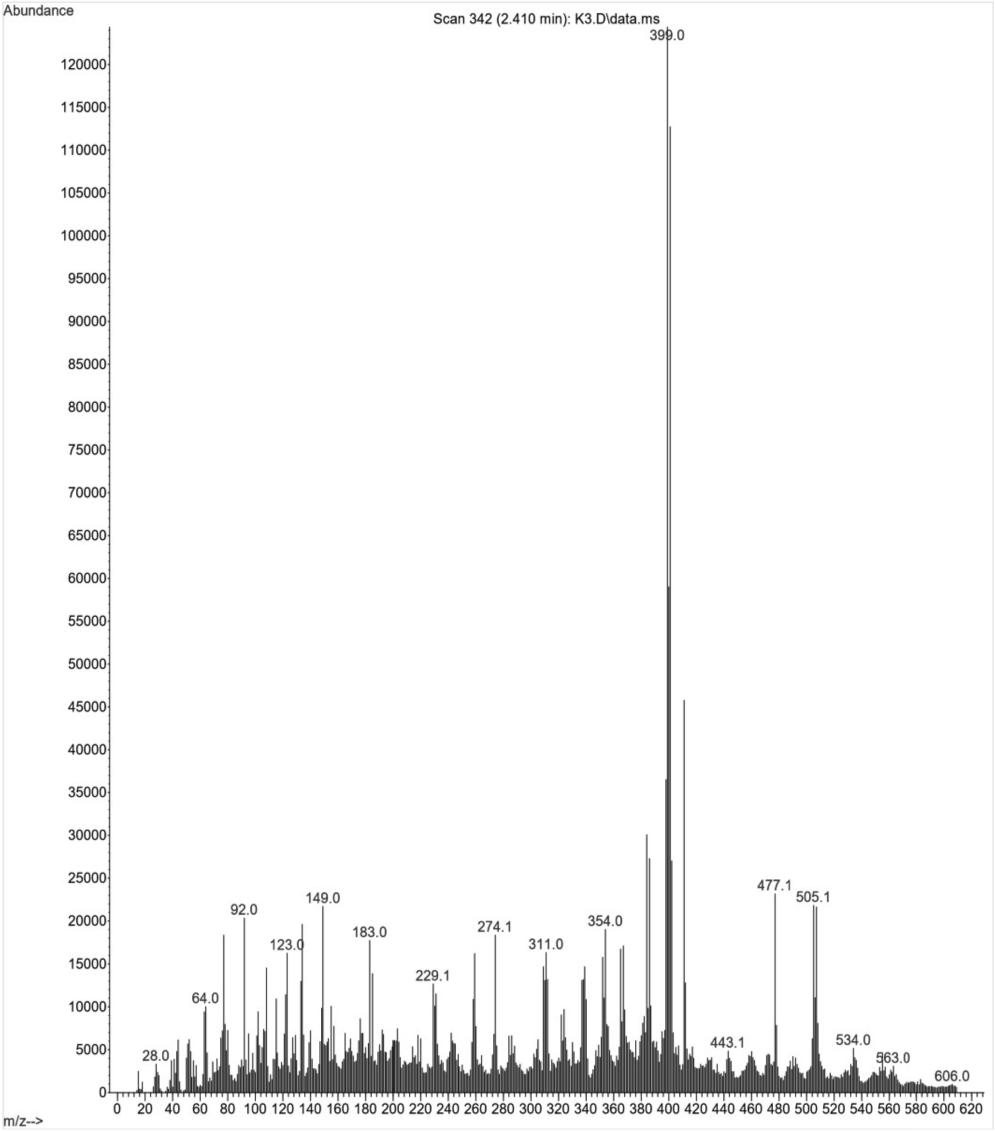

$C_{18}H_{14}BrN_3O_3$ (**3j**)

MS (*m/z*): 399

Fig S11. 5-(4-bromophenyl)-N-(4-chlorophenyl)-3-nitropyridin-2-amine (**3k**)

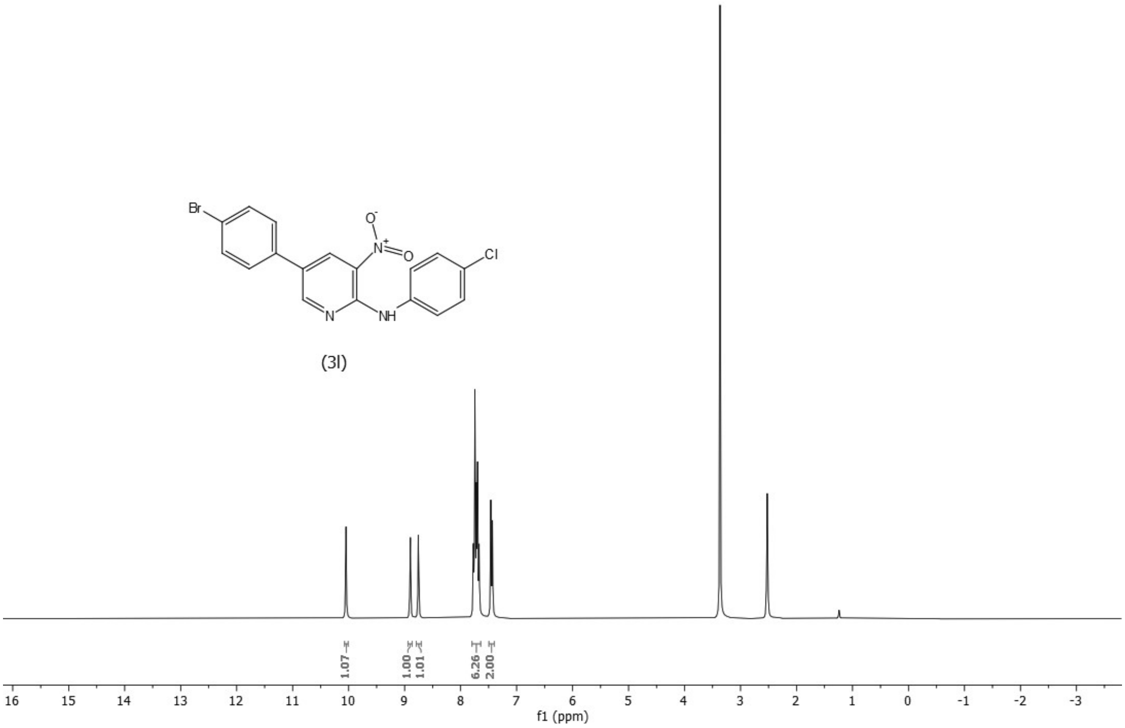

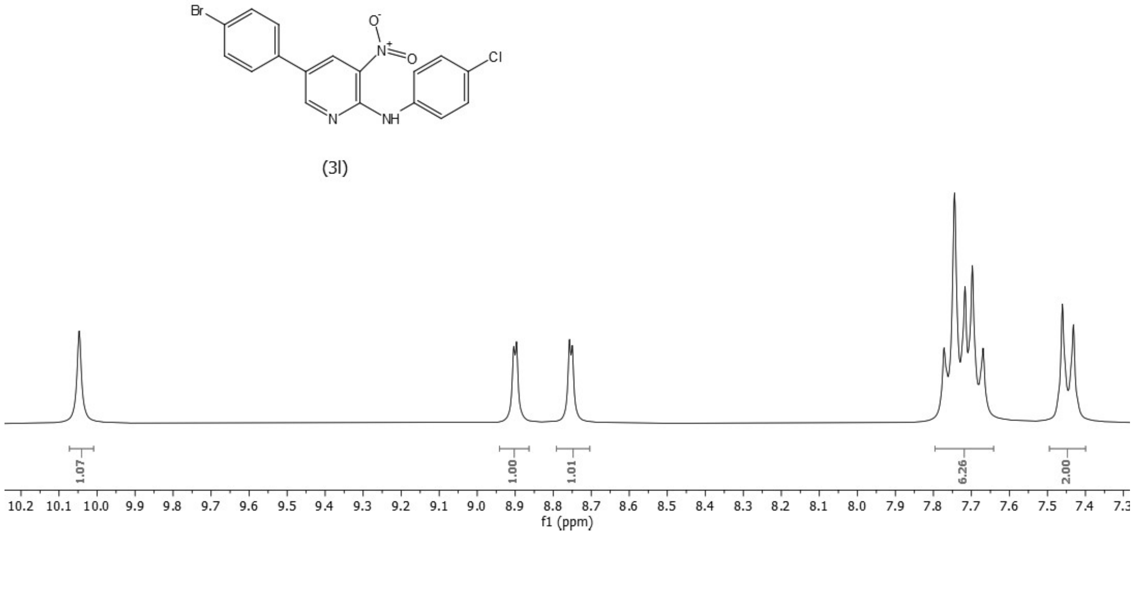

Fig S11. 5-(4-bromophenyl)-N-(4-chlorophenyl)-3-nitropyridin-2-amine (**3k**)

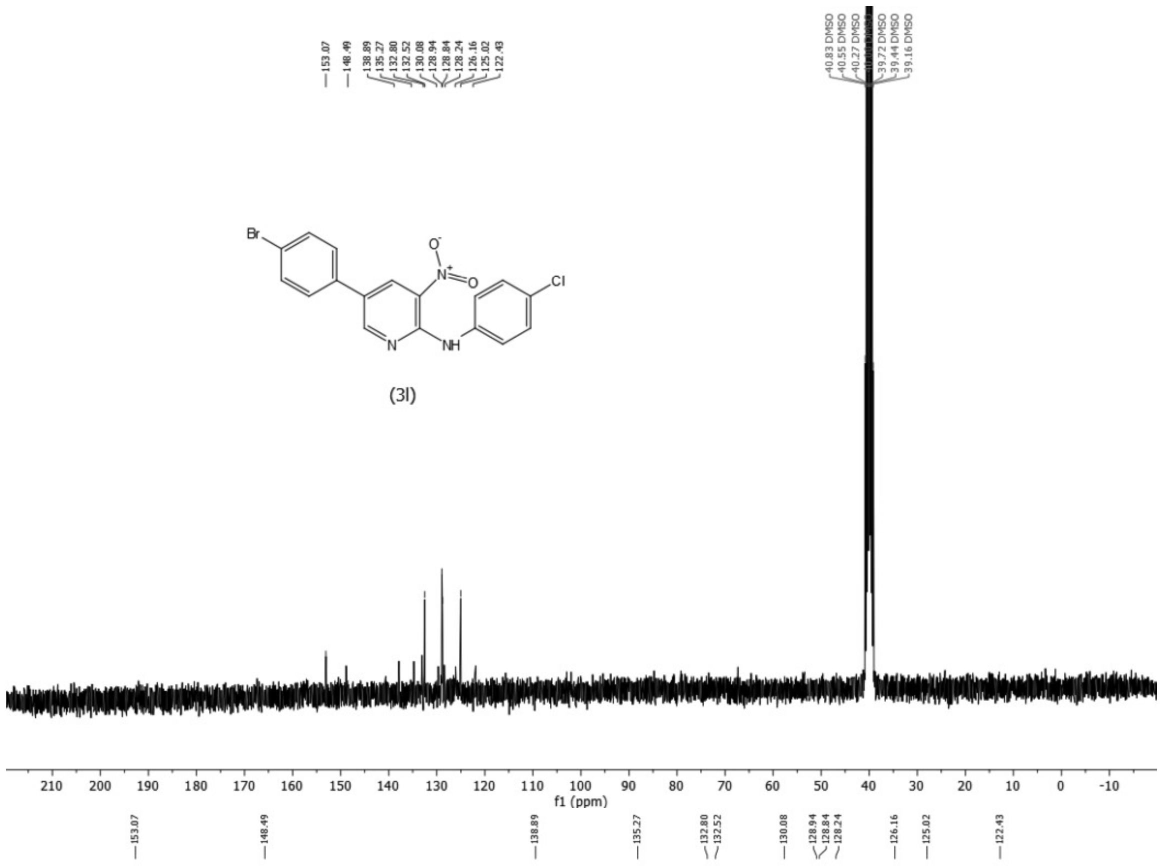

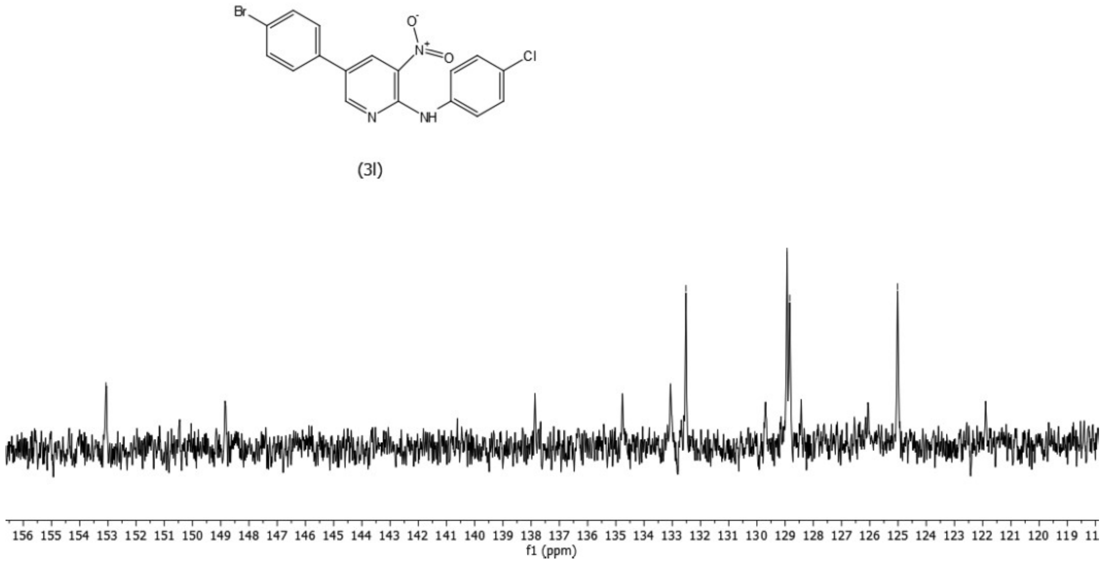

Fig S11. 5-(4-bromophenyl)-N-(4-chlorophenyl)-3-nitropyridin-2-amine (**3k**)

Chromatogram C:\EAS Clarity\Work1\DATA\k5 1402.5.15_1_2_2002 3_54_00 AM_061.PRM
Result Table (ESTD – k5 1402.5.15_1_2_2002 3_54_00 AM_061 - INT7 - 1)

	Reten. Time [min]	Response	Weight [mg]	Weight [%]	Peak Type	Element Name	Carbon Response Ratio
1	1.317	878.660	0.433	13.68	Refer	Nitrogen	0.092
3	2.563	4114.128	0.761	67.75	Refer	Carbon	1.000
4	10.740	1035.756	0.062	4.33	Refer	Hydrogen	0.176
	Total		1.858	85.76			

CHN for $C_{17}H_{11}BrClN_3O_2$ (**3k**)

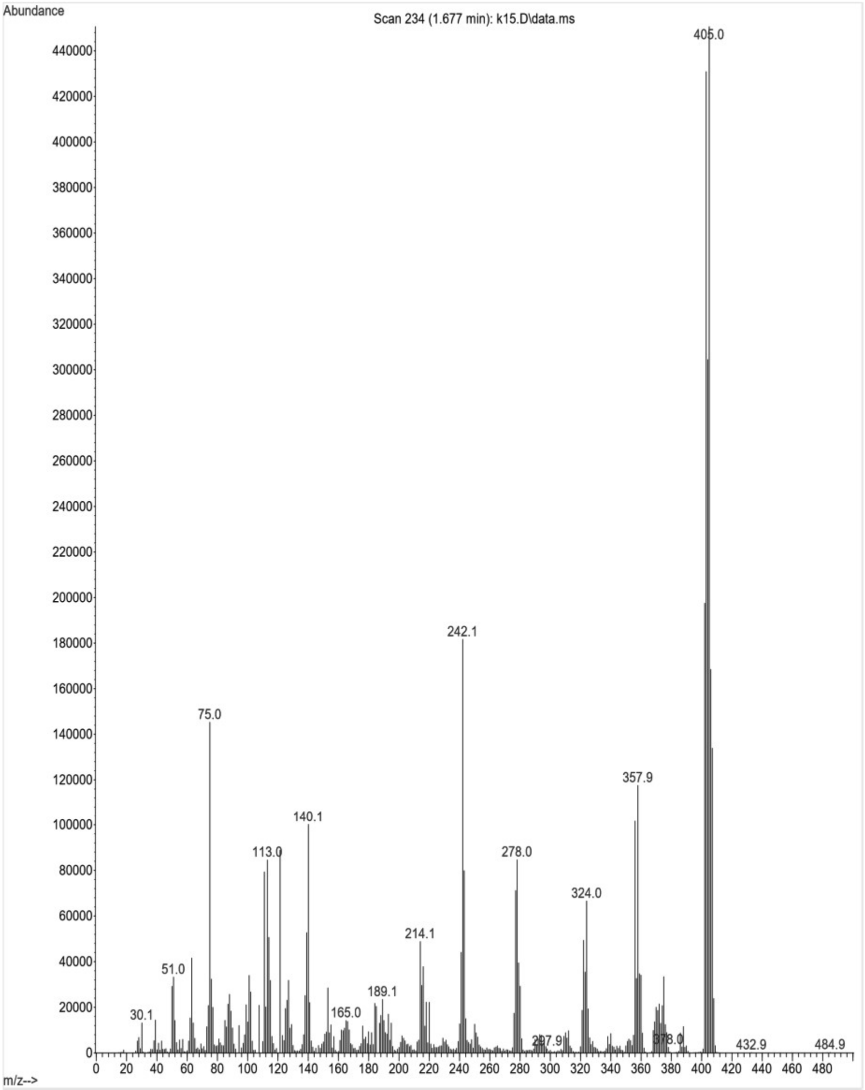

$C_{17}H_{11}BrClN_3O_2$ (**3k**)

MS (*m/z*): 405

Fig S12. *5-(4-bromophenyl)-3-nitro-N-(p-tolyl)pyridin-2-amine* (**3l**)

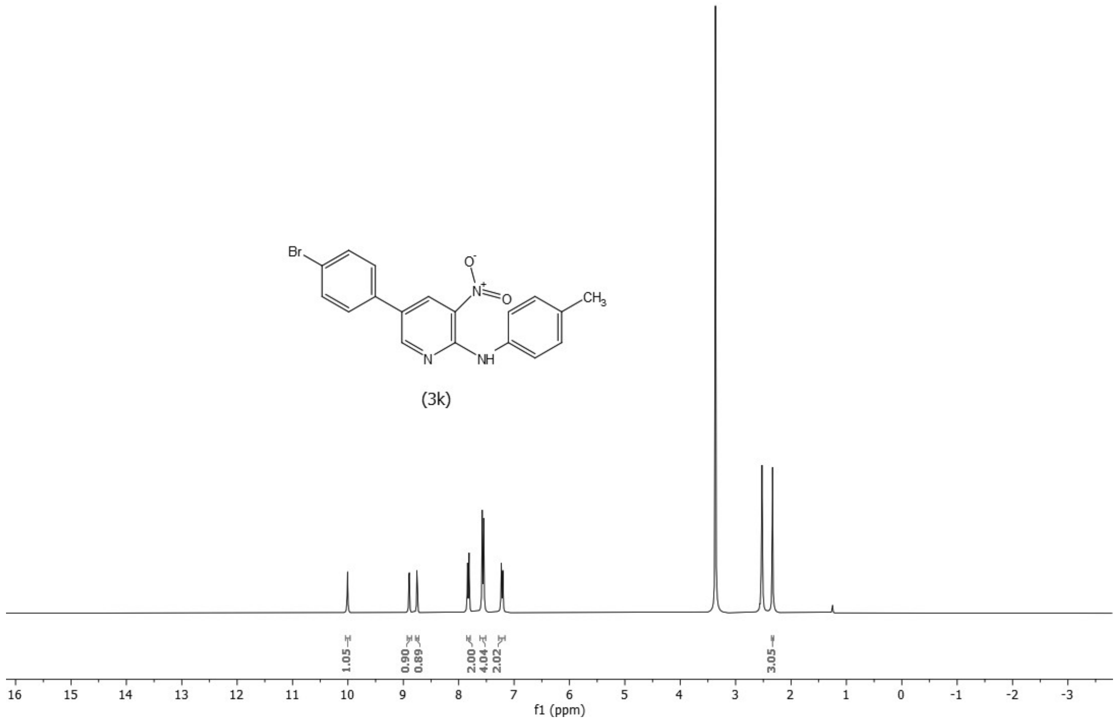

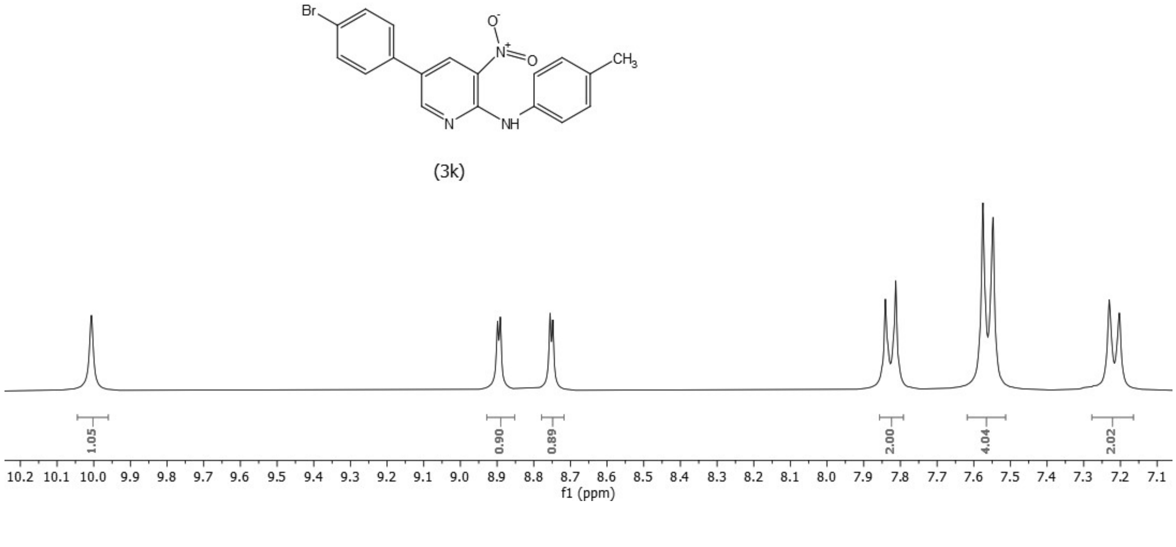

Fig S12. *5-(4-bromophenyl)-3-nitro-N-(p-tolyl)pyridin-2-amine* (**3l**)

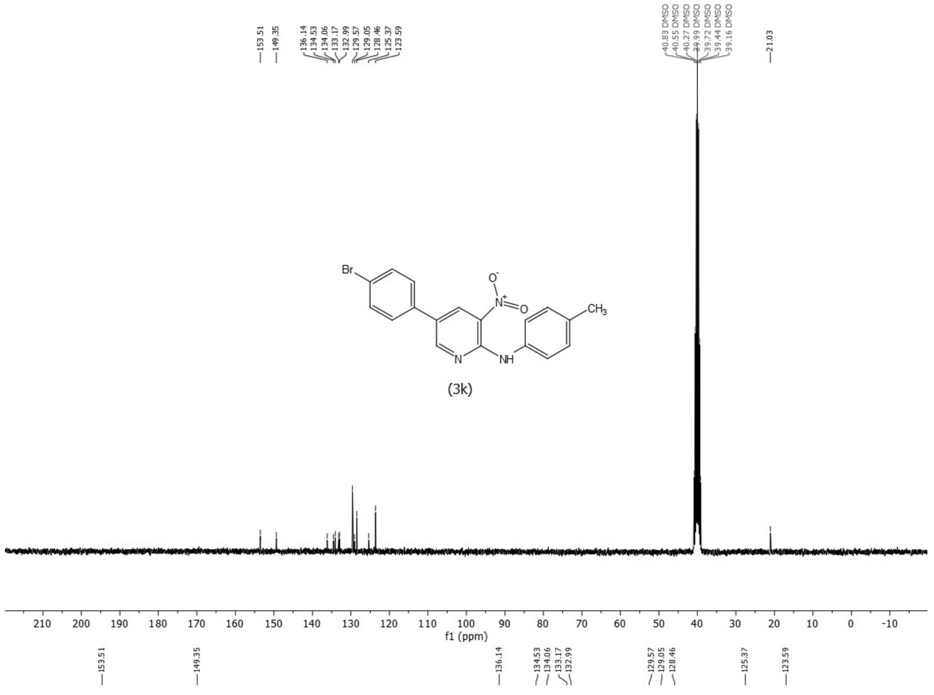

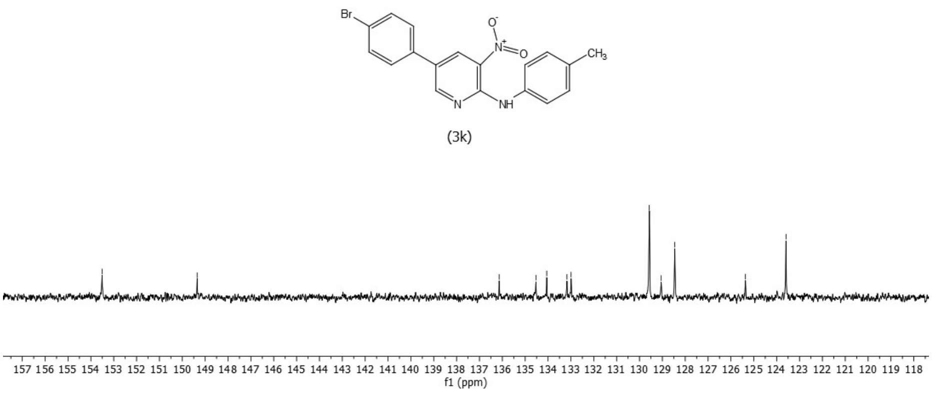

Chromatogram C:\EAS Clarity\Work1\DATA\k2 1402.5.16_1_3_2002 4_22_03 AM_074.PRM

Result Table (ESTD – k2 1402.5.16_1_3_2002 4_22_03 AM_074 - INT7 - 1)

	Reten. Time [min]	Response	Weight [mg]	Weight [%]	Peak Type	Element Name	Carbon Response Ratio
1	1.360	348.176	0.152	11.13	Refer	Nitrogen	0.106
3	2.687	3298.772	0.593	49.95	Refer	Carbon	1.000
4	12.457	653.888	0.037	4.39	Refer	Hydrogen	0.198
	Total		0.782	65.47			

CHN for $C_{18}H_{14}BrN_3O_2$ (**3l**)

Fig S13. 4-(tert-butyl)-6'-((4-chlorophenyl)amino)-5'-nitro-[1,3'-bipyridin]-1-ium perchlorate (**3m**)

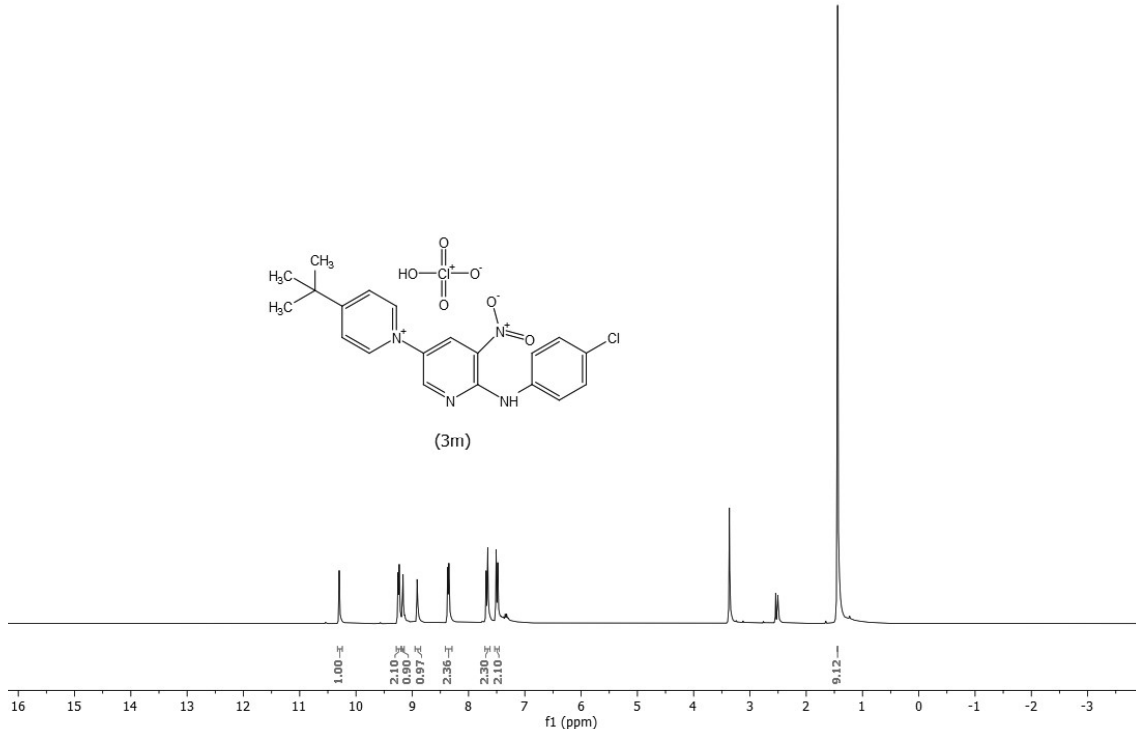

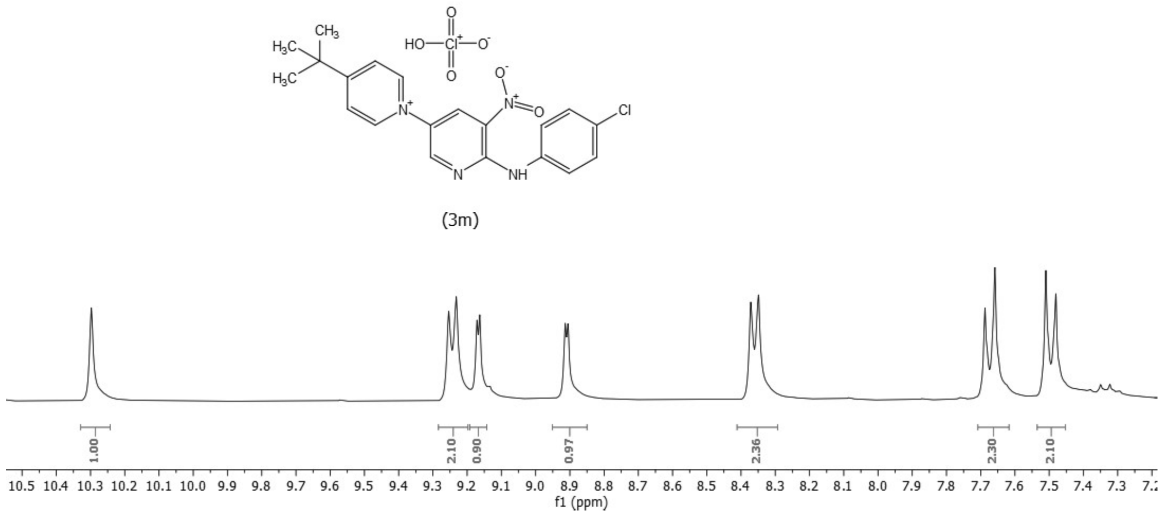

Fig S13. 4-(tert-butyl)-6'-((4-chlorophenyl)amino)-5'-nitro-[1,3'-bipyridin]-1-ium perchlorate (**3m**)

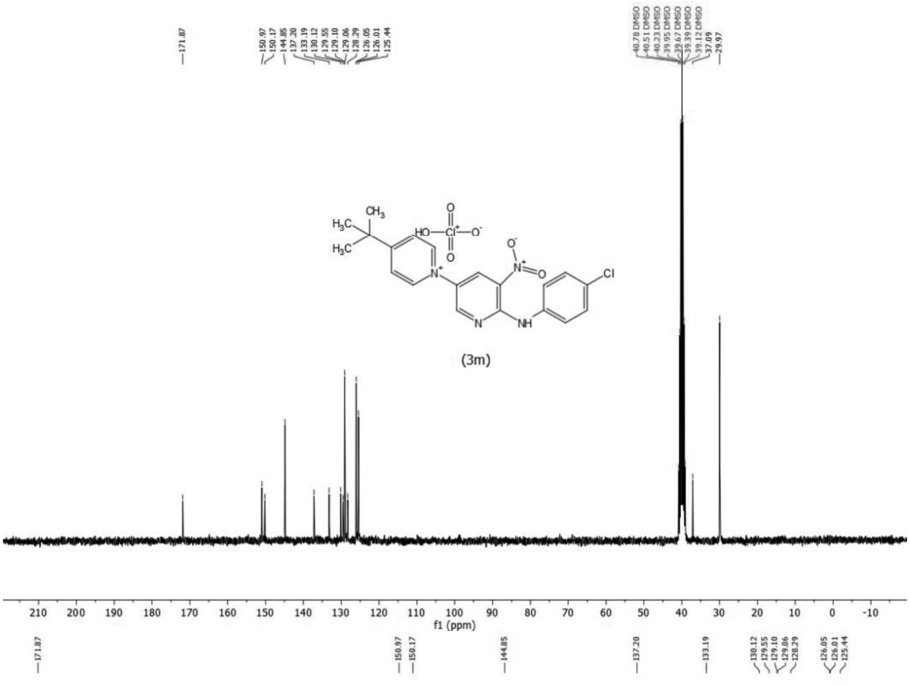

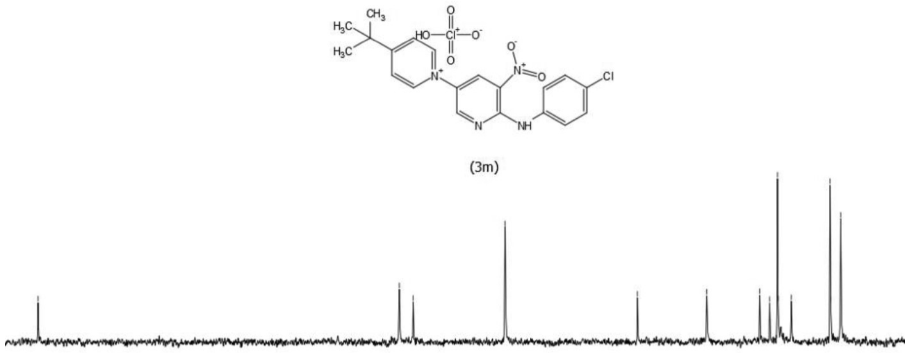

Chromatogram C:\EAS Clarity\Work1\DATA\k6 1402.5.16_1_3_2002 2_52_02 AM_071.PRM
Result Table (ESTD - k6 1402.5.16_1_3_2002 2_52_02 AM_071 - INT7 - 1)

	Reten. Time [min]	Response	Weight [mg]	Weight [%]	Peak Type	Element Name	Carbon Response Ratio
1	1.337	748.925	0.433	11.68	Refer	Nitrogen	0.072
3	2.453	11794.093	0.761	71.96	Refer	Carbon	1.000
4	10.163	20.49.761	0.062	4.47	Refer	Hydrogen	0.172
	Total		1.858	85.76			

CHN for $C_{21}H_{20}Cl_2N_3O_6$ (**3m**)

Fig S14. 5-(naphthalen-1-yl)-3-nitro-N-(o-tolyl)pyridin-2-amine (**3n**)

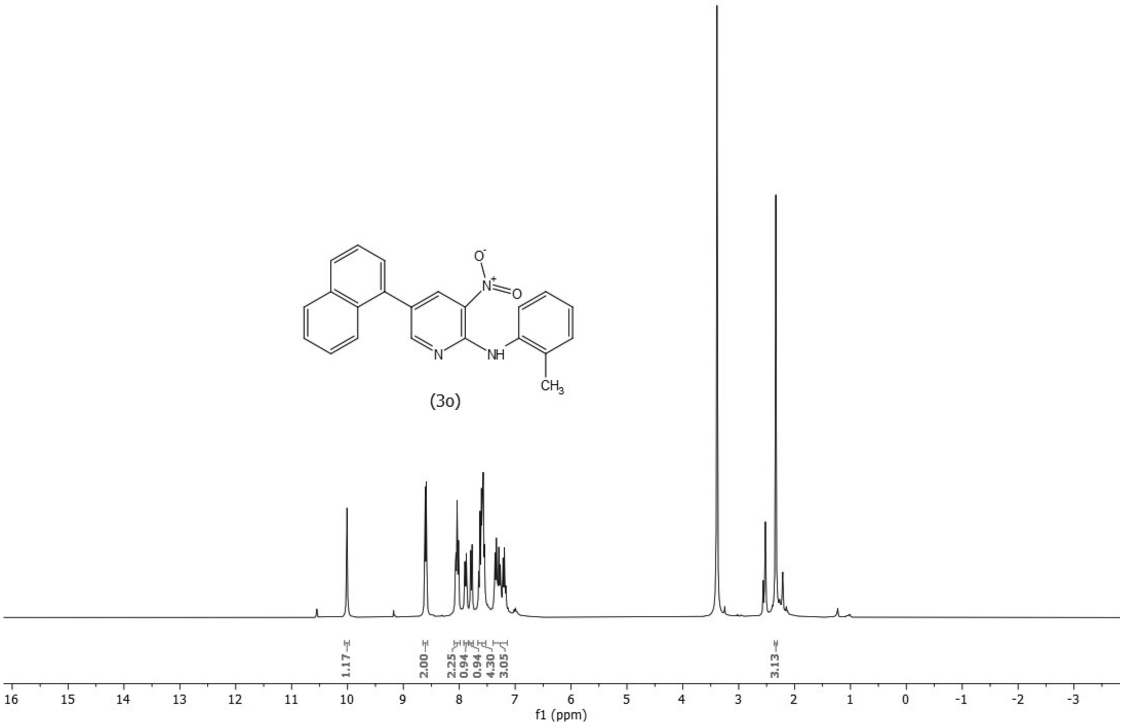

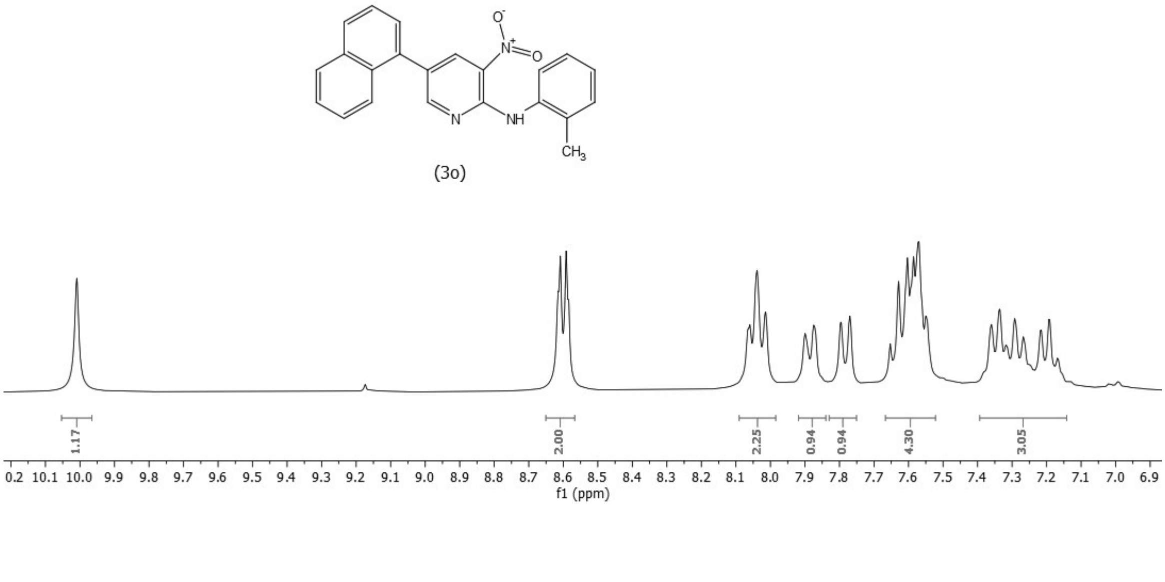

Fig S14. 5-(naphthalen-1-yl)-3-nitro-N-(o-tolyl)pyridin-2-amine (**3n**)

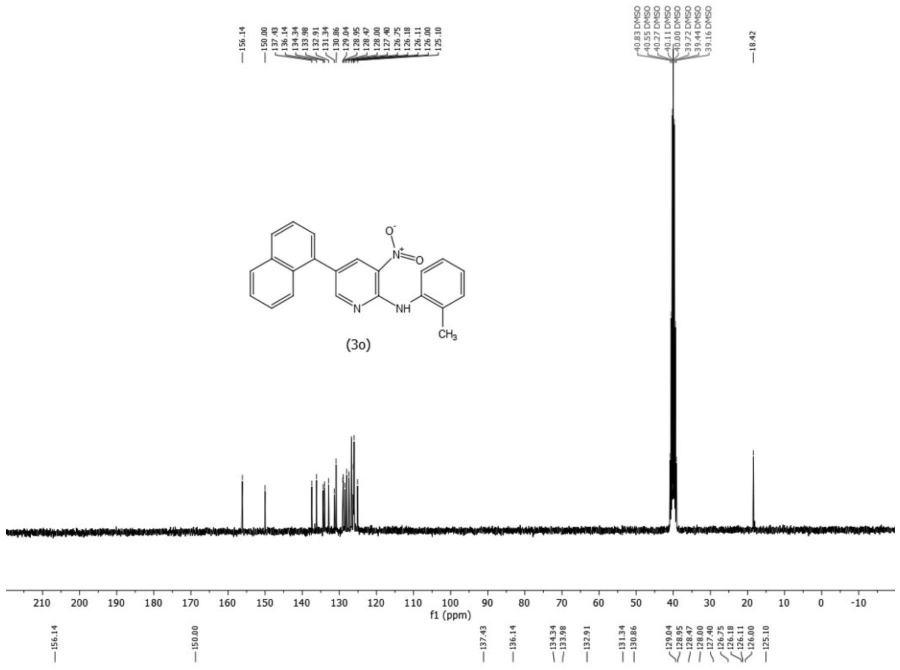

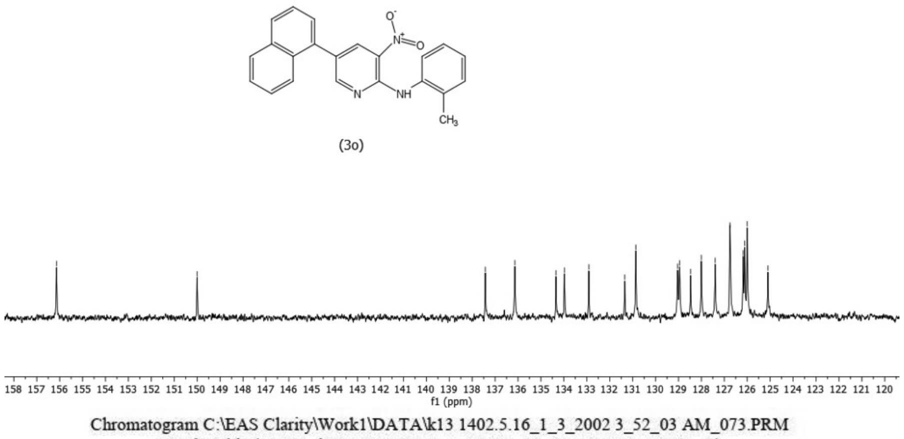

Chromatogram C:\EAS Clarity\Work1\DATA\k13 1402.5.16_1_3_2002 3_52_03 AM_073.PRM
Result Table (ESTD - k13 1402.5.16_1_3_2002 3_52_03 AM_073 - INT7 - 1)

	Reten. Time [min]	Response	Weight [mg]	Weight [%]	Peak Type	Element Name	Carbon Response Ratio
1	1.353	411.904	0.186	11.52	Refer	Nitrogen	0.081
3	2.607	5992.995	1.165	67.61	Refer	Carbon	1.000
4	11.567	762.341	0.044	3.45	Refer	Hydrogen	0.160
	Total		3.062	82.58			

CHN for $C_{22}H_{17}N_3O_2$ (**3n**)

Fig S15. 5-(naphthalen-1-yl)-3-nitro-N-(p-tolyl)pyridin-2-amine (**3o**)

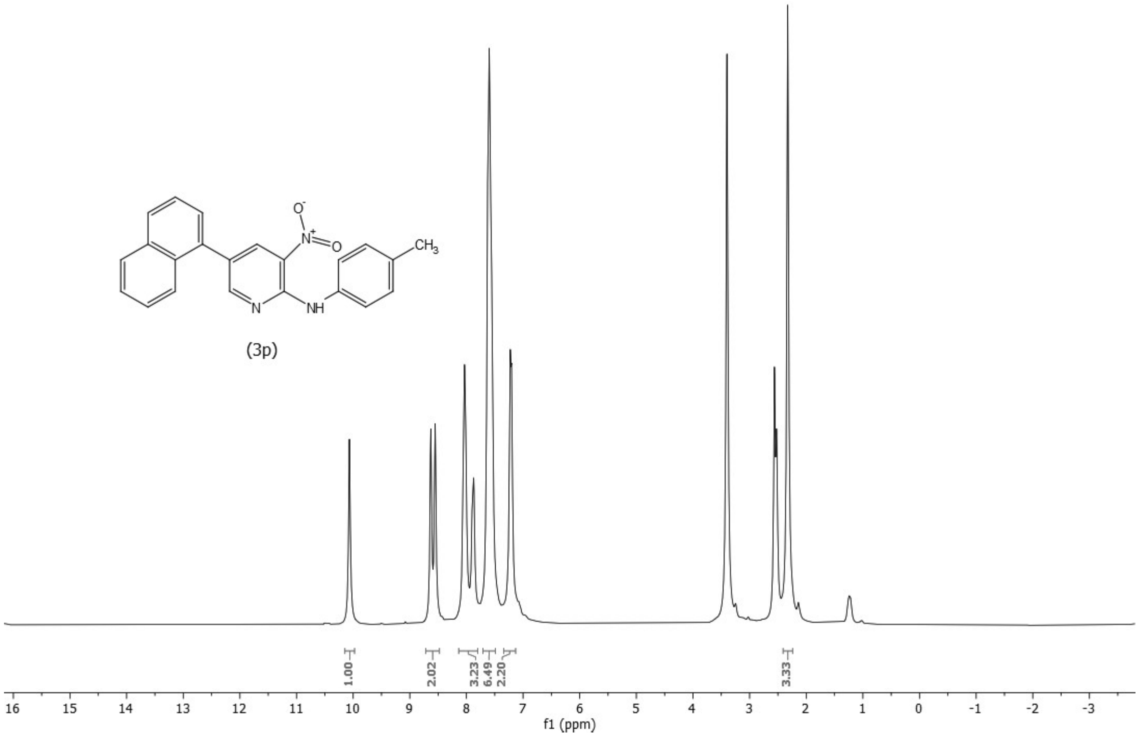

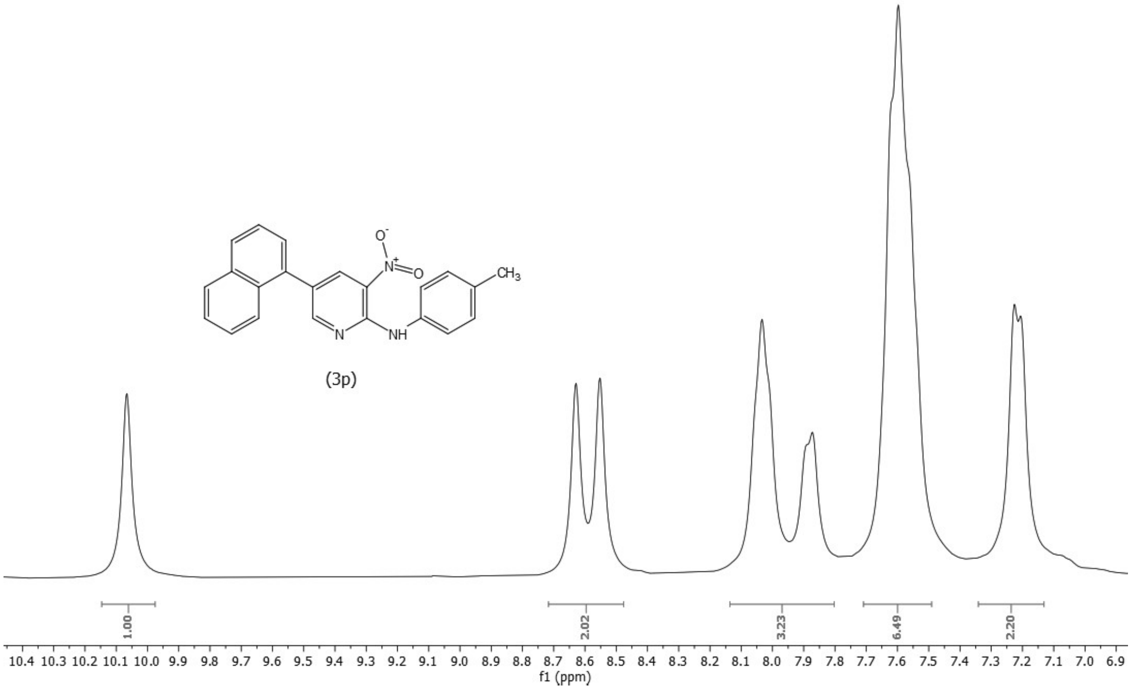

Fig S15. 5-(naphthalen-1-yl)-3-nitro-N-(p-tolyl)pyridin-2-amine (**3o**)

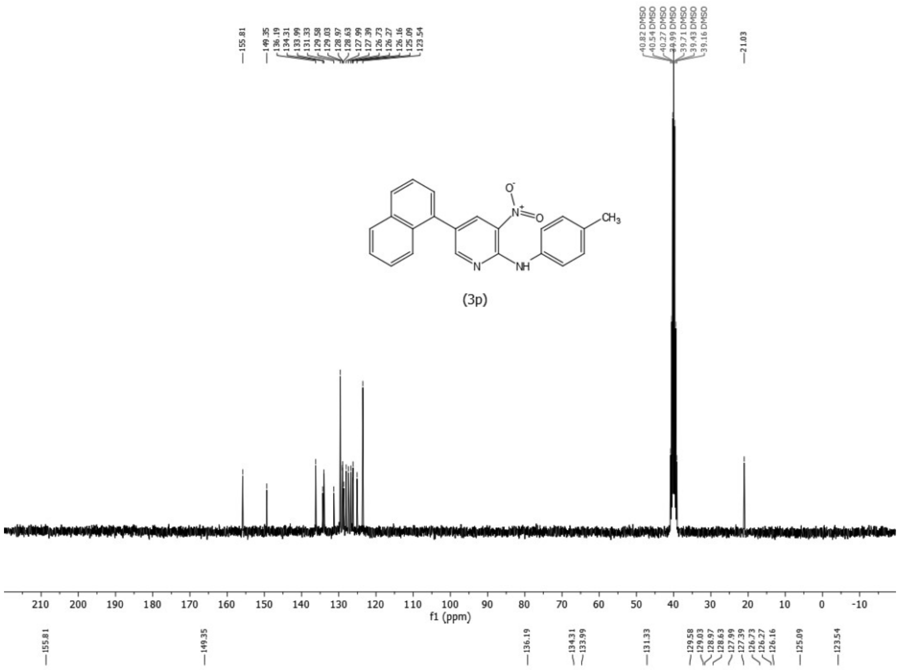

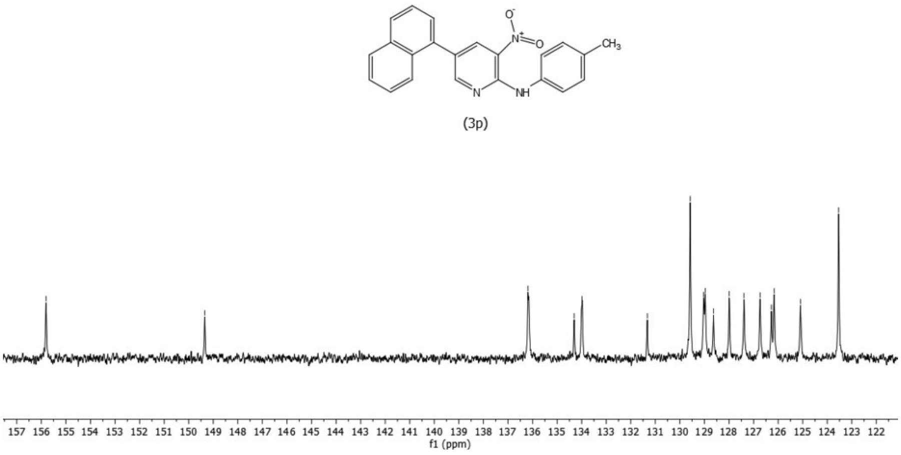

Chromatogram C:\EAS Clarity\WORK1\Data\k16 1402.5.16_1_3_2002 5_52_03 AM_077.PRM

Result Table (ESTD - k16 1402.5.16_1_3_2002 5_52_03 AM_077 - INT7 - 1)

	Reten. Time [min]	Response	Weight [mg]	Weight [%]	Peak Type	Element Name	Carbon Response Ratio
1	1.373	787.064	0.385	12.96	Refer	Nitrogen	0.086
3	2.560	4126.818	0.763	62.69	Refer	Carbon	1.000
4	10.560	1098.419	0.066	3.67	Refer	Hydrogen	0.171
	Total		1.879	79.32			

CHN for $C_{22}H_{17}N_3O_2$ (**3o**)

Fig S16. N-(4-chlorophenyl)-5-(naphthalen-1-yl)-3-nitropyridin-2-amine (**3p**)

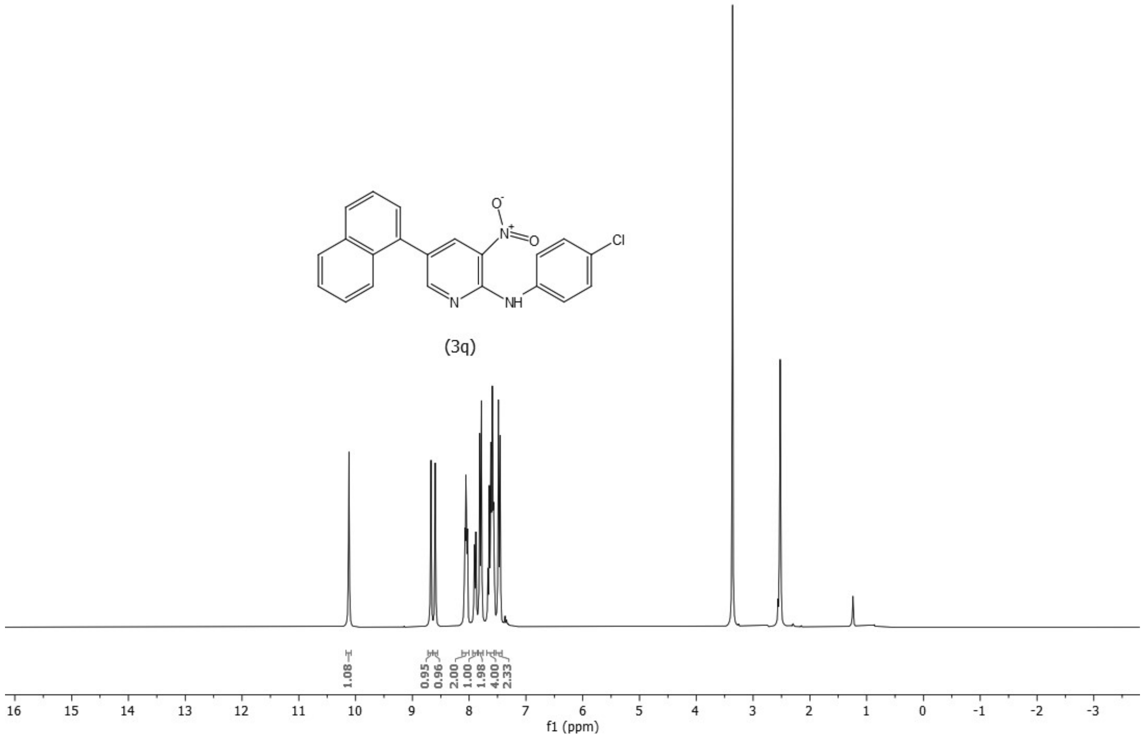

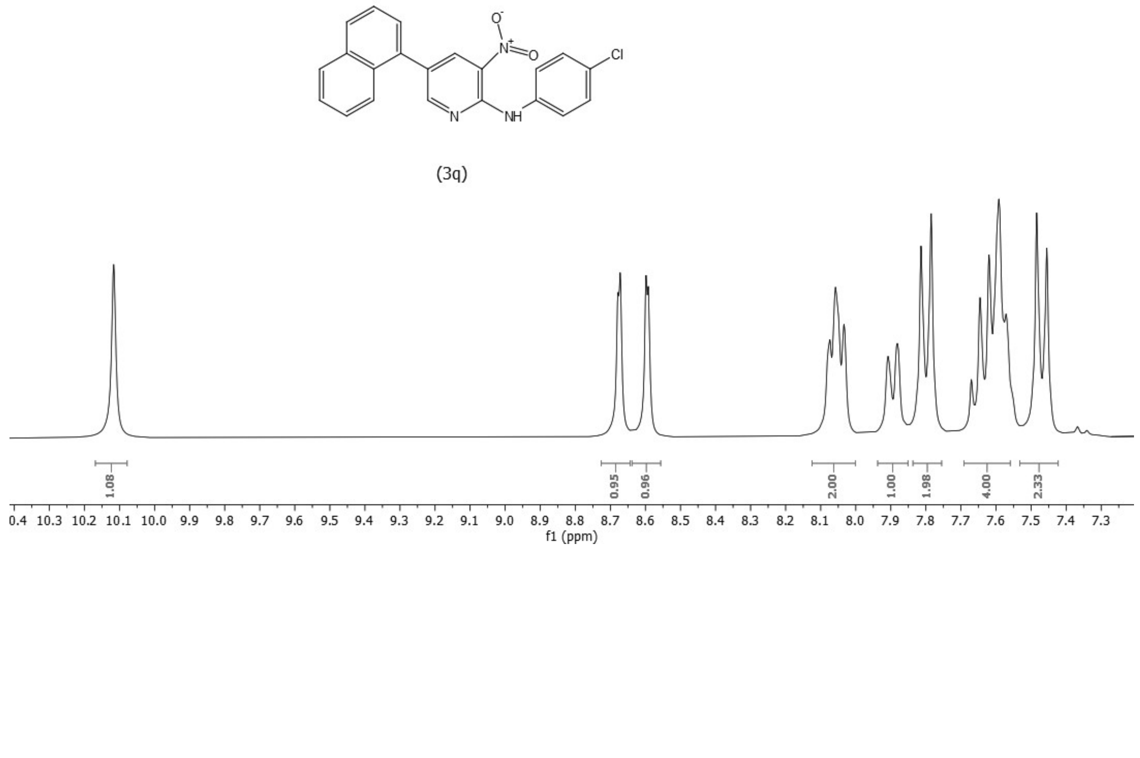

Fig S16. N-(4-chlorophenyl)-5-(naphthalen-1-yl)-3-nitropyridin-2-amine (**3p**)

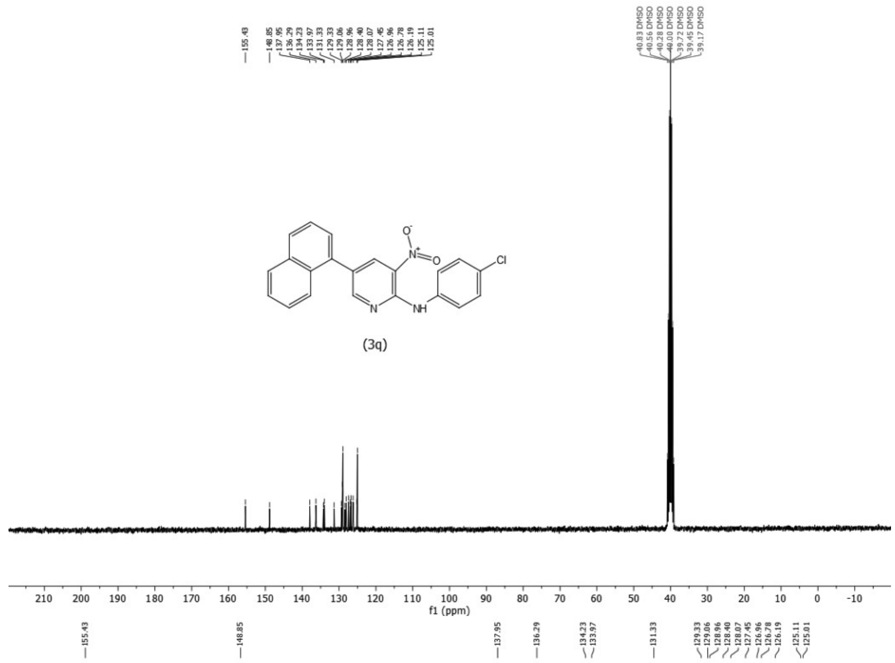

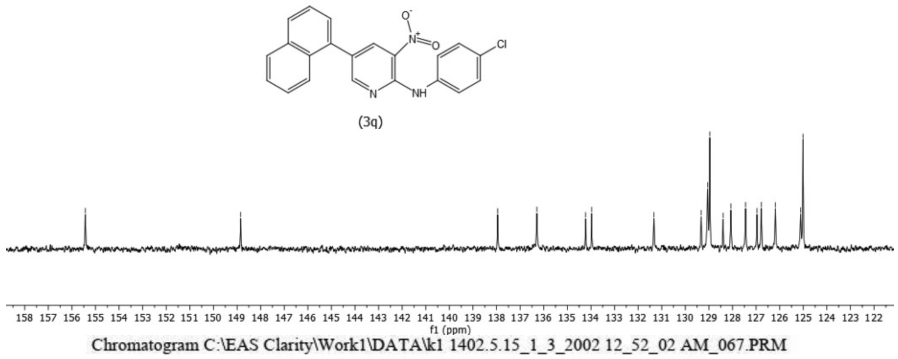

Chromatogram C:\EAS Clarity\Work1\DATA\k1 1402.5.15_1_3_2002 12_52_02 AM_067.PRM

Result Table (ESTD - k1 1402.5.15_1_3_2002 12_52_02 AM_067 - INT7 - 1)

	Reten. Time [min]	Response	Weight [mg]	Weight [%]	Peak Type	Element Name	Carbon Response Ratio
1	1.307	976.438	0.485	11.09	Refer	Nitrogen	0.077
3	2.350	12688.541	2.927	74.85	Refer	Carbon	1.000
4	10.150	2718.823	0.172	4.62	Refer	Hydrogen	0.214
	Total		3.805	90.56			

CHN for $C_{21}H_{14}ClN_3O_2$ (**3p**)

Fig S17. N,5-di(naphthalen-1-yl)-3-nitropyridin-2-amine (**3q**)

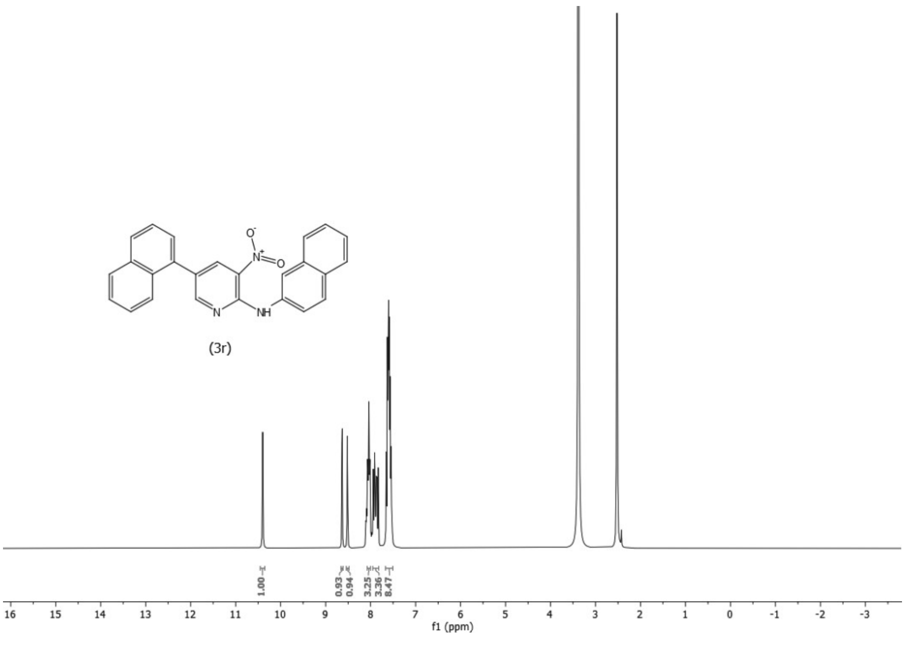

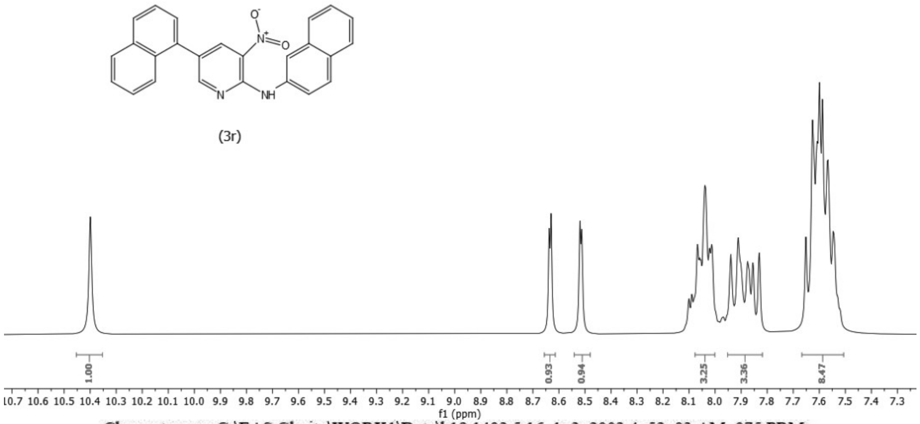

Chromatogram C:\EAS Clarity\WORK1\Data\k19 1402.5.16_1_3_2002 4_52_03 AM_075.PRM
Result Table (ESTD - k19 1402.5.16_1_3_2002 4_52_03 AM_075 - INT7 - 1)

	Reten. Time [min]	Response	Weight [mg]	Weight [%]	Peak Type	Element Name	Carbon Response Ratio
1	1.383	733.824	0.356	13.02	Refer	Nitrogen	0.092
3	2.540	11313.187	2.506	62.45	Refer	Carbon	1.000
4	10.597	2275.748	0.143	3.56	Refer	Hydrogen	0.170
	Total		3.063	79.03			

CHN for $C_{25}H_{17}N_3O_2$ (**3q**)
